# Extra-Mediterranean glacial refuges in barred and common grass snakes *(Natrix helvetica*, *N. natrix)*

**DOI:** 10.1038/s41598-018-20218-2

**Published:** 2018-01-29

**Authors:** Carolin Kindler, Eva Graciá, Uwe Fritz

**Affiliations:** 1 Museum of Zoology (Museum für Tierkunde), Senckenberg Dresden, A. B. Meyer Building, 01109 Dresden, Germany; 2 Ecology Area, Department of Applied Biology, Miguel Hernández University, Av. de la Universidad, Torreblanca, 03202 Elche, Spain

## Abstract

Extra-Mediterranean glacial refugia of thermophilic biota, in particular in northern latitudes, are controversial. In the present study we provide genetic evidence for extra-Mediterranean refugia in two species of grass snake. The refuge of a widely distributed western European lineage of the barred grass snake (*Natrix helvetica*) was most likely located in southern France, outside the classical refuges in the southern European peninsulas. One genetic lineage of the common grass snake (*N. natrix*), distributed in Scandinavia, Central Europe and the Balkan Peninsula, had two distinct glacial refuges. We show that one was located in the southern Balkan Peninsula. However, Central Europe and Scandinavia were not colonized from there, but from a second refuge in Central Europe. This refuge was located in between the northern ice sheet and the Alpine glaciers of the last glaciation and most likely in a permafrost region. Another co-distributed genetic lineage of *N. natrix*, now massively hybridizing with the aforementioned lineage, survived the last glaciation in a structured refuge in the southern Balkan Peninsula, according to the idea of ‘refugia-within-refugia’. It reached Central Europe only very recently. This study reports for the first time the glacial survival of a thermophilic egg-laying reptile species in Central Europe.

## Introduction

Climatic oscillations in the Pleistocene induced large-scale range shifts of many animal and plant species all over the world^1^. The extant phylogeographic structure of thermophilic European biota is often explained by their retreat to refugia in southern peninsulas (Iberian, Apennine, Balkan) during the last glacial and postglacial recolonization of northern regions^1–5^. However, there is increasing evidence for extra-Mediterranean refugia^6^, and this is also true for thermophilic species like reptiles^7,8^. Grass snakes (*Natrix astreptophora*, *N. helvetica*, *N. natrix*; formerly lumped together under *N. natrix*^9–11^) are widely distributed across the western Palaearctic^9,12^. The range of the barred grass snake (*N. helvetica*) extends from the Pyrenees to the Rhine region, and it includes the Italian Peninsula, Sicily, Corsica and Sardinia. In the north, the range reaches to Northumberland, close to the Scottish border^9^. The common or eastern grass snake (*N. natrix* sensu stricto) occurs from the Rhine region eastwards to Lake Baikal in Siberia^9,12^. Its northern range extension is controversial, with unambiguous records up to central Sweden and southern Finland and debated records close to the Arctic Circle further north in Fennoscandia^9,13,14^. In any case, these two grass snake species are cold-tolerant compared to many other egg-laying reptile species^9^. This suggests that extra-Mediterranean refugia might have existed, like recently shown for the more thermophilic wall lizard (*Podarcis muralis*). For this species, extra-Mediterranean refugia were inferred for southern France, northern Italy, the eastern Alps and the Central Balkans^8^.

**Figure 1:**
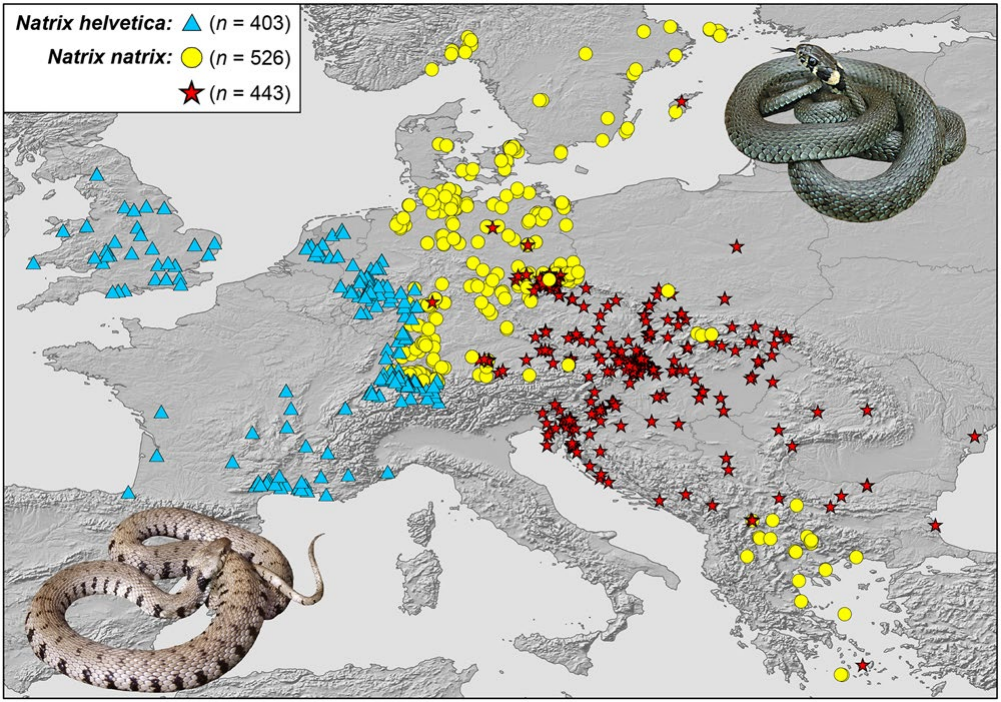
Sampling sites for mitochondrial lineages of the barred grass snake (*Natrix helvetica*, blue triangles) and the common grass snake (*N. natrix*, yellow circles and red stars). Insets: *N. helvetica* (left, Linz am Rhein, Germany; photo: Wolfgang Böhme) and *N. natrix* (right, Moritzburg, Germany; photo: Melita Vamberger). Map was created using arcgis 10.2 (www.esri.com/arcgis) and adobe illustrator CS6 (www.adobe.com/products/illustrator.html).

**Figure 2:**
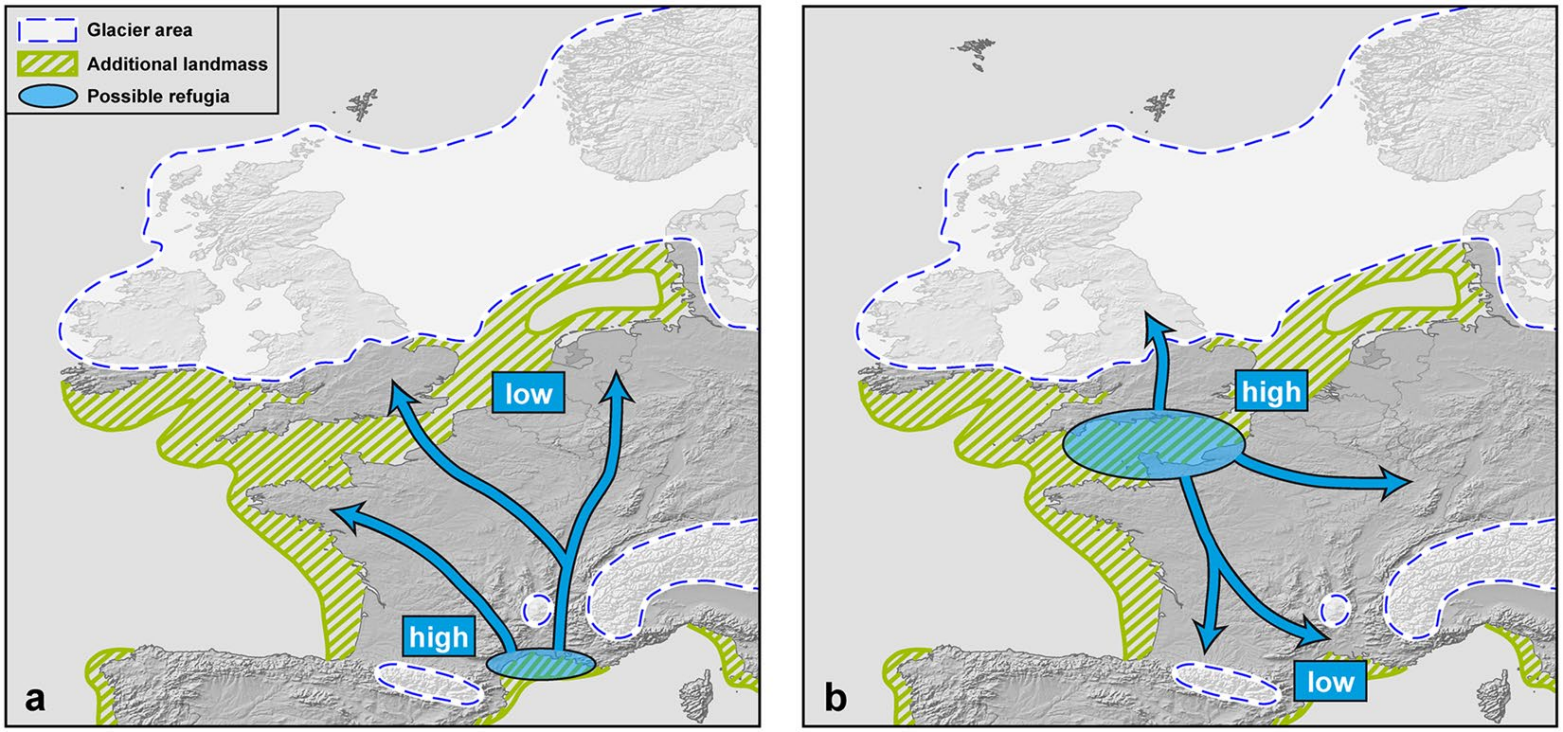
Hypotheses for the location of the glacial refuge and Holocene range expansion of the blue lineage of the barred grass snake (*Natrix helvetica*). Schematic representation of landmass, ice sheet and glacier areas during the Last Glacial Maximum (approx. 20,000 years ago) according to Diercke International Atlas 2010 (ISBN 978-3-14-100790-9). Light blue areas correspond to the possible southern **(a)** or northern refuge **(b)** of the blue lineage of *N. helvetica*. ‘High’ and ‘low’ indicate expected genetic diversities; location of putative refugia is speculative and arbitrary. Map was created using arcgis 10.2 (www.esri.com/arcgis) and adobe illustrator CS6 (www.adobe.com/products/illustrator.html).

A previous study^15^ revealed glacial refugia for grass snakes in each of the southern European peninsulas, Corso-Sardinia, North Africa, Anatolia and the neighbouring Near and Middle East, with multiple microrefugia in continental Italy plus Sicily, the Balkan Peninsula, Anatolia and the Near and Middle East. In continental Italy, one refugium was inferred for the Padan Plain, south of the Alps, which qualifies for an extra-Mediterranean refugium. However, the location of the glacial refugia of some of the most widely distributed mitochondrial lineages of *N. helvetica* and *N. natrix* remain unclear. In *N. helvetica*, the concerned lineage is distributed from the Pyrenees across France, the Benelux countries, Switzerland and westernmost Germany to the Rhine region. Its range also includes Britain up to Northumberland, but not Ireland (Fig. 1: blue lineage). The Apennine Peninsula can be excluded as potential location of its glacial refuge because there are related, but distinct mitochondrial lineages of *N. helvetica* occurring^15^ (Supplementary Fig. S1). Thus, the refuge of the blue lineage could have been either in southern France or further northwest, perhaps in one of the areas emerged from sea during glacial low sea level stands (Fig. 2). If a southern refuge existed, genetic diversity should conform to the classical paradigm of ‘southern richness and northern purity’^1–3^, with high diversity in the south and low diversity in the north (Fig. 2a). If a northern refugium existed (Fig. 2b), a similar, but inversed, pattern is expected, with high genetic diversity in the north and low diversity in the south because ‘the rapid postglacial colonization and founder events inevitably reduce within-lineage genetic diversity’^16^.

In *N. natrix*, the two concerned lineages are distributed from the southern Balkan Peninsula across Central Europe to the Baltic region (Fig. 1: red lineage) or Scandinavia (Fig. 1: yellow lineage), with sympatric records in many regions. However, the red lineage has not been found in the northernmost parts of the species’ range, supporting that the yellow lineage arrived first^14^, either from a southeastern glacial refuge or from a northern extra-Mediterranean refuge. Indeed, we speculated in a pilot study that a refuge north of the Alps could have existed for the yellow lineage^17^ because then no southern records were known. However, using larger and geographically more comprehensive sampling, we found the yellow lineage also widely distributed in the southern Balkans^15^, suggesting rather multiple refuges for the yellow and red lineages and other lineages in the southern Balkan Peninsula. Yet, the southern Balkan records of the yellow lineage are separated by an enigmatic distribution gap from the northern records, whereas the red lineage is continuously distributed from the southern Balkans to Central Europe (Fig. 1)^11,15^.

**Figure 3:**
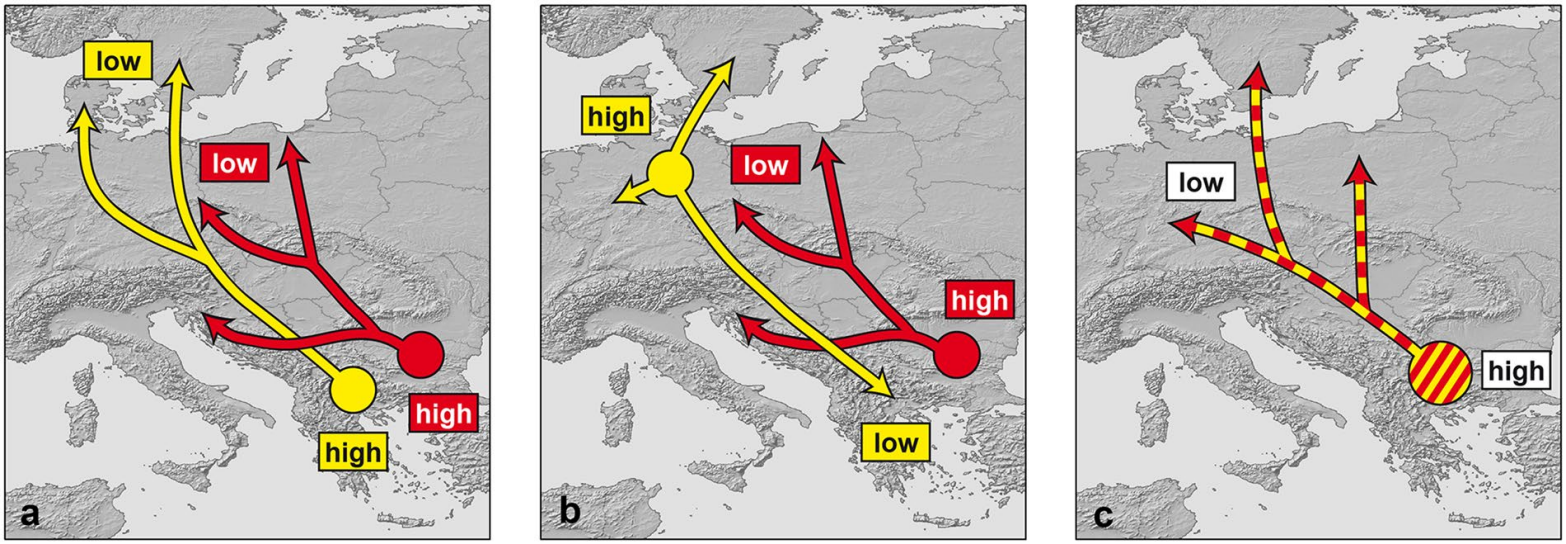
Hypotheses for the location of glacial refuges and Holocene range expansions of the yellow and red lineages of the common grass snake (*Natrix natrix*). **(a)** Range expansions from two distinct southern Balkan refuges (yellow and red circles) leading to secondary admixture in Central Europe and in the Balkan Peninsula, **(b)** antidromic range expansions from a northern (yellow circle) and southern refuge (red circle) leading to secondary admixture in Central Europe and in the Balkan Peninsula, **(c)** range expansion from one Balkan refuge harbouring two distinct mtDNA lineages (ancestral polymorphism). ‘High’ and ‘low’ indicate expected genetic diversities; location of putative refugia is speculative and arbitrary. Maps were created using arcgis 10.2 (www.esri.com/arcgis) and adobe illustrator CS6 (www.adobe.com/products/illustrator.html).

This distribution pattern could result either from range expansions from two distinct refugia in the southern Balkan Peninsula (Fig. 3a) or from antidromic range expansions from a northern refugium of the yellow lineage and from a southern refugium of the red lineage (Fig. 3b). Alternatively, it could be speculated that both mitochondrial lineages existed together in one and the same southern refugium (ancestral polymorphism; Fig. 3c), what could explain their co-occurrence in many regions.

If only southern refugia would have existed (Fig. 3a and c), genetic diversity should be high in the south and low in the north^1–3^. If for the yellow lineage a northern refugium existed (Fig. 3b), the pattern is expected to be inversed, though, with genetic diversity being high in the north and low in the south.

Recent phylogeographic studies^10,11,14,15^ provided a wealth of mitochondrial DNA (mtDNA) and microsatellite data for grass snakes. However, nuclear gene pools turned out to be highly admixed, especially in the south of the range of *N. natrix*^11^, with several genetic lineages involved (Supplementary Fig. S1). Thus, microsatellite data are not feasible for unravelling the location of glacial refugia because high genetic diversity could be the result from admixture and not ‘southern richness’ of an individual genetic lineage. In contrast, maternally inherited mtDNA is not affected by admixture so that data for nearly 1,400 grass snakes are expected to be informative for inferring the location of former glacial refugia, even though nuclear DNA of the same grass snakes may be impacted by hybridization.

## Materials and Methods

### Sampling and used marker system

Geographically broad sampling of 1,372 grass snakes covered the distribution ranges of the blue lineage of *Natrix helvetica* and the yellow and red lineages of *N. natrix*^11,15^. In our previous studies^11,15^, we used 13 polymorphic microsatellite loci and two mitochondrial DNA blocks, the cytochrome *b* gene and the partial ND4 gene plus adjacent DNA coding for tRNAs = ND4 + tRNAs (866 bp). Here we focus on the latter mitochondrial marker because more data are available and differentiation patterns were found to be the same^11,15^. Microsatellite data could not be used for examining the location of glacial refuges because in the south massive admixture of distinct genetic lineages^11^ causes high diversity indices that have nothing to do with the original diversity in former refuges. The same is true for the Rhine region, where a narrow hybrid belt of the two grass snake species results in high nuclear genomic diversity^11^.

Among the used mtDNA sequences of grass snakes, 403 corresponded to the blue lineage of *N. helvetica*^11,15^, 526 to the yellow lineage and 443 to the red lineage of *N. natrix*. An additional sequence from a *N. helvetica* from northwestern Italy, generated according to laboratory procedures described before^15^, was added to our previously published data set^11^. Sequences of obviously non-native grass snakes from the study regions^11^ were excluded from calculations. All samples studied are listed in Supplementary Table S1.

The new sample (from a roadkill) was obtained and methods were carried out according to relevant guidelines, regulations and best ethical and experimental practice of the Senckenberg Nature Research Society.

### Haplotype network analyses and PCAs

Using popart (www.popart.otago.ac.nz) and the implemented parsimony network algorithm of tcs^18^, a network was built for the 1,372 mtDNA sequences for displaying relationships of haplotypes. In addition, Principal Component Analyses (PCAs) were calculated using the same data set and the R package adegenet^19^.

### Genetic diversity indices and demographic analyses of mtDNA

**Figure 4:**
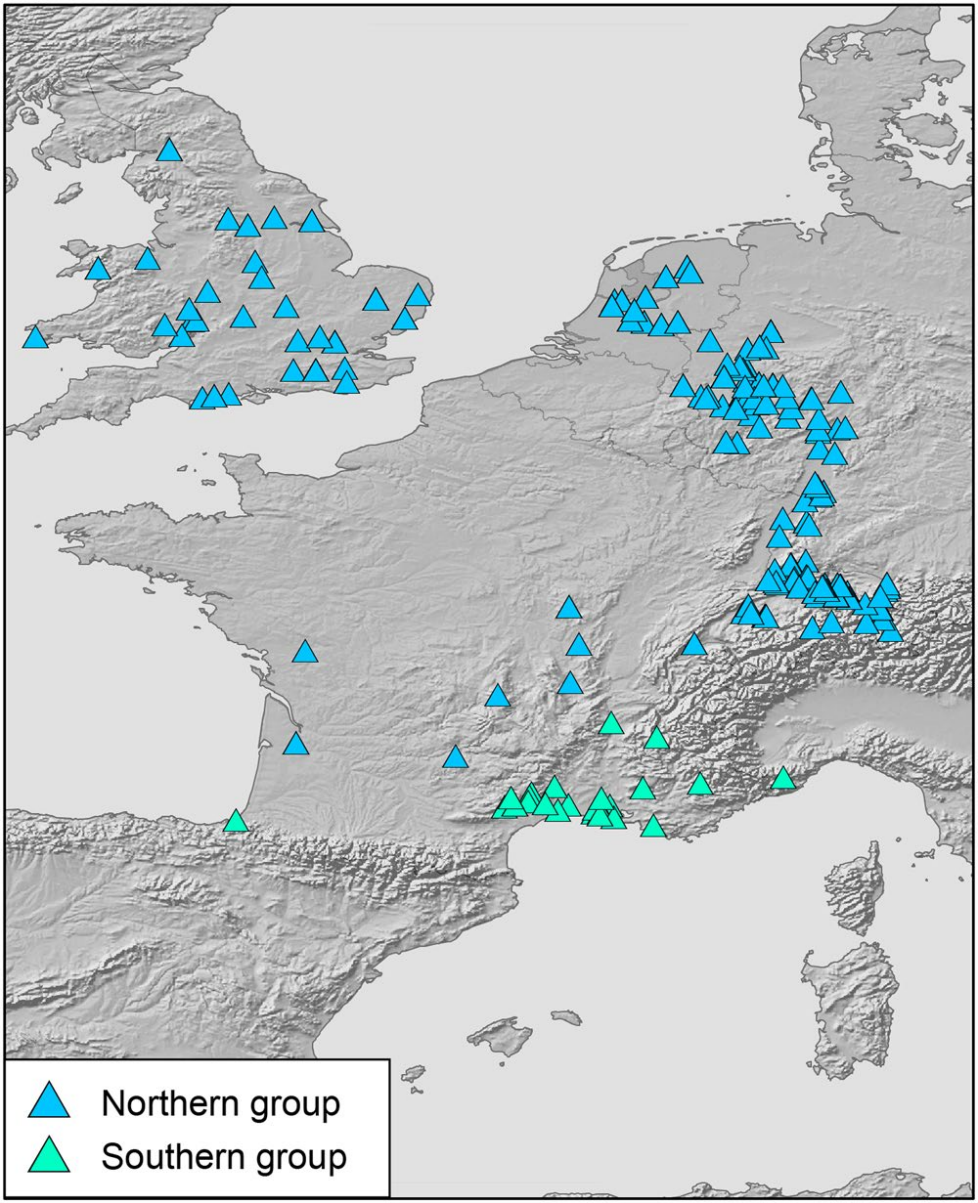
Northern and southern groups for samples of *Natrix helvetica* (*n* = 403). Map was created using arcgis 10.2 (www.esri.com/arcgis) and adobe illustrator CS6 (www.adobe.com/products/illustrator.html).

**Figure 5:**
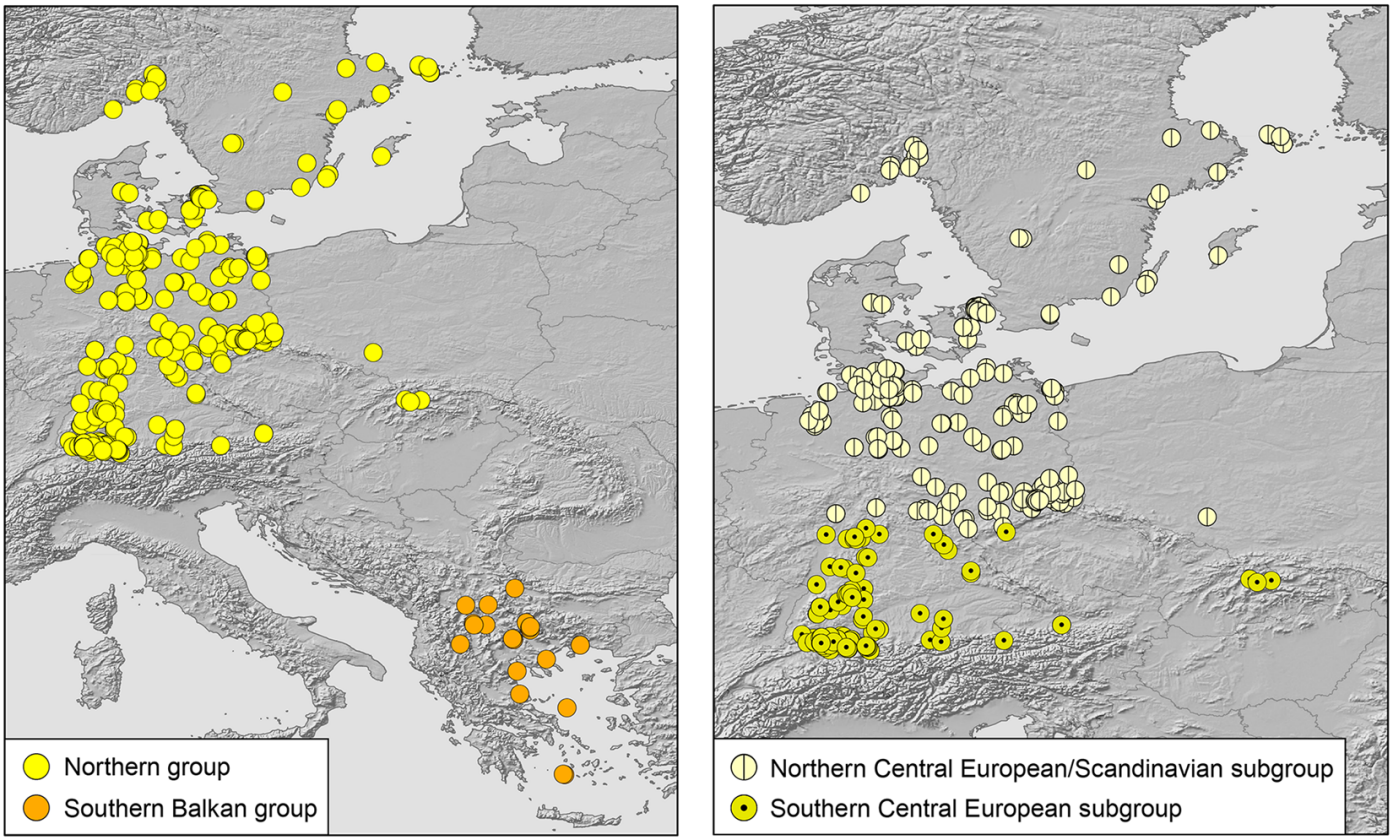
Northern and southern groups of the yellow lineage (*n* = 499) of *Natrix natrix* (left) and subdivision of the northern group (right). Maps were created using arcgis 10.2 (www.esri.com/arcgis) and adobe illustrator CS6 (www.adobe.com/products/illustrator.html).

**Figure 6:**
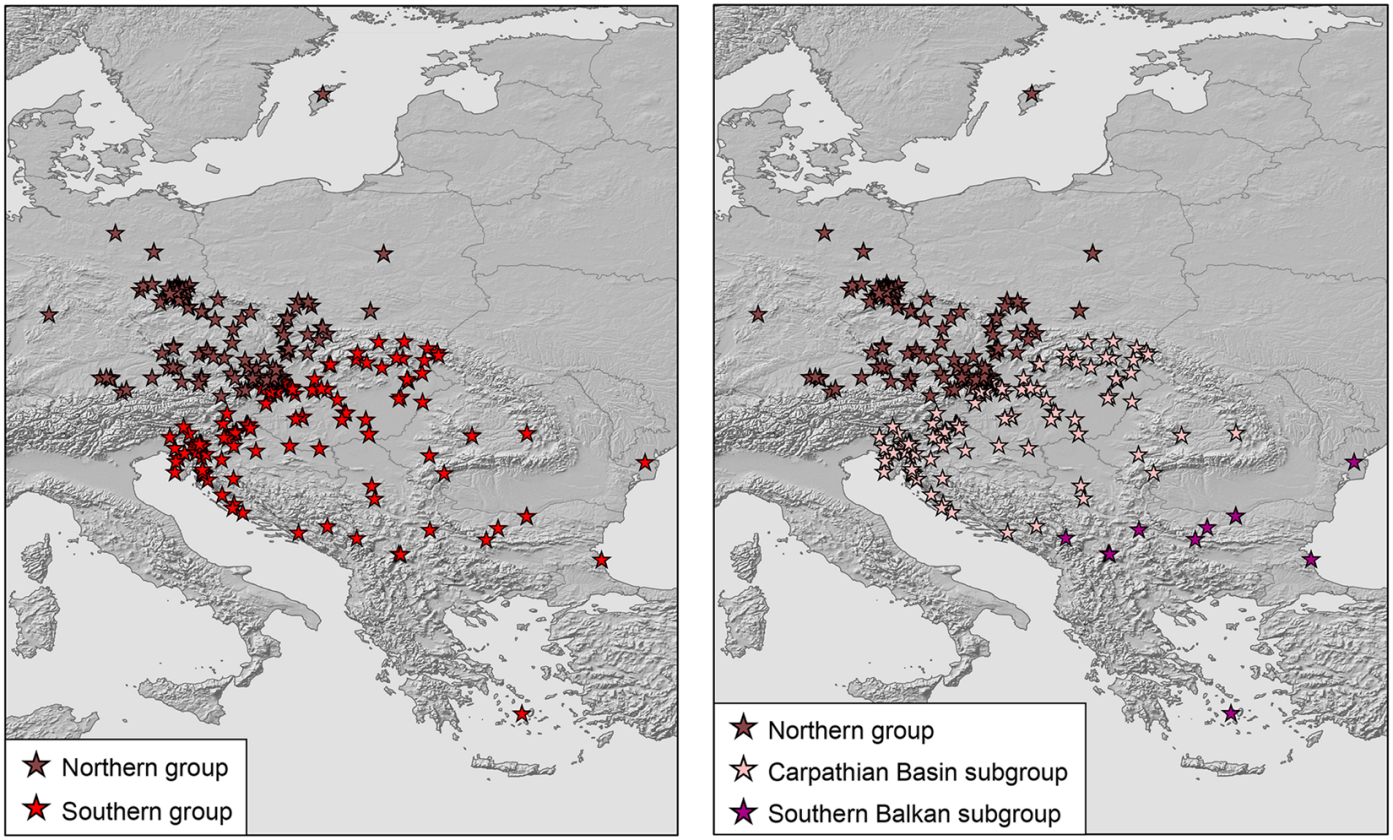
Northern and southern groups of the red lineage (*n* = 443) of *Natrix natrix* (left) and subdivision of the southern group (right). Maps were created using arcgis 10.2 (www.esri.com/arcgis) and adobe illustrator CS6 (www.adobe.com/products/illustrator.html).

Genetic diversity in glacial refugia should be much higher than in recently colonized regions^1,2,16^. To examine this expectation, samples were arbitrarily assigned to northern and southern groups. For the blue lineage of *N. helvetica*, the divide was the Massif Central in southeastern France (Fig. 4). For the yellow and red lineages of *N. natrix*, the Carpathians served as primary divide (Figs 5 and 6, left). Because the yellow lineage has a wide distribution gap in the northern Balkan Peninsula^11,15^, no data were available for the Carpathian Basin. Thus, the southern group of the yellow lineage corresponds only to the southern Balkan. For fine scale analysis of genetic diversities, the data sets were further subdivided (Figs 5 and 6, right). For the northern yellow group, genetic diversity was examined for two subgroups north and south of a line running approximately from the Ore Mountains to the Hunsrück. The southern red group was approximately subdivided along the Balkan Mountains.

To assess genetic diversity of each group, the following diversity indices were calculated using dnasp 5.10.01^20^: number of segregating sites *S*, nucleotide diversity *π*, number of haplotypes *h*, and haplotype diversity *Hd*. The number of private haplotypes h_P_ was determined in arlequin 3.5.1.2^21^. dnasp was also used to examine for signals for population size changes by generating pairwise mismatch distributions. Unimodal mismatch distributions are expected when range or demographic expansions lead to an excess of recent, and thus rare, mutations. In contrast, multimodal mismatch distributions indicate stationary population sizes^22–25^. Moreover, a mismatch distribution test was run in arlequin and parameters of sudden demographic expansion (τ, θ_0_ and θ_1_) were calculated. To verify the validity of the expansion model, a parametric bootstrap approach was used, comparing the fit of the observed and 100 simulated mismatch distributions. The fit to the expected mismatch distribution was quantified by the sum of square deviations (SSD). SSD values are non-significant when the data do not deviate from the expectation of population expansion. Raggedness indices (rg)^26,27^ were computed quantifying the smoothness of the observed mismatch distribution. Non-significant results indicate an expanding population^27^. Finally, changes in population size were examined by calculating Tajima’s *D* and Fu’s *F*_S_ values in dnasp. An increasing population size (or purifying selection) is indicated by significantly negative *D* and *F*_S_ values, whereas positive values suggest a recent bottleneck^28,29^.

Sample sizes differed considerably among populations. In order to eliminate the positive bias of sample size on inferred diversity, a rarefaction procedure was employed. For each sample larger than the smallest one with size *N*, a subsample of *N* individuals was drawn randomly (without replacement) and diversity indices were calculated. The procedure was repeated five times for each sample, and then the mean subsample diversity for each sample was calculated.

**Figure 7:**
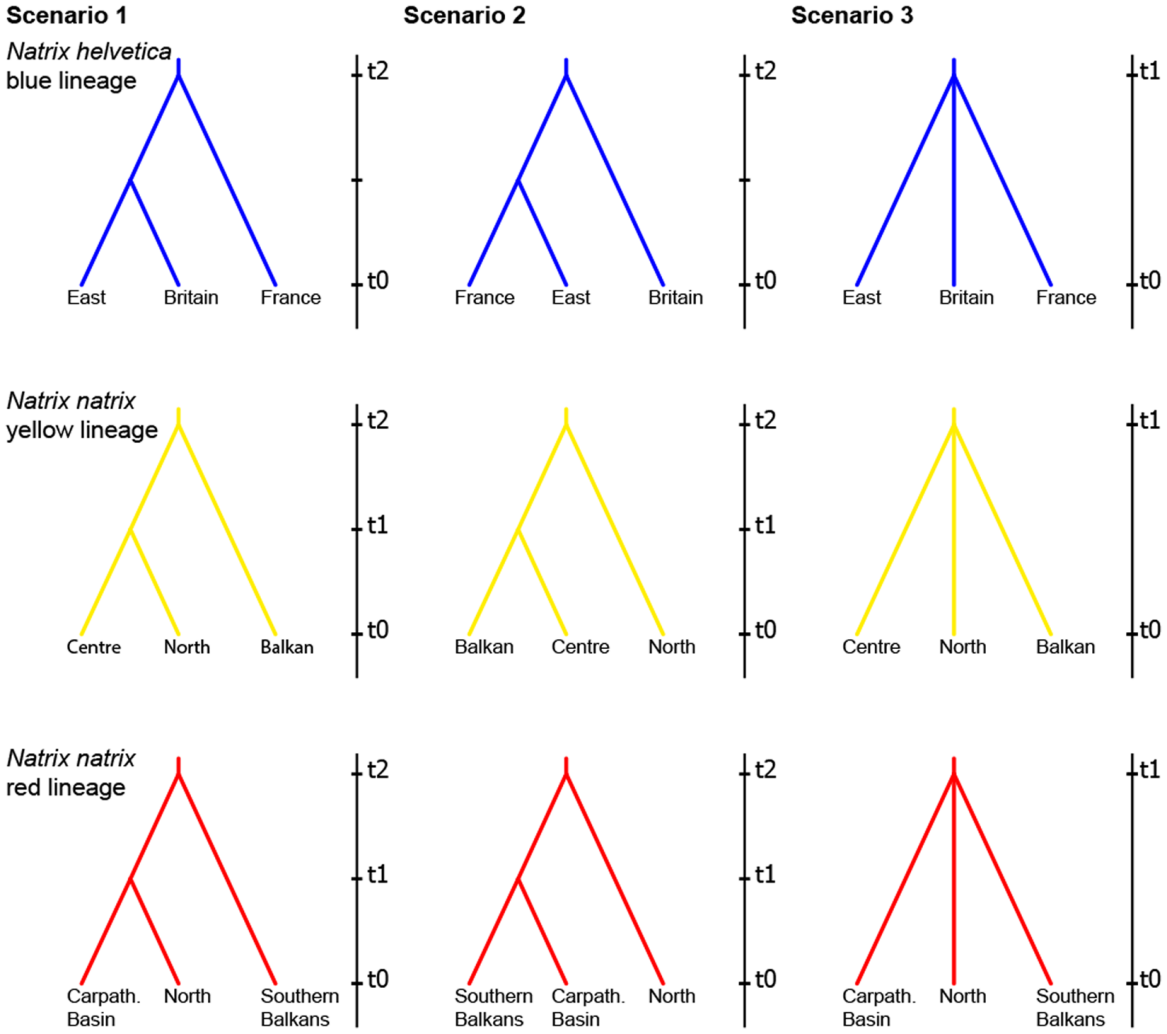
Phylogeographic hypotheses for genetic lineages of *Natrix helvetica* and *N. natrix* explicitly tested using diyabc. Scenarios 1 and 2 correspond to gradual diversification from an ancient population, while scenario 3 corresponds to sudden expansion.

For comparing specific hypotheses for the demographic history of the three studied lineages (blue, yellow and red), we utilized the Approximate Bayesian Computation approach implemented in diyabc 2.0.3^30^. This software allows for rapid testing of different scenarios by calculating summary statistics rather than exact likelihoods^31^. For each lineage, three alternative scenarios were examined that represented either gradual diversification from refuge areas or sudden expansion (Fig. 7). Three groups were defined for each lineage and a rarefaction procedure (as described above) was applied to obtain even sample sizes. For the blue lineage of *N. helvetica*, the groups corresponded to samples from Britain, France, and the Rhine region (East). For *N. natrix*, the yellow lineage was divided into the groups North (Scandinavia), Centre (Central Europe), and Balkan. The red lineage was divided into the groups North, Carpathian Basin, and southern Balkans. Priors were set to follow uniform distributions ranging from 10 to 10,000 for effective population sizes (*N*_e_) and from 10 to 10,000 for diversification times (t1 and t2, in generations). For each lineage 300,000 simulations were run, after which summary statistics were drawn for: (i) number of haplotypes, (ii) number of segregating sites, (iii) mean of pairwise differences, (iv) private segregating sites, (v) mean of pairwise differences (W), (vi) mean of pairwise differences (B), and (vii) *F*_ST_. According to previous studies^32,33^, the priors for the mutation rate were set to a minimum of 10^−8^ and a maximum of 10^−6^. All other settings were left as suggested in the diyabc 2.0 handbook. Posterior probabilities for scenarios were calculated by logistic regression considering the 3,000 simulated datasets that were closest to the observed values. To convert the obtained estimates of divergence times from generations to years, a generation time of 10 years was used, based on life history data (sexual maturity, maximum age)^9^.

### Data availability

All data analysed during this study are included in this published article and its Supplementary Information files. DNA sequences have been uploaded to the European Nucleotide Archive (ENA) and are available under the accession numbers listed in Supplementary Table S1.

## Results

### Haplotype networks and PCAs

**Figure 8:**
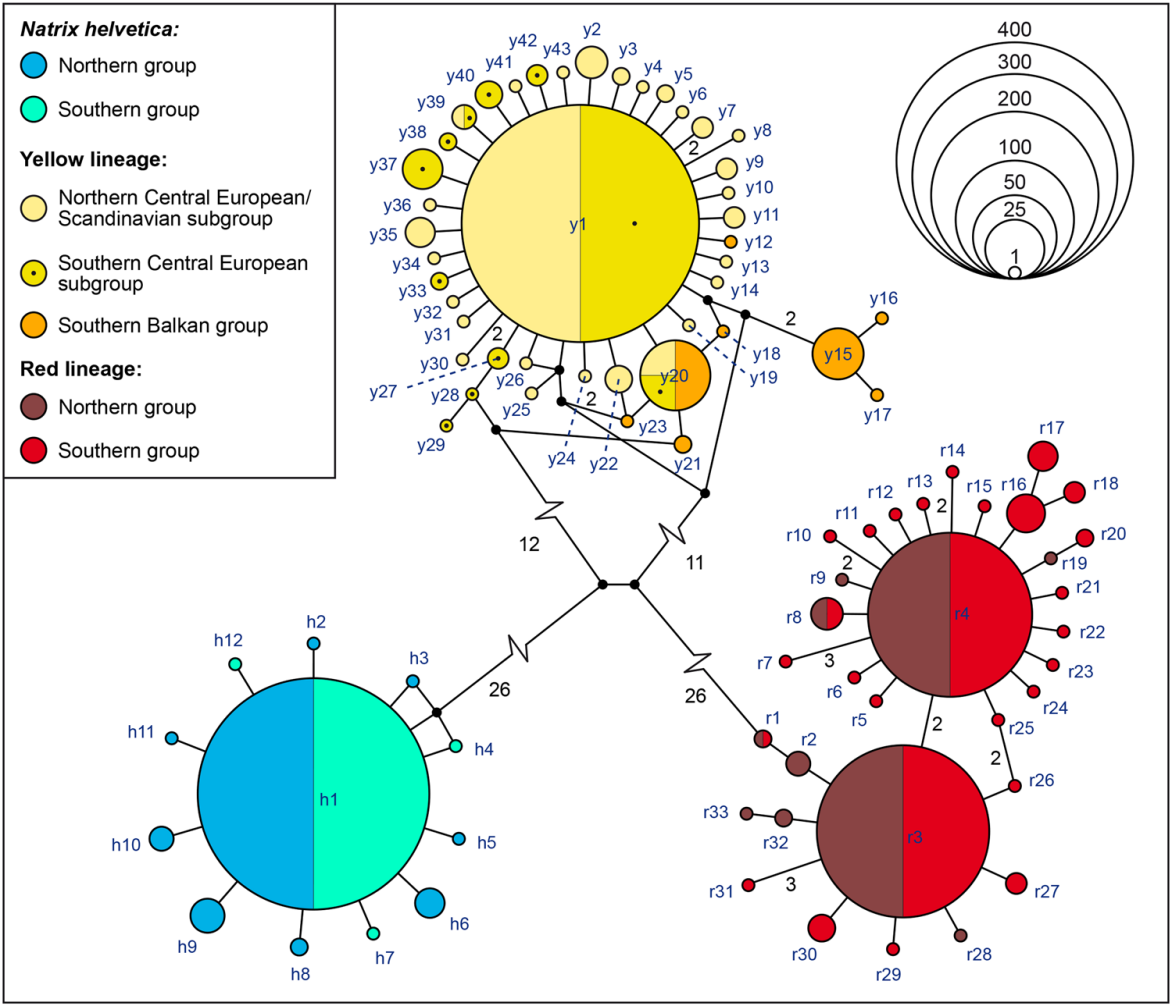
Parsimony networks of 1,372 ND4+tRNA sequences of barred and common grass snakes. Symbol sizes reflect haplotype frequencies. Small black circles are missing node haplotypes; each line connecting two haplotypes corresponds to one mutation step, if not otherwise indicated by numbers. Haplotype colours represent mitochondrial lineages. Lighter or darker nuances show the occurrence of haplotypes in the northern or southern distribution area according to Figs 4, 5 (left) and 6 (left). Two-coloured haplotypes occurred in the respective northern and southern groups; colours do not indicate percentages of occurrence.

The haplotypes of each grass snake lineage corresponded to a highly distinct cluster in parsimony network analyses (Fig. 8; for geographic distribution of individual haplotypes, see Supplementary Figs S2–S5). Haplotypes of *Natrix helvetica* (blue lineage) were separated by a minimum of 40 mutation steps from the haplotype cluster of the yellow lineage of *N. natrix*, and by a minimum of 54 steps from the haplotypes of the red lineage of *N. natrix*. Between the two clusters of *N. natrix*, a minimum of 40 mutations occurred. Haplotypes of all lineages had star-like genealogies with a common internal haplotype surrounded by rare haplotypes differing only in one or a few mutations from the central haplotype. However, only for *N. helvetica* (blue lineages) this topology was unambiguous, with the internal haplotype h1 and eleven rare haplotypes (h2–h11) differing in only one mutation step from h1. There was no obvious pattern of a geographically correlated distribution of any haplotypes.

The haplotype cluster of the yellow lineage of *N. natrix* consisted also of a common central haplotype (y1) surrounded by many rare haplotypes that differed in one or two mutations from y1. Yet, there were some network reticulations, and the more distinct haplotypes y15-y17 differed by four to five mutations from y1. Notably, these haplotypes y15-y17 and the rare haplotypes y12, y21 and y23 occurred only in the southern Balkan region, whereas the vast majority of other haplotypes was found only north of the Carpathians. There was only one shared haplotype (y20) between the two geographical groups. However, some unique southern haplotypes (y12, y18, y21, y23) differed in only one or two mutations from the most common central northern haplotype y1.

The haplotype cluster of the red lineage of *N. natrix* corresponded not to one, but to two star-like subclusters, with tip haplotypes differing in up to three mutations from the two frequent central haplotypes r3 and r4. The latter two haplotypes differed by two mutation steps from one another. There was no clear pattern in geographical distribution of haplotypes of the red lineage, with haplotypes of both subclusters occurring north and south of the Carpathians.

In accordance with the network analyses, the PCAs revealed three highly distinct clusters corresponding to the blue, yellow and red lineages (Supplementary Fig. S6). When PCAs were calculated for the sequences of each lineage alone, only for the yellow lineage the northern and southern groups were clearly differentiated, with largely non-overlapping 95% confidential intervals. The confidential intervals of the northern and southern groups of the blue and red lineages were massively overlapping, reflecting that these lineages comprise less differentiated haplotypes or haplotype clusters (Supplementary Fig. S7).

### Genetic diversity indices and demographic analyses of mtDNA

Diversity indices increased with sample size, suggestive of a positive sampling bias. Therefore, we applied our rarefaction procedure to obtain balanced sample sizes (Table 1, Supplementary Tables S2–S5). However, with respect to mismatch distributions, no differences were observed for total and rarefied samples (Supplementary Tables S6–S11).

**Table 1:** Genetic diversities for ND4+tRNA of three lineages of grass snakes. Large samples were reduced using a rarefaction procedure as described under ‘Genetic diversity indices and demographic analyses of mtDNA’. For groups and subgroups, see Figs 4–6.

Group	*n*	*S*	*h*	h_P_	*Hd*	π (*10^−3^)
**Blue lineage of** ***Natrix helvetica*** (Fig. 4)						
Northern group (total)	370	8	9	8	0.125	0.15
Northern group (ø rarefied)	33	2	3	2	0.107	0.13
Southern group (total)	33	3	4	3	0.176	0.21
**Yellow lineage of** ***Natrix natrix*** (Fig. 5)						
Carpathians as divide:						
Northern group (total)	499	37	36	35	0.388	5.30
Northern group (ø rarefied)	27	5	5	5	0.385	0.49
Southern Balkan group (total)	27	11	8	7	0.638	3.64
Northern group subdivided:						
Northern Central European/Scandinavian subgroup (total)	334	29	28	25	0.414	0.55
Northern Central European/Scandinavian subgroup (ø rarefied)	165	16	17	14	0.399	0.52
Southern Central European subgroup (total)	165	10	11	8	0.326	0.45
**Red lineage of** ***Natrix natrix*** (Fig. 6)						
Carpathians as divide:						
Northern group (total)	202	10	10	6	0.498	1.16
Southern group (total)	241	34	27	23	0.672	1.71
Southern group subdivided:						
Northern group (ø rarefied)	11	2	2	0	0.345	0.81
Carpathian Basin subgroup (total)	230	32	26	21	0.671	1.69
Carpathian Basin subgroup (ø rarefied)	11	5	4	2	0.680	1.75
Southern Balkan subgroup (total)	11	6	4	1	0.691	1.93

*n* = sample size; *S* = number of segregating sites; *h* = number of haplotypes; h_P_ = number of private haplotypes, *Hd* = Haplotype diversity, π = nucleotide diversity.

**Figure 9:**
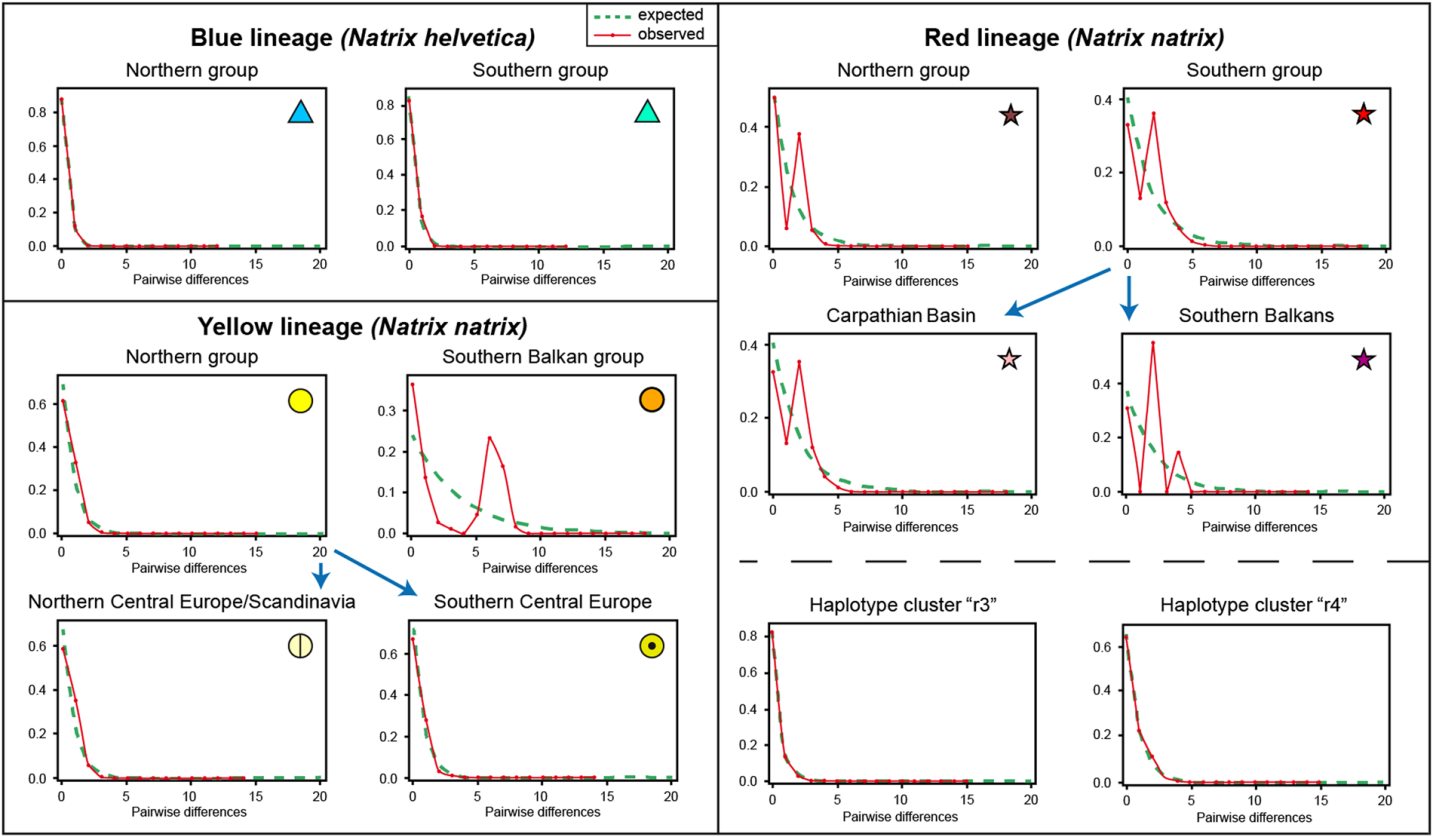
Pairwise mismatch distributions using all samples of the respective groups. Rarefied sampling resulted consistently in the same patterns. Broken green lines are expected frequencies of pairwise differences under demographic expansion. Solid red lines and circles, observed values. Symbols correspond to Figs 4–6.

For the blue lineage of *Natrix helvetica*, diversity in the south was higher than in the north when the much larger northern sample was rarefied (Table 1). Mismatch distributions were unimodal (Fig. 9) and Tajima’s *D* and Fu’s *F*_S_ values were significantly negative for both groups considering all data, suggestive of population expansion. This is supported by non-significant values of the sum of square deviations (SSD) and the raggedness index (rg) indicating that the data do not deviate from the expectation of population expansion (Supplementary Table S6).

For the yellow lineage of *N. natrix*, diversities of the southern Balkan group were higher than for the northern group using a rarefied northern sample (Table 1). For the northern group, there was a signal for recent expansion (unimodal mismatch distribution, Fig. 9), which is in line with significantly negative Tajima’s *D* and Fu’s *F*_S_ values and non-significant SSD and rg values (Supplementary Table S6). However, the southern Balkan group was characterized by a multimodal mismatch distribution (Fig. 9), with non-significant positive Tajima’s *D* and Fu’s *F*_S_ values, arguing for a stationary, non-expanding population. Contrary to expectation, SSD and rg values were also non-significant (Supplementary Table S6).

To examine genetic diversity on both sides of the distribution gap in the northern Balkans (Fig. 5 left), the northern group was subdivided along a line running from the Ore Mountains to the Hunsrück (Fig. 5, right). Genetic diversity in the northern Central European and Scandinavian subgroup was higher than in the southern subgroup (Table 1). For both subgroups, mismatch graphs were unimodal and Tajima’s *D* and Fu’s *F*_S_ values were significantly negative and SSD and rg values were not significant, indicating population expansion (Fig. 9; Supplementary Table S6).

Sample sizes of the red lineage north and south of the Carpathians (Fig. 6, left) were nearly even, so that no rarefication was required. Genetic diversity was distinctly higher south of the Carpathians (Table 1). Mismatch distributions were multimodal for the northern and southern groups (Fig. 9), suggestive of stationary population sizes. However, this was not supported by mostly significant negative Tajima’s *D* and Fu’s *F*_S_ values and non-significant SSD and rg values, suggesting rather expansion (Supplementary Table S6). When the southern group was subdivided into the two subgroups Carpathian Basin and southern Balkan Peninsula (Fig. 6, right), highly uneven sample sizes were obtained because only 11 samples were available for the southern Balkan Peninsula. Using the rarefication approach described above to achieve similar sample sizes, diversities in both southern subgroups were similar and higher than in the northern group (Table 1). Mismatch distributions were multimodal for both southern subgroups (Fig. 9). However, Tajima’s *D* and Fu’s *F*_S_ values were significantly negative for the Carpathian Basin, complemented by non-significant SSD and rg values (Supplementary Table S6). The Tajima’s *D* value for the southern Balkan subgroup was negative, the Fu’s *F*_S_ value was positive, but both were not significant. Significant SSD and rg values support stationary population sizes indicated by the multimodal mismatch distribution graph for the southern Balkans (Supplementary Table S6).

The haplotypes of the red lineage corresponded to two distinct star-like clusters (Fig. 8), which were also analysed separately. Genetic diversity of the cluster with the central haplotype “r4” was much higher than in the cluster having the central haplotype “r3” (Supplementary Table S12). For both clusters, mismatch distributions were unimodal (Fig. 9), SSD and rg values were not significant. Tajima’s *D* and Fu’s *F*_S_ values were for both clusters significantly negative (Supplementary Table S13), suggesting expansion.

The models preferred by our ABC analyses (Fig. 7; Table 2; Supplementary Figs S8 and S9) supported the results of the other demographic analyses. If the northern populations are derived from Holocene range expansions from a southern refuge, either scenario 1 (stepwise range expansion from south to north) or scenario 3 (sudden expansion) were expected. The stepwise range expansion of scenario 1 could also correspond, however, to two distinct glacial refugia if the time elapsed between the branching events would conflict with Holocene expansion. Conversely, scenario 2 would indicate a stepwise southward range expansion from the north. For the blue lineage of *N. helvetica* and the red lineage of *N. natrix*, scenario 3 was best supported (Table 2). The sudden expansion for the blue lineage was dated to 12,000 years before present (BP), while the expansion was dated only to approximately 3,000 BP for the red lineage. For the yellow lineage of *N. natrix* scenario 1, representing a stepwise range expansion from the Balkan, was supported. The divergence of the Balkan group and the common ancestor of the central and northern groups was dated to approximately 39,000 BP, whereas the divergence of the Central and northern European groups was dated to 7,700 BP (Table 2).

**Table 2:** Posterior probabilities and credibility intervals [CI] for scenarios tested using diyabc (Fig. 7). Favoured scenarios in **bold** and with asterisks; divergence times estimates in years before present (median and 95% confidence interval) concern the favoured scenarios.

	Scenario choice (logistic regression)			Divergence times	
	Scenario 1	Scenario 2	Scenario 3	t1	t2
*Natrix helvetica* (blue lineage)	0.25 [0.21–0.38]	0.28 [0.13–0.45]	0.48* **[0.46–0.50]**	12,000 [1,260–72,400]	Not included in selected scenario
*Natrix natrix* (yellow lineage)	0.88* **[0.87–0.90]**	0.03 [0.02–0.04]	0.08 [0.07–0.10]	7,700 [2,170–28,700]	39,300 [9,330–91,000]
*Natrix natrix* (red lineage)	0.10 [0.09–0.12]	0.21 [0.19–0.24]	0.69* **[0.66–0.71]**	3,290 [950–14,800]	Not included in selected scenario

## Discussion

**Figure 10:**
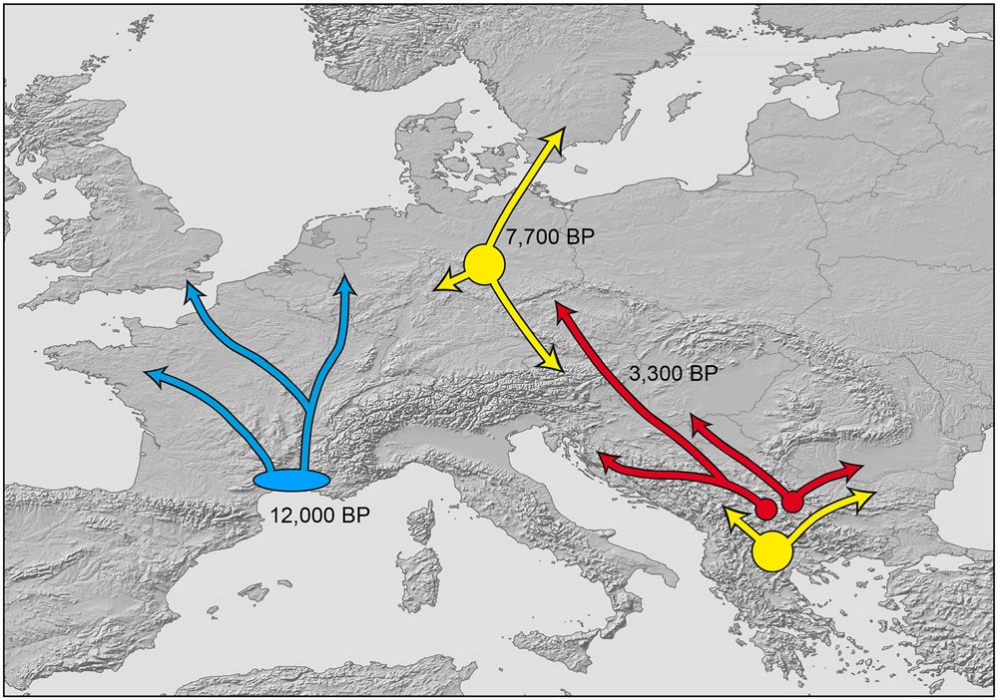
Putative glacial refuges and schematic range expansions of genetic lineages of *Natrix helvetica* (blue lineage) and *N. natrix* (yellow and red lineages). Expansion times from diyabc indicated. Exact location of refugia is arbitrary. Map was created using arcgis 10.2 (www.esri.com/arcgis) and adobe illustrator CS6 (www.adobe.com/products/illustrator.html).

Our analyses of genetic diversities of mtDNA suggest for the blue lineage of the barred grass snake (*Natrix helvetica*) and the red lineage of the common grass snake (*N. natrix*) glacial refugia in the south, close to the Mediterranean Sea or inland of the southern Balkan Peninsula. This is indicated by higher genetic diversities in the former refuges and supported by our diyabc analyses. Typically, such refugia are thought to be located in the southern peninsulas^1,4,5,34–37^. This was not the case for the blue lineage of *N. helvetica*, because the two peninsulas adjacent to its range, the Iberian and Italian peninsulas, are occupied by genetically distinct grass snakes (*N. astreptophora* in the Iberian Peninsula, several distinct lineages of *N. helvetica* in the Italian Peninsula^15^ (Supplementary Fig. S1). Our new sample from northwestern Italy constitutes the southeastern most record of *N. helvetica*. Thus, our results are in line with the previous suggestion^15^ that the refuge of the blue lineage of *N. helvetica* was in southern France (Fig. 10). This region has also been inferred as a possible glacial refuge for other reptile species (*Podarcis muralis*^8^, *Zootoca vivipara*^38^, *Vipera aspis*^39^, *V. berus*^40^). From there, the blue lineage seems to have rapidly expanded its range approximately 12,000 BP (Table 2). This date coincides with a rapid warming event evinced from ice core and climate records from northern Europe that has triggered also range shifts in other species^41–43^ and explains the colonization of Britain by *N. helvetica* via what is now the English Channel.

For the red lineage of *N. natrix*, the higher southern genetic diversity indicates that its glacial refuge was located in the southern Balkan Peninsula, conforming to one of the classical refuges^4,5,34,44–46^. However, the structured, bipartite haplotype network and the multimodal mismatch distributions of the red lineage of *N. natrix* support a different demographic history compared to the blue lineage of *N. helvetica*. The network for the blue lineage is perfectly star-like, as typical for demographic expansions, coupled or not with range expansions. Such networks are characterized by a presumably ancestral common central haplotype. Expansion is also supported by an excess of rare mutations corresponding to rare tip haplotypes, and unimodal mismatch distributions. In contrast, intricate network topologies with multiple internal haplotypes and multimodal mismatch distributions as in the red lineage argue normally for stationary and geographically structured populations^22,23,25,47^. However, for the red lineage, some demographic analyses were conflicting. When the samples corresponding to two star-like haplotype clusters were examined separately for the red lineage, signal for expansion was obtained. This suggests that the refuge of the red lineage was structured into two microrefugia (or ‘refugia within refugia’^48^). These putative microrefuges, and the occurrence of the yellow and other mitochondrial lineages in the southern Balkan Peninsula, are in line with the idea of multiple glacial refuges of grass snakes^15^ and other reptiles^8,49–51^ there. The microrefuges of the red lineage must have been in very close proximity, so that haplotypes of each microrefuge came rapidly into contact, spread together and are now co-distributed across the entire range (Fig. 10). Also our diyabc analyses support a range expansion from the south, which is, however, very recent with only approximately 3,300 BP.

Haplotypes of the red lineage are occurring together with haplotypes of the yellow lineage in many parts of their distribution ranges. Theoretically, such a pattern could also originate from ancestral polymorphism, i.e. from the survival of the two mitochondrial lineages in one and the same refugial population. However, based on our previously published analyses of 13 microsatellite loci^11^, this hypothesis can be firmly rejected, because then only one nuclear gene pool should correspond to grass snakes harbouring haplotypes of the yellow and red mtDNA lineages. This is not the case. The yellow and the red mtDNA lineages clearly match with two distinct nuclear clusters (Supplementary Fig. S10), which is why for both distinct refuges have to be inferred.

Consequently, the question remains whether the yellow lineage of *N. natrix* had another refuge in the southern Balkan Peninsula, in close proximity of those of the red lineage, or whether the yellow lineage might have survived the last glaciation somewhere in a northern extra-Mediterranean refuge^17^ (Fig. 3). At first glance our data support a southern refugium because genetic diversity in the south is much higher (Table 1). However, our PCA analyses indicate that the northern and southern groups of the yellow lineage are genetically distinct, a sharp contrast to the blue and red lineages (Supplementary Fig. S7). Moreover, demographic analyses suggest no Holocene expansion for the southern group, in contrast to the northern one. There is only one shared haplotype between the northern and the southern groups (Fig. 8), and the central haplotype of the northern group has not been found in the southern Balkans. If the northern grass snakes spread from a southern Balkan refuge, it should be expected that more haplotypes of the southern group occur also in the north and that a southern haplotype is ancestral to northern tip haplotypes. Also when the southern group were derived from the northern one, we should expect a similar, but inversed pattern (Fig. 3b), with high diversity in the north and low diversity in the south. Then, the central haplotype should occur in the northern group. This scenario of an exclusively northern refuge for the yellow lineage is rejected by our diyabc calculations (Table 2) and not supported by any demographic analyses.

In the face of the wide distribution gap in the northern Balkans, yet another possibility comes into mind, namely two glacial refugia, one north of the Alps and one in the southern Balkan Peninsula. It seems plausible that the last glaciation interrupted an originally continuous population of the yellow lineage, with survivors in two distinct refuges, one in the north and one in the south. This hypothesis is supported by the high genetic diversity indices of northern Central European and Scandinavian grass snakes compared southern Central European ones (Table 1) and by our diyabc analyses (Table 2). The diyabc analyses suggest that the Balkan group separated from the more northerly populations of the yellow lineage approximately 39,000 years ago. This predates the Last Glacial Maximum (LGM) and coincides with the time when the ice sheets first reached their local last glacial maxima and the sea level dropped first to glacial lowstand^52^, suggesting that the range interruption was caused by corollary environmental changes. The divergence of the Scandinavian and Central European populations was dated to only 7,700 BP, arguing for a colonization of Scandinavia from the Central European source populations during the Holocene climatic optimum.

The absence of the red mitochondrial lineage in northern Europe and its rareness in northern Central Europe (Fig. 1) is in agreement with late invasion^14^ and our ABC modelling, which suggests that the red lineage expanded its range into Central Europe only 3,300 BP (Table 2). Late invasion is also supported by the largely pure nuclear gene pool of grass snakes of the yellow lineage in northern Germany and southern Scandinavia, without admixture with the red nuclear cluster^11^. This pattern perfectly matches with the idea of a northern refuge of the yellow lineage because grass snakes of the yellow lineage had to cover much lower distances to reach Scandinavia than grass snakes of the red lineage coming from the southern Balkans.

Also for other taxa, there is growing evidence for extra-Mediterranean glacial refugia, in particular in the Alpine region, the Carpathian and the Caucasian regions^6,7,53–71^. However, our data (Tables 1 and 2) suggest an even more northerly location for the refuge of the yellow lineage of *N. natrix*, namely within Central Europe, i.e. in between the northern ice sheet and the Alpine glaciers of the last glaciation (Fig. 10). In this region, refuges are expected mainly, but not exclusively, for artic or alpine species^6,53–55^, but not necessarily for an egg-laying thermophilic reptile. Such northern refugia are controversially debated^55,72–74^, especially with respect to their location in permafrost regions. For instance, the glacial survival of the adder (*Vipera berus*), a species widely co-distributed with grass snakes, has been doubted for permafrost regions^40^. Yet, even today, grass snakes occur in regions with discontinuous and continuous permafrost, for example near Lake Baikal in Siberia^9,12,75,76^, and the same is true for the adder^12,76^. Based on fossil and genetic evidence, Bhagwat & Willis^72^ examined glacial refuges of more than 50 European vertebrate species and concluded that there is a positive correlation between present-day distribution (northern range limits beyond 60°N) and persistence in northern glacial refugia. Species with northern range limits beyond 60°N are likely to have survived in northern refuges, and *N. natrix* qualifies for this category. Even if the northernmost questionable records^9,13,14^ are disregarded, there are unambiguous records of grass snakes beyond 60°N (for instance in Finland at 61.9°N^14^).

With respect to refugia in permafrost regions it has to be kept in mind that this does not necessarily imply life on permanently frozen ground. The surface of permafrost soils thaws during the warm season, and permanent frost remains only in different depths beneath. Moreover, it has been suggested that thermophilic biota have persisted in sheltered valleys with suitable microclimates, and there is hard evidence for some northern refugia up to the Norwegian coast^55,77^. For instance, forest land snails have been recorded from the late glacial of the Bohemian Massif, providing evidence for the persistence of small patches of broadleaf forest in Central Europe^78^.

In conclusion (Fig. 10), our study supports the existence of extra-Mediterranean refugia in grass snakes. The refugium of the blue lineage of *N. helvetica* was most likely located in southern France, outside the Mediterranean peninsulas. The red lineage of *N. natrix* seems to have survived the last glacial in a classical southern refugium in the Balkan Peninsula. Haplotype networks, mismatch distributions and demographic analyses of mtDNA sequences suggest that its refuge was geographically structured. From the Balkan, the red lineage has spread late to Central Europe and the Carpathian Basin and admixed en route with the yellow lineage of *N. natrix*^11^. For the yellow lineage, there is strong evidence for two distinct refugia during the LGM, one in the southern Balkan Peninsula and another one in Central Europe. This is supported (1) by the wide distribution gap in the northern Balkan Peninsula, (2) largely distinct mitochondrial haplotypes of grass snakes from the disjunct southern and northern parts of the distribution range, (3) by the higher genetic diversity of grass snakes from northern Germany and Scandinavia compared to snakes from southern Central Europe, (4) the lack of the red lineage in Scandinavia and northern Central Europe, and (5) by modelling using Approximate Bayesian Computation. From the Central European refugium, the yellow lineage colonized Scandinavia during the Holocene climatic optimum, long before the red lineage arrived in Central Europe.

## Electronic supplementary material

Supplementary Information
