# Supplementary material for: Extra-Mediterranean glacial refuges in barred and common grass snakes (Natrix helvetica, N. natrix)
Source: Sci Rep. 2018 Jan 29;8:1821. doi: 10.1038/s41598-018-20218-2 (PMC5788984; doi:10.1038/s41598-018-20218-2)
Supplement: Supplementary file 1 — Supplementary Information [file 41598_2018_20218_MOESM1_ESM.pdf]

Extra-Mediterranean glacial refuges in barred and common grass snakes *(Natrix helvetica, N. natrix)*

Carolin Kindler, Eva Graciá & Uwe Fritz

Scientific Reports

DOI:10.1038/s41598-018-20218-2

Supplementary Information

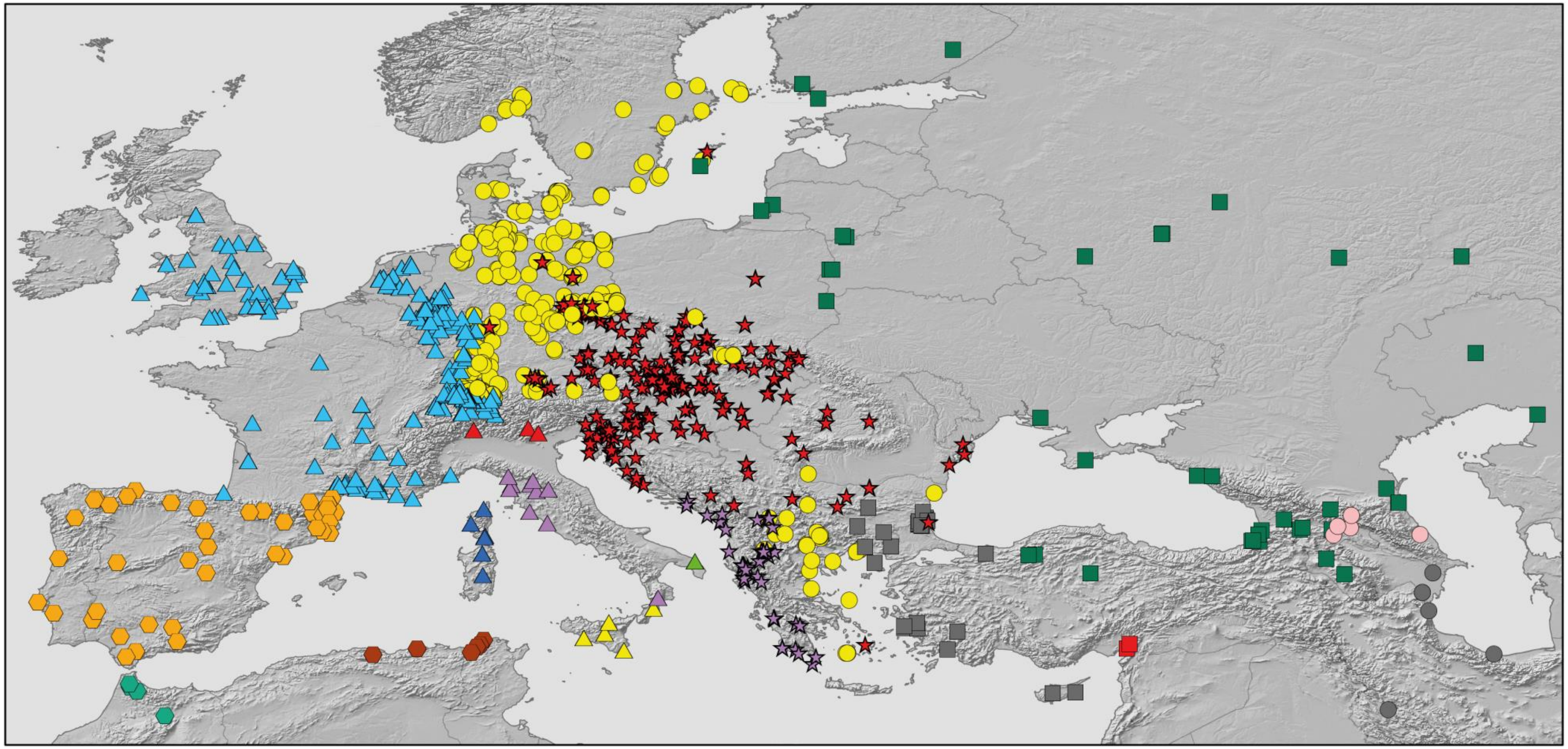

Figure S1. Distribution of mitochondrial lineages of *Natrix astreptophora*, *N. helvetica*, and *N. natrix* (combined from Kindler *et al.* 2013, 2014, 2017, 2018; Pokrant *et al.* 2016, and this study). Different mitochondrial lineages are indicated by different colours and symbols. Hexagons correspond to *N. astreptophora*; triangles, *N. helvetica*; remaining symbols, *N. natrix*. The Iberian Peninsula is occupied by *N. astreptophora* and the Apennine Peninsula and the Po drainage basin, by endemic lineages of *N. helvetica*. Note the many different lineages of *N. natrix* on the southern Balkan Peninsula contributing to massive nuclear admixture in this region. Non-native records disregarded. Map was created using ARCGIS 10.2 (www.esri.com/arcgis) and ADOBE ILLUSTRATOR CS6 (www.adobe.com/products/illustrator.html).

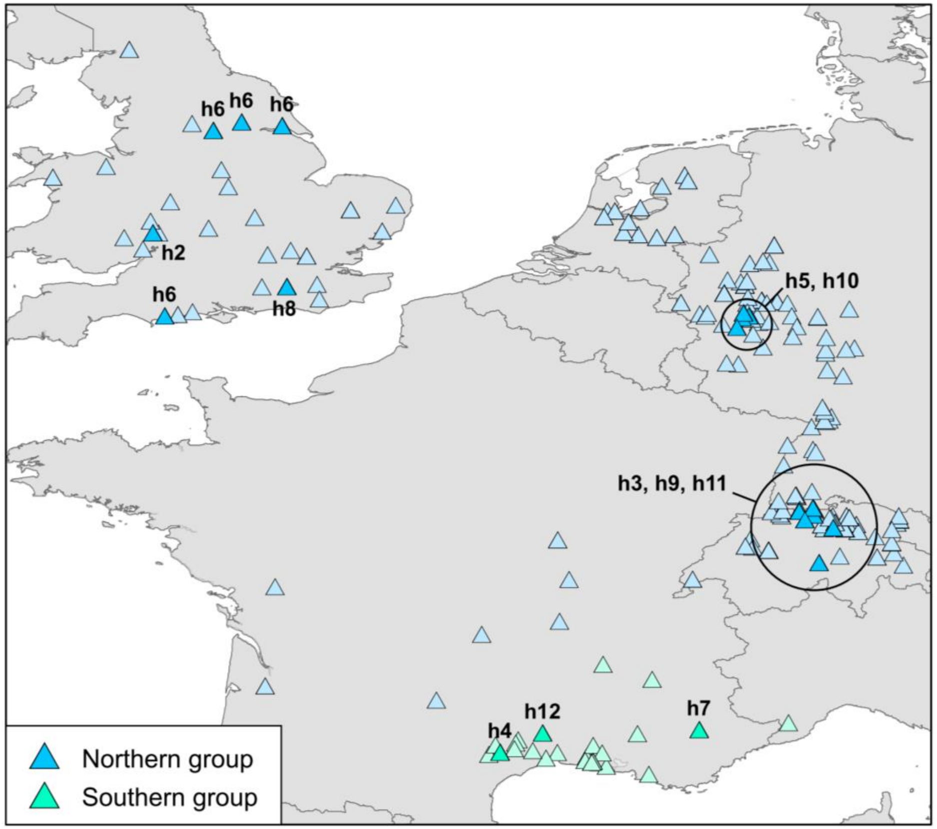

Figure S2. Geographic distribution of haplotypes of the blue lineage. Pale symbols indicate the central haplotype h1; more intensely coloured symbols, all other haplotypes. Map was created using ARCGIS 10.2 (www.esri.com/arcgis) and ADOBE ILLUSTRATOR CS6 (www.adobe.com/products/illustrator.html).

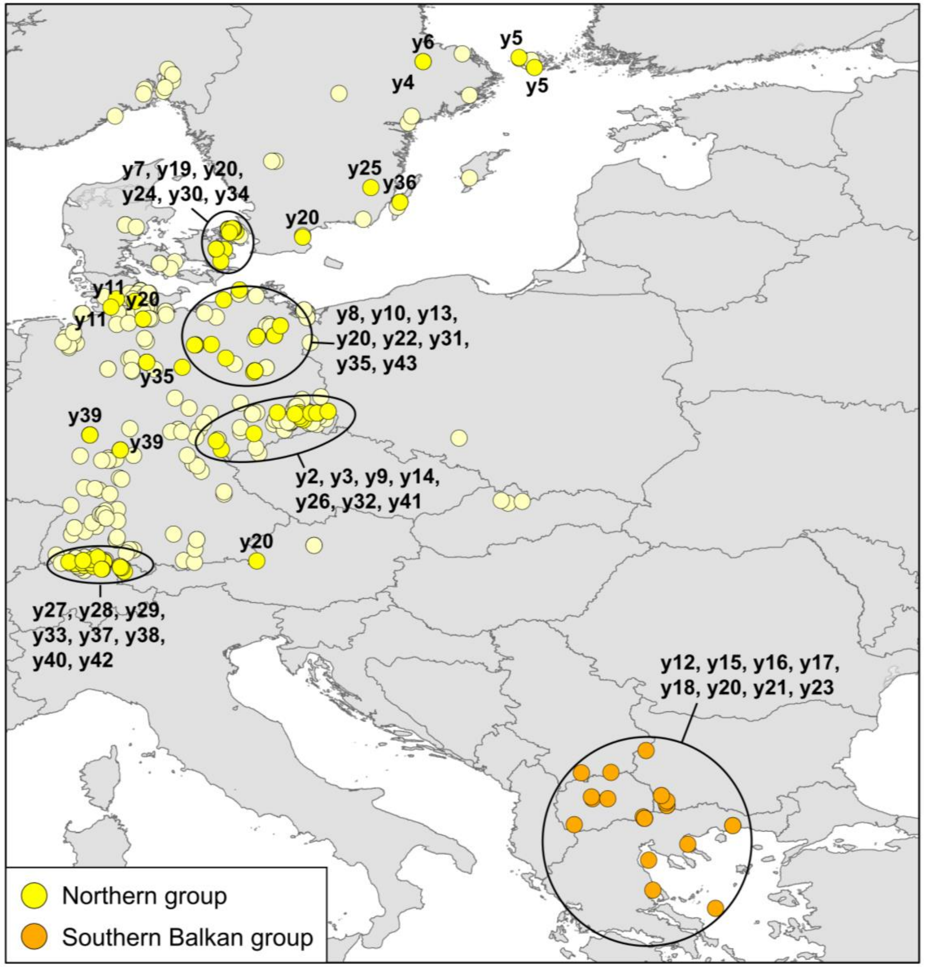

Figure S3. Geographic distribution of haplotypes of the yellow lineage. Pale symbols indicate the central haplotype y1; more intensely coloured symbols, all other haplotypes. Map was created using ARCGIS 10.2 (www.esri.com/arcgis) and ADOBE ILLUSTRATOR CS6 (www.adobe.com/products/illustrator.html).

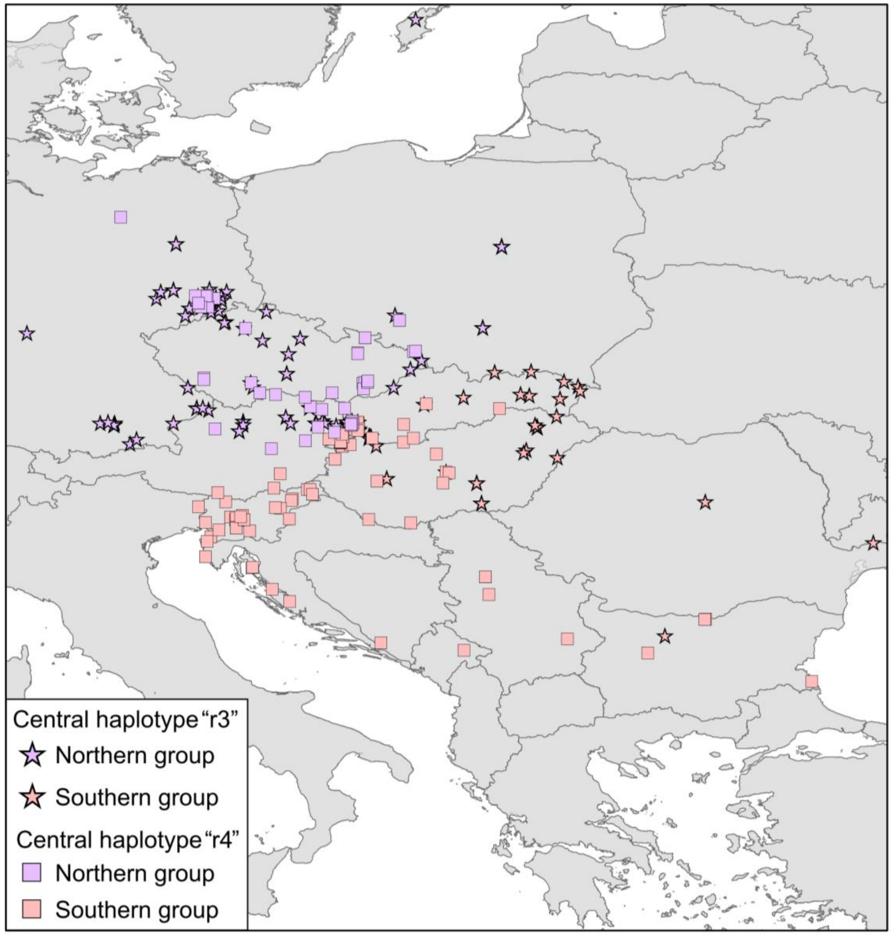

Figure S4. Geographic distribution of both central haplotypes of the red lineage. Map was created using ARCGIS 10.2 (www.esri.com/arcgis) and ADOBE ILLUSTRATOR CS6 (www.adobe.com/products/illustrator.html).

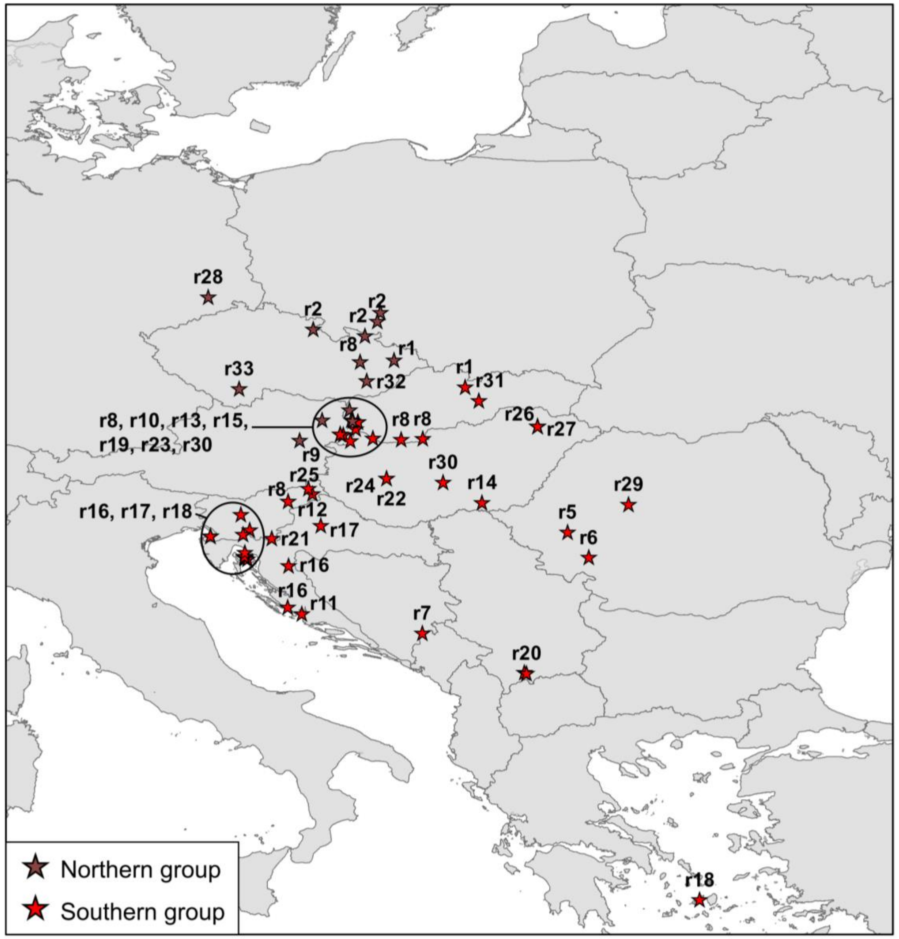

Figure S5. Geographic distribution of haplotypes of the red lineage (exept both central haplotypes). Map was created using ARCGIS 10.2 (www.esri.com/arcgis) and ADOBE ILLUSTRATOR CS6 (www.adobe.com/products/illustrator.html).

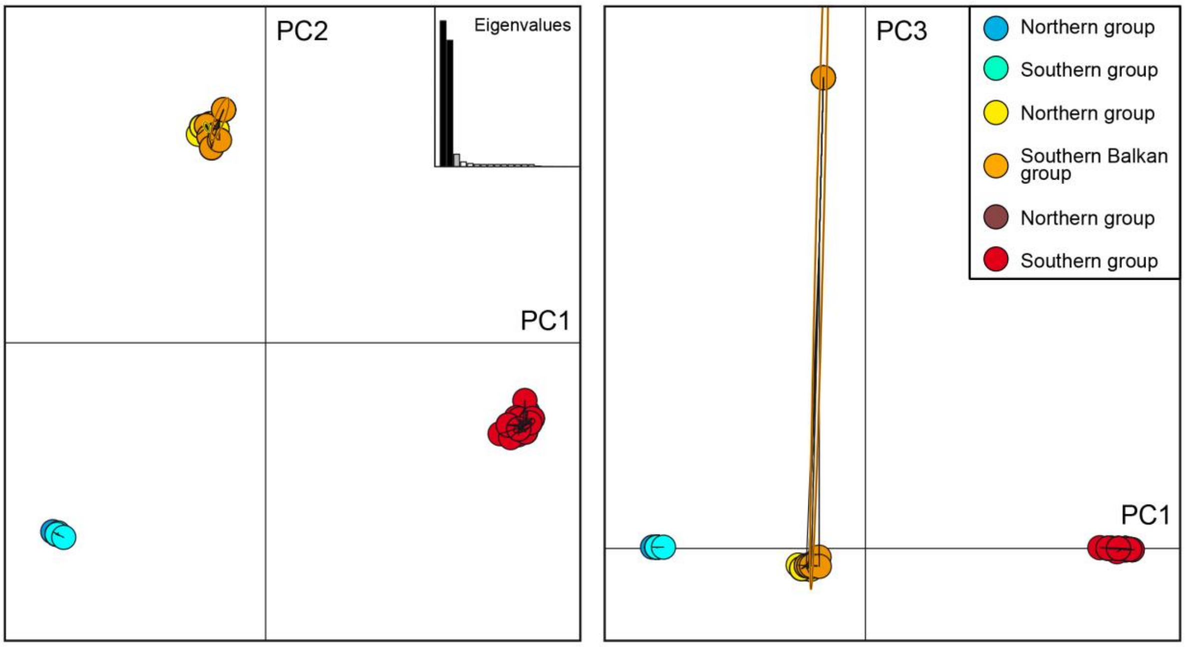

Figure S6. Principal Component Analysis (PCA) for 1,372 mitochondrial DNA sequences of grass snakes and 95% confidential intervals (ovals) for the three lineages blue, yellow and red. The first, second and third principal components (PC1–3) explain 72.0%, 62.4%, and 6.1% of the observed variance.

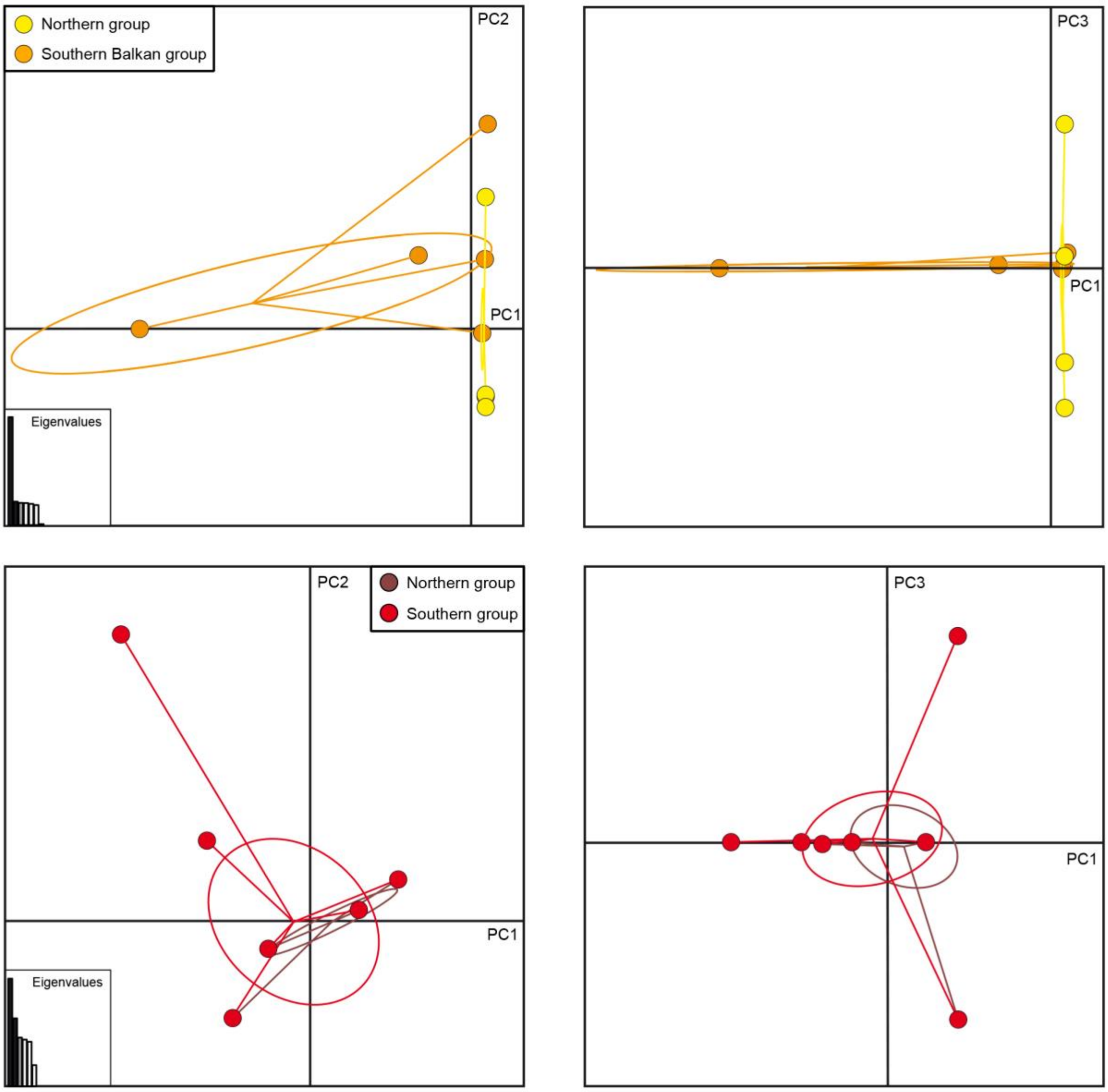

Figure S7. Principal Component Analysis (PCA) for 526 mitochondrial DNA sequences of the yellow lineage (top) and 443 sequences of the red lineage (bottom) and 95% confidential intervals (ovals) for groups within these lineages. For the yellow lineage, PC1–3 explain 9.9%, 2.2%, and 2.0% of the observed variance. For the red lineage, PC1–3 explain 4.5%, 2.8%, and 2.0% of the observed variance. For the blue lineage (not shown), only PC1–2 could be calculated due to massive overlap, with each PC explaining 2.0% of the observed variance. Due to similarity of sequences and haplotypes, individual values appear only as few dots despite large samples.

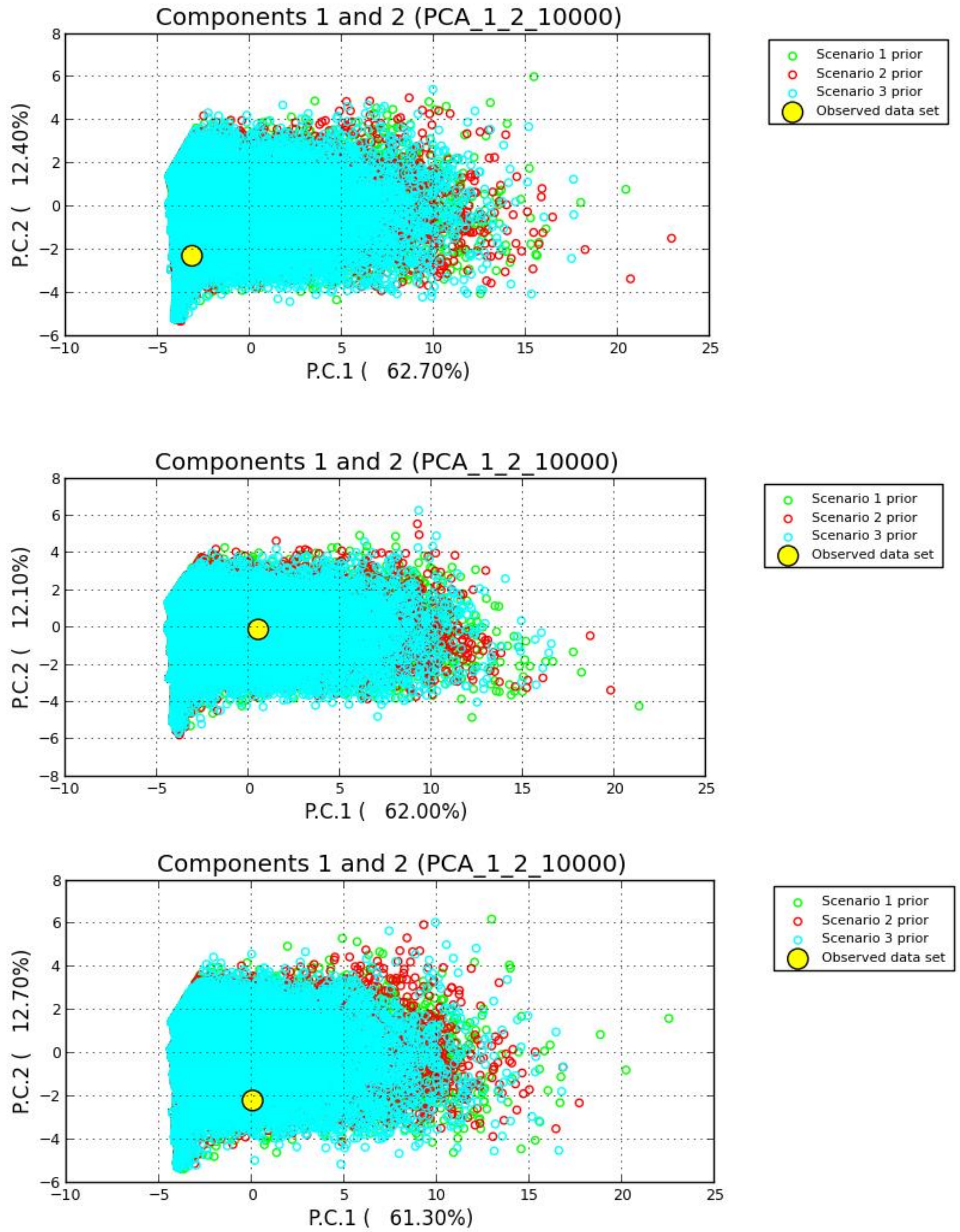

Figure S8. Principal Component Analysis (PCA) plots to pre-evaluate scenario-prior combinations in DIYABC 2.0.4 (Cornuet *et al.* 2014): (a) *Natrix helvetica* (blue lineage), (b) yellow lineage of *N. natrix*, and (c) red lineage of *N. natrix*. The large yellow dots represent where the observed data fits within a representative set of 10,000 simulated datasets per scenario (small dots). Since the observed data fall within the clouds of simulated data, it is possible to produce summary statistics of demographic parameters for the proposed scenarios (divergence time among populations).

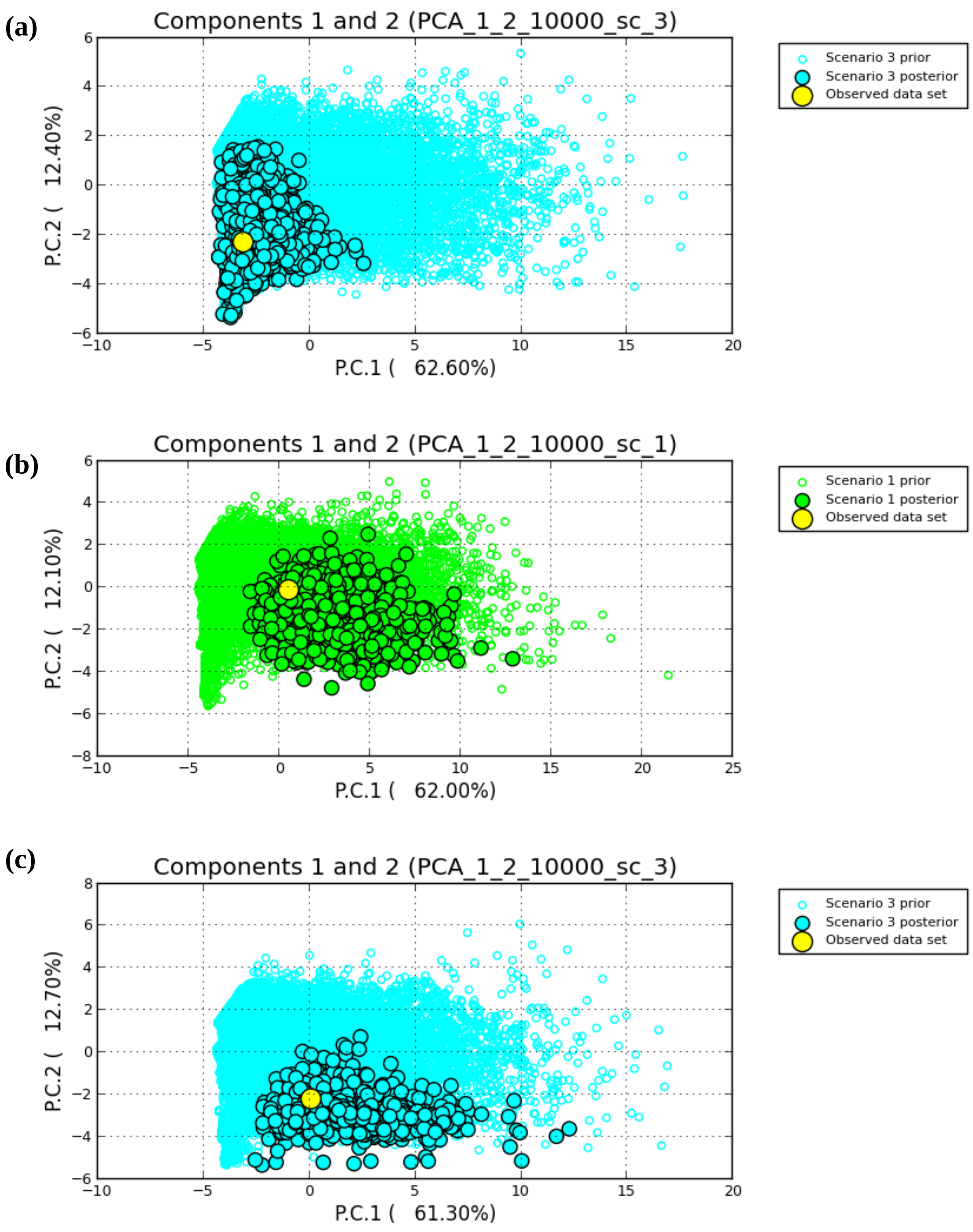

Figure S9. Principal Component Analysis (PCA) plots to perform model checking in DIYABC 2.0.4 (Cornuet *et al.* 2014): (a) *Natrix helvetica* (blue lineage), (b) yellow lineage of *N. natrix*, and (c) red lineage of *N. natrix*. The large yellow dots represent where the observed data fits within a representative set of 10,000 simulated datasets (small blue and green dots) and the posterior predictive distributions (larger blue and green dots).

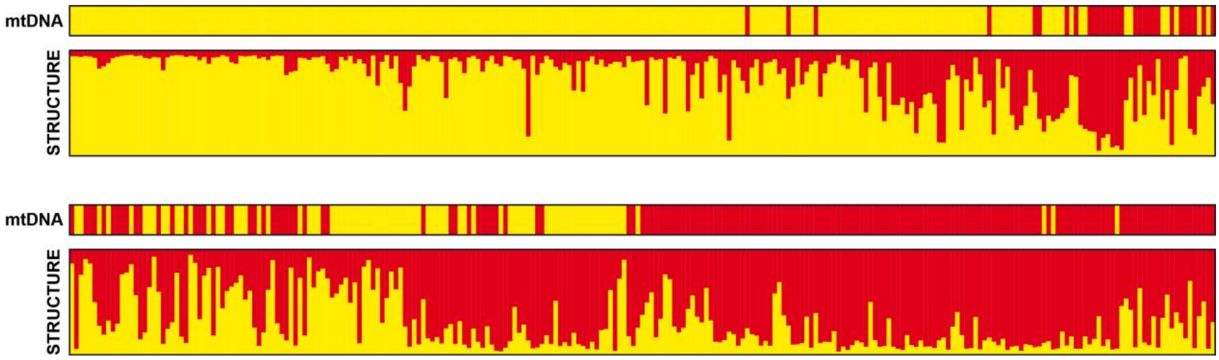

Figure S10. Genotypic structuring of 502 samples of the yellow and red lineages of common grass snakes (*Natrix natrix*) inferred by STRUCTURE 2.3.4 (Falush *et al.* 2003; Pritchard *et al.* 2000) using 13 microsatellite loci, the admixture model and correlated allele frequencies. Individual samples correspond to vertical bars indicating the mitochondrial haplotype (top) and the inferred ancestry from STRUCTURE (bottom). Samples are arranged geographically from northern Europe (top left) to southeastern Europe (bottom right). Data were pruned in a step-wise process from genetic impact of other geographically neighbouring lineages of the southern Balkan Peninsula (for details, see Kindler *et al*. 2017). The ancestral polymorphism hypothesis for the yellow and red lineages is to be rejected because the mitochondrial lineages match with nuclear genomic clusters. If the two mitochondrial lineages would have occurred in one and the same refuge, only one nuclear cluster were expected. Redrawn from Kindler *et al.* (2017).

Table S1. Used grass snake samples with corresponding European Nucleotide Archive (ENA) accession numbers. NCESS = Northern Central European/Scandinavian Subgroup, SCES = Southern Central European Subgroup.

Voucher	Locality	Haplotype	Accession number	Reference	Geographical division	
					Group	Subgroup
**BEV.1690**	France: Alpes-de-Haute-Provence: Clumanc	**h7**	LT839098	Pokrant *et al.* (2016)	southern group	—
**ZFMK 54711**	France: Ardèche: 10 km N Tournon-sur-Rhône	**h1**	LT839092	Kindler *et al.* (2013)	southern group	—
**BEV.6430**	France: Aveyron: Peyrusse-le-Roc	**h1**	LT839092	Pokrant *et al.* (2016)	northern group	—
**MTD T 10091**	France: Bas-Rhin: Strasbourg	**h1**	LT839092	Kindler *et al.* (2013)	northern group	—
**BEV.11494**	France: Bouches-du-Rhône: Aubagne	**h1**	LT839092	Kindler *et al.* (2017)	southern group	—
**BEV.9397**	France: Bouches-du-Rhône: between Fos-sur-Mer and Port-Saint-Louis-du-Rhône	**h1**	LT839092	Pokrant *et al.* (2016)	southern group	—
**ZFMK 54712**	France: Bouches-du-Rhône: Camargue	**h1**	LT839092	Kindler *et al.* (2013)	southern group	—
**BEV.6425**	France: Bouches-du-Rhône: Camargue: Clos de Lange	**h1**	LT839092	Pokrant *et al.* (2016)	southern group	—
**BEV.284**	France: Bouches-du-Rhône: Camargue: Marais du Grenouillet	**h1**	LT839092	Pokrant *et al.* (2016)	southern group	—
**ZMH R07158**	France: Bouches-du-Rhône: Camargue: near Fiélouse	**h1**	LT839092	Kindler *et al.* (2017)	southern group	—
**ZMH R07572**	France: Bouches-du-Rhône: Camargue: Salin de Badon	**h1**	LT839092	Pokrant *et al.* (2016)	southern group	—
**ZMH R07573**	France: Bouches-du-Rhône: Camargue: Salin de Badon	**h1**	LT839092	Pokrant *et al.* (2016)	southern group	—
**BEV.9398**	France: Bouches-du-Rhône: Camargue: Salin de Badon	**h1**	LT839092	Kindler *et al.* (2017)	southern group	—
**ZMH R07062**	France: Bouches-du-Rhône: Camargue: Tour du Valat	**h1**	LT839092	Pokrant *et al.* (2016)	southern group	—
**ZMH R06997**	France: Bouches-du-Rhône: Camargue: Tour du Valat	**h1**	LT839092	Pokrant *et al.* (2016)	southern group	—
**ZMH R06950**	France: Bouches-du-Rhône: Camargue: Tour du Valat	**h1**	LT839092	Pokrant *et al.* (2016)	southern group	—
**ZMH R06951**	France: Bouches-du-Rhône: Camargue: Tour du Valat	**h1**	LT839092	Pokrant *et al.* (2016)	southern group	—
**ZMH R06952**	France: Bouches-du-Rhône: Camargue: Tour du Valat	**h1**	LT839092	Pokrant *et al.* (2016)	southern group	—
**ZFMK 54710**	France: Bouches-du-Rhône: Saint-Martin-de-Crau	**h1**	LT839092	Kindler *et al.* (2013)	southern group	—
**BEV.9401**	France: Bouches-du-Rhône: Saint-Martin-de-Crau: Mas de Pernes	**h1**	LT839092	Pokrant *et al.* (2016)	southern group	—
**MTD T 11950**	France: Deux-Sèvres: near Niort	**h1**	LT839092	Pokrant *et al.* (2016)	northern group	—
**BEV.9400**	France: Gard: Saint-Hippolyte-du-Fort	**h12**	LT839103	Pokrant *et al.* (2016)	southern group	—
**BEV.T9260**	France: Gironde: near Léognan: Minoy	**h1**	LT839092	Pokrant *et al.* (2016)	northern group	—
**BEV.T9261**	France: Gironde: near Léognan: Minoy	**h1**	LT839092	Pokrant *et al.* (2016)	northern group	—
**BEV.T9264**	France: Hérault: Avène dam	**h1**	LT839092	Pokrant *et al.* (2016)	southern group	—
**BEV.9049**	France: Hérault: between Le Bosc and Loiras	**h1**	LT839092	Pokrant *et al.* (2016)	southern group	—
**BEV.9029**	France: Hérault: Lansargues	**h1**	LT839092	Pokrant *et al.* (2016)	southern group	—
**BEV.T9266**	France: Hérault: Lattes	**h1**	LT839092	Pokrant *et al.* (2016)	southern group	—
**BEV.T9259**	France: Hérault: Le Mas Blanc	**h1**	LT839092	Pokrant *et al.* (2016)	southern group	—
**BEV.T9258**	France: Hérault: Le Mas Blanc	**h4**	LT839095	Pokrant *et al.* (2016)	southern group	—
**BEV.T9565**	France: Hérault: Les Corrèges	**h1**	LT839092	Kindler *et al.* (2017)	southern group	—
**BEV.8499**	France: Hérault: Massif du Caroux	**h1**	LT839092	Pokrant *et al.* (2016)	southern group	—
**BEV.847**	France: Hérault: Saint-Pierre-de-la-Fage	**h1**	LT839092	Pokrant *et al.* (2016)	southern group	—
**BEV.10974**	France: Hérault: Soulagets	**h1**	LT839092	Kindler *et al.* (2017)	southern group	—
**BEV.9293**	France: Isère: Clelles	**h1**	LT839092	Pokrant *et al.* (2016)	southern group	—
**BEV.8324**	France: Loire: Sainte-Foy-Saint-Sulpice	**h1**	LT839092	Pokrant *et al.* (2016)	northern group	—
**MTD T 10092**	France: Puy-de-Dôme: near Murol: Monneaux	**h1**	LT839092	Kindler *et al.* (2013)	northern group	—
**MTD T 11999**	France: Pyrénées-Atlantiques: St. Jean de Luz	**h1**	LT839092	Pokrant *et al.* (2016)	southern group	—
**ZFMK 61095**	France: Saône-et-Loire: Anost	**h1**	LT839092	Kindler *et al.* (2013)	northern group	—
**MTD T 12024**	France: Saône-et-Loire: Anost	**h1**	LT839092	Kindler *et al.* (2017)	northern group	—

Table S1 continued						
**Voucher**	**Locality**	**Haplotype**	**Accession number**	**Reference**	**Geographical division**	
					**Group**	**Subgroup**
**BEV.11530**	France: Vaucluse: Saint-Saturnin-lès-Apt	**h1**	LT839092	Pokrant *et al.* (2016)	southern group	—
**SMNS 14503**	Germany: Baden-Württemberg: Dettenheim	**h1**	LT839092	Kindler *et al.* (2017)	northern group	—
**MTD T 14828**	Germany: Baden-Württemberg: Harpolingen	**h1**	LT839092	Kindler *et al.* (2017)	northern group	—
**SMNS 14505**	Germany: Baden-Württemberg: Herten	**h1**	LT839092	Kindler *et al.* (2017)	northern group	—
**SMNS 14512**	Germany: Baden-Württemberg: Kandern	**h1**	LT839092	Kindler *et al.* (2017)	northern group	—
**MTD T 14874**	Germany: Baden-Württemberg: Kappel-Grafenhausen	**h1**	LT839092	Kindler *et al.* (2017)	northern group	—
**SMNK-REP 689**	Germany: Baden-Württemberg: Karlsruhe	**h1**	LT839092	Kindler *et al.* (2017)	northern group	—
**SMNK-REP 910**	Germany: Baden-Württemberg: Karlsruhe	**h1**	LT839092	Kindler *et al.* (2017)	northern group	—
**MTD T 14841**	Germany: Baden-Württemberg: Karlsruhe	**h1**	LT839092	Kindler *et al.* (2017)	northern group	—
**SMNK-REP 1003**	Germany: Baden-Württemberg: Karlsruhe: between Staffort and Weingarten	**h1**	LT839092	Kindler *et al.* (2017)	northern group	—
**MTD T 14813**	Germany: Baden-Württemberg: Karlsruhe: Oberwaldsee	**h1**	LT839092	Kindler *et al.* (2017)	northern group	—
**MTD T 14821**	Germany: Baden-Württemberg: Kniebis	**h1**	LT839092	Kindler *et al.* (2017)	northern group	—
**MTD T 14820**	Germany: Baden-Württemberg: Lenzkirch	**h1**	LT839092	Kindler *et al.* (2017)	northern group	—
**SMNK-REP 1268**	Germany: Baden-Württemberg: Linkenheim	**h1**	LT839092	Kindler *et al.* (2017)	northern group	—
**MTD T 14818**	Germany: Baden-Württemberg: Ottenhöfen	**h1**	LT839092	Kindler *et al.* (2017)	northern group	—
**MTD T 14823**	Germany: Baden-Württemberg: Präg	**h1**	LT839092	Kindler *et al.* (2017)	northern group	—
**MTD T 14824**	Germany: Baden-Württemberg: Präg	**h1**	LT839092	Kindler *et al.* (2017)	northern group	—
**MTD T 14825**	Germany: Baden-Württemberg: Präg	**h1**	LT839092	Kindler *et al.* (2017)	northern group	—
**MTD T 14826**	Germany: Baden-Württemberg: Präg	**h1**	LT839092	Kindler *et al.* (2017)	northern group	—
**MTD T 14827**	Germany: Baden-Württemberg: Präg	**h1**	LT839092	Kindler *et al.* (2017)	northern group	—
**MTD T 14839**	Germany: Baden-Württemberg: Steinmauern	**h1**	LT839092	Kindler *et al.* (2017)	northern group	—
**SMF 76440**	Germany: Hesse: Bergen-Enkheim	**h1**	LT839092	Kindler *et al.* (2017)	northern group	—
**ZFMK 89403**	Germany: Hesse: Herborn	**h1**	LT839092	Kindler *et al.* (2013)	northern group	—
**MTD T 14112**	Germany: Hesse: Herborn	**h1**	LT839092	Kindler *et al.* (2017)	northern group	—
**MTD D 35776**	Germany: Hesse: Hofheim/Lorsbuch	**h1**	LT839092	Kindler *et al.* (2013)	northern group	—
**MTD T 14175**	Germany: Hesse: Lautertal-Beedenkirchen	**h1**	LT839092	Kindler *et al.* (2017)	northern group	—
**ZSUM Re.S2.1.2.c**	Germany: Hesse: Marburg	**h1**	LT839092	Kindler *et al.* (2017)	northern group	—
**SMF 72196**	Germany: Hesse: near Hanau: Mittelbuchen	**h1**	LT839092	Kindler *et al.* (2017)	northern group	—
**SMF 72456**	Germany: Hesse: near Kelkheim: Fischbach	**h1**	LT839092	Kindler *et al.* (2017)	northern group	—
**SMF 75825**	Germany: Hesse: SW Gross-Gerau: Kühkopf	**h1**	LT839092	Kindler *et al.* (2017)	northern group	—
**SMF 78703**	Germany: Hesse: Weilrod: Niederlauken	**h1**	LT839092	Kindler *et al.* (2017)	northern group	—
**ZFMK 68431**	Germany: North Rhine-Westphalia: Bad Honnef	**h1**	LT839092	Kindler *et al.* (2013)	northern group	—
**ZFMK 68432**	Germany: North Rhine-Westphalia: Bad Honnef	**h1**	LT839092	Kindler *et al.* (2013)	northern group	—
**ZFMK 92193**	Germany: North Rhine-Westphalia: Bad Münstereifel	**h1**	LT839092	Kindler *et al.* (2013)	northern group	—
**MTD T 11191**	Germany: North Rhine-Westphalia: between Ammelsbüren and Davensberg	**h1**	LT839092	Kindler *et al.* (2017)	northern group	—
**MTD T 11193**	Germany: North Rhine-Westphalia: between Ammelsbüren and Davensberg	**h1**	LT839092	Kindler *et al.* (2017)	northern group	—
**MTD T 11194**	Germany: North Rhine-Westphalia: between Ammelsbüren and Davensberg	**h1**	LT839092	Kindler *et al.* (2017)	northern group	—
**MTD T 11195**	Germany: North Rhine-Westphalia: between Ammelsbüren and Davensberg	**h1**	LT839092	Kindler *et al.* (2017)	northern group	—
**MTD T 11196**	Germany: North Rhine-Westphalia: between Ammelsbüren and Davensberg	**h1**	LT839092	Kindler *et al.* (2017)	northern group	—
**MTD T 11197**	Germany: North Rhine-Westphalia: between Ammelsbüren and Davensberg	**h1**	LT839092	Kindler *et al.* (2017)	northern group	—
**MTD T 11198**	Germany: North Rhine-Westphalia: between Ammelsbüren and Davensberg	**h1**	LT839092	Kindler *et al.* (2017)	northern group	—
**MTD T 11199**	Germany: North Rhine-Westphalia: between Ammelsbüren and Davensberg	**h1**	LT839092	Kindler *et al.* (2017)	northern group	—

Table S1 continued						
**Voucher**	**Locality**	**Haplotype**	**Accession number**	**Reference**	**Geographical division**	
					**Group**	**Subgroup**
**MTD T 11200**	Germany: North Rhine-Westphalia: between Ammelsbüren and Davensberg	**h1**	LT839092	Kindler *et al.* (2017)	northern group	—
**MTD T 11204**	Germany: North Rhine-Westphalia: between Ammelsbüren and Davensberg	**h1**	LT839092	Kindler *et al.* (2017)	northern group	—
**MTD T 11408**	Germany: North Rhine-Westphalia: between Ammelsbüren and Davensberg	**h1**	LT839092	Kindler *et al.* (2017)	northern group	—
**MTD T 11409**	Germany: North Rhine-Westphalia: between Ammelsbüren and Davensberg	**h1**	LT839092	Kindler *et al.* (2017)	northern group	—
**MTD T 11412**	Germany: North Rhine-Westphalia: between Ammelsbüren and Davensberg	**h1**	LT839092	Kindler *et al.* (2017)	northern group	—
**MTD T 11414**	Germany: North Rhine-Westphalia: between Ammelsbüren and Davensberg	**h1**	LT839092	Kindler *et al.* (2017)	northern group	—
**MTD T 11415**	Germany: North Rhine-Westphalia: between Ammelsbüren and Davensberg	**h1**	LT839092	Kindler *et al.* (2017)	northern group	—
**MTD T 11417**	Germany: North Rhine-Westphalia: between Ammelsbüren and Davensberg	**h1**	LT839092	Kindler *et al.* (2017)	northern group	—
**MTD T 11420**	Germany: North Rhine-Westphalia: between Ammelsbüren and Davensberg	**h1**	LT839092	Kindler *et al.* (2017)	northern group	—
**MTD T 11421**	Germany: North Rhine-Westphalia: between Ammelsbüren and Davensberg	**h1**	LT839092	Kindler *et al.* (2017)	northern group	—
**MTD T 11422**	Germany: North Rhine-Westphalia: between Ammelsbüren and Davensberg	**h1**	LT839092	Kindler *et al.* (2017)	northern group	—
**MTD T 11424**	Germany: North Rhine-Westphalia: between Ammelsbüren and Davensberg	**h1**	LT839092	Kindler *et al.* (2017)	northern group	—
**MTD T 11426**	Germany: North Rhine-Westphalia: between Ammelsbüren and Davensberg	**h1**	LT839092	Kindler *et al.* (2017)	northern group	—
**MTD T 11427**	Germany: North Rhine-Westphalia: between Ammelsbüren and Davensberg	**h1**	LT839092	Kindler *et al.* (2017)	northern group	—
**MTD T 11429**	Germany: North Rhine-Westphalia: between Ammelsbüren and Davensberg	**h1**	LT839092	Kindler *et al.* (2017)	northern group	—
**MTD T 11430**	Germany: North Rhine-Westphalia: between Ammelsbüren and Davensberg	**h1**	LT839092	Kindler *et al.* (2017)	northern group	—
**MTD T 11432**	Germany: North Rhine-Westphalia: between Ammelsbüren and Davensberg	**h1**	LT839092	Kindler *et al.* (2017)	northern group	—
**ZFMK 75088**	Germany: North Rhine-Westphalia: between Bergisch-Gladbach and Herrenstrunden	**h1**	LT839092	Kindler *et al.* (2013)	northern group	—
**ZFMK 70420**	Germany: North Rhine-Westphalia: between Hürtgenwald and Gey	**h1**	LT839092	Kindler *et al.* (2013)	northern group	—
**MTD T 12494**	Germany: North Rhine-Westphalia: between Ratingen and Düsseldorf	**h1**	LT839092	Kindler *et al.* (2017)	northern group	—
**ZFMK 92536**	Germany: North Rhine-Westphalia: Bonn	**h1**	LT839092	Kindler *et al.* (2013)	northern group	—
**ZFMK 92228**	Germany: North Rhine-Westphalia: Bonn	**h1**	LT839092	Kindler *et al.* (2013)	northern group	—
**ZFMK 68433**	Germany: North Rhine-Westphalia: Bonn	**h10**	LT839101	Kindler *et al.* (2013)	northern group	—
**ZFMK 92537**	Germany: North Rhine-Westphalia: Bonn	**h10**	LT839101	Kindler *et al.* (2013)	northern group	—
**ZFMK 89393**	Germany: North Rhine-Westphalia: Bonn: Kottenforst	**h10**	LT839101	Kindler *et al.* (2013)	northern group	—
**MTD T 12106**	Germany: North Rhine-Westphalia: Brüchermühle	**h1**	LT839092	Kindler *et al.* (2017)	northern group	—
**MTD T 12107**	Germany: North Rhine-Westphalia: Brüchermühle	**h1**	LT839092	Kindler *et al.* (2017)	northern group	—
**MTD T 12108**	Germany: North Rhine-Westphalia: Brüchermühle	**h1**	LT839092	Kindler *et al.* (2017)	northern group	—
**MTD T 12109**	Germany: North Rhine-Westphalia: Brüchermühle	**h1**	LT839092	Kindler *et al.* (2017)	northern group	—
**MTD T 12110**	Germany: North Rhine-Westphalia: Brüchermühle	**h1**	LT839092	Kindler *et al.* (2017)	northern group	—
**MTD T 12111**	Germany: North Rhine-Westphalia: Brüchermühle	**h1**	LT839092	Kindler *et al.* (2017)	northern group	—
**MTD T 12112**	Germany: North Rhine-Westphalia: Brüchermühle	**h1**	LT839092	Kindler *et al.* (2017)	northern group	—
**MTD T 12115**	Germany: North Rhine-Westphalia: Brüchermühle	**h1**	LT839092	Kindler *et al.* (2017)	northern group	—
**MTD T 12117**	Germany: North Rhine-Westphalia: Brüchermühle	**h1**	LT839092	Kindler *et al.* (2017)	northern group	—
**MTD T 12118**	Germany: North Rhine-Westphalia: Brüchermühle	**h1**	LT839092	Kindler *et al.* (2017)	northern group	—
**MTD T 12119**	Germany: North Rhine-Westphalia: Brüchermühle	**h1**	LT839092	Kindler *et al.* (2017)	northern group	—
**MTD T 12120**	Germany: North Rhine-Westphalia: Brüchermühle	**h1**	LT839092	Kindler *et al.* (2017)	northern group	—
**MTD T 12121**	Germany: North Rhine-Westphalia: Brüchermühle	**h1**	LT839092	Kindler *et al.* (2017)	northern group	—
**MTD T 12122**	Germany: North Rhine-Westphalia: Brüchermühle	**h1**	LT839092	Kindler *et al.* (2017)	northern group	—
**MTD T 12123**	Germany: North Rhine-Westphalia: Brüchermühle	**h1**	LT839092	Kindler *et al.* (2017)	northern group	—
**MTD T 12124**	Germany: North Rhine-Westphalia: Brüchermühle	**h1**	LT839092	Kindler *et al.* (2017)	northern group	—
**MTD T 12125**	Germany: North Rhine-Westphalia: Brüchermühle	**h1**	LT839092	Kindler *et al.* (2017)	northern group	—
**MTD T 12126**	Germany: North Rhine-Westphalia: Brüchermühle	**h1**	LT839092	Kindler *et al.* (2017)	northern group	—
**MTD T 12127**	Germany: North Rhine-Westphalia: Brüchermühle	**h1**	LT839092	Kindler *et al.* (2017)	northern group	—

Table S1 continued						
Voucher	Locality	Haplotype	Accession number	Reference	Geographical division	
					Group	Subgroup
**MTD T 12128**	Germany: North Rhine-Westphalia: Brüchermühle	**h1**	LT839092	Kindler *et al.* (2017)	northern group	—
**MTD T 14059**	Germany: North Rhine-Westphalia: Dortmund	**h1**	LT839092	Kindler *et al.* (2017)	northern group	—
**MTD T 14060**	Germany: North Rhine-Westphalia: Dortmund	**h1**	LT839092	Kindler *et al.* (2017)	northern group	—
**MTD T 11981**	Germany: North Rhine-Westphalia: Dortmund: Kirchderne	**h1**	LT839092	Kindler *et al.* (2017)	northern group	—
**ZFMK 86786**	Germany: North Rhine-Westphalia: Drove	**h1**	LT839092	Kindler *et al.* (2013)	northern group	—
**SMNS 14502**	Germany: North Rhine-Westphalia: Herfterath	**h1**	LT839092	Kindler *et al.* (2017)	northern group	—
**MTD T 11786**	Germany: North Rhine-Westphalia: Herne	**h1**	LT839092	Kindler *et al.* (2017)	northern group	—
**MTD T 12021**	Germany: North Rhine-Westphalia: Herne	**h1**	LT839092	Kindler *et al.* (2017)	northern group	—
**MTD T 12020**	Germany: North Rhine-Westphalia: Königswinter	**h10**	LT839101	Kindler *et al.* (2017)	northern group	—
**ZFMK 82773**	Germany: North Rhine-Westphalia: Königswinter: Ittenbach	**h1**	LT839092	Kindler *et al.* (2013)	northern group	—
**MTD T 11574**	Germany: North Rhine-Westphalia: Much	**h1**	LT839092	Kindler *et al.* (2017)	northern group	—
**MTD T 11575**	Germany: North Rhine-Westphalia: Much	**h1**	LT839092	Kindler *et al.* (2017)	northern group	—
**MTD T 11576**	Germany: North Rhine-Westphalia: Much	**h1**	LT839092	Kindler *et al.* (2017)	northern group	—
**MTD T 11577**	Germany: North Rhine-Westphalia: Much	**h1**	LT839092	Kindler *et al.* (2017)	northern group	—
**MTD T 14087**	Germany: North Rhine-Westphalia: near Dormagen: Knechtstedener Wald	**h1**	LT839092	Kindler *et al.* (2017)	northern group	—
**MTD T 14088**	Germany: North Rhine-Westphalia: near Dormagen: Knechtstedener Wald	**h1**	LT839092	Kindler *et al.* (2017)	northern group	—
**MTD T 14089**	Germany: North Rhine-Westphalia: near Dormagen: Knechtstedener Wald	**h1**	LT839092	Kindler *et al.* (2017)	northern group	—
**MTD T 14091**	Germany: North Rhine-Westphalia: near Dormagen: Knechtstedener Wald	**h1**	LT839092	Kindler *et al.* (2017)	northern group	—
**MTD T 14092**	Germany: North Rhine-Westphalia: near Dormagen: Knechtstedener Wald	**h1**	LT839092	Kindler *et al.* (2017)	northern group	—
**MTD T 14094**	Germany: North Rhine-Westphalia: near Dormagen: Knechtstedener Wald	**h1**	LT839092	Kindler *et al.* (2017)	northern group	—
**MTD T 14095**	Germany: North Rhine-Westphalia: near Dormagen: Knechtstedener Wald	**h1**	LT839092	Kindler *et al.* (2017)	northern group	—
**MTD T 14096**	Germany: North Rhine-Westphalia: near Dormagen: Knechtstedener Wald	**h1**	LT839092	Kindler *et al.* (2017)	northern group	—
**MTD T 14097**	Germany: North Rhine-Westphalia: near Dormagen: Knechtstedener Wald	**h1**	LT839092	Kindler *et al.* (2017)	northern group	—
**MTD T 14098**	Germany: North Rhine-Westphalia: near Dormagen: Knechtstedener Wald	**h1**	LT839092	Kindler *et al.* (2017)	northern group	—
**MTD T 14099**	Germany: North Rhine-Westphalia: near Dormagen: Knechtstedener Wald	**h1**	LT839092	Kindler *et al.* (2017)	northern group	—
**MTD T 14100**	Germany: North Rhine-Westphalia: near Dormagen: Knechtstedener Wald	**h1**	LT839092	Kindler *et al.* (2017)	northern group	—
**MTD T 14101**	Germany: North Rhine-Westphalia: near Dormagen: Knechtstedener Wald	**h1**	LT839092	Kindler *et al.* (2017)	northern group	—
**MTD T 14103**	Germany: North Rhine-Westphalia: near Dormagen: Knechtstedener Wald	**h1**	LT839092	Kindler *et al.* (2017)	northern group	—
**MTD T 14104**	Germany: North Rhine-Westphalia: near Dormagen: Knechtstedener Wald	**h1**	LT839092	Kindler *et al.* (2017)	northern group	—
**MTD T 14105**	Germany: North Rhine-Westphalia: near Dormagen: Knechtstedener Wald	**h1**	LT839092	Kindler *et al.* (2017)	northern group	—
**MTD T 14106**	Germany: North Rhine-Westphalia: near Dormagen: Knechtstedener Wald	**h1**	LT839092	Kindler *et al.* (2017)	northern group	—
**MTD T 14107**	Germany: North Rhine-Westphalia: near Dormagen: Knechtstedener Wald	**h1**	LT839092	Kindler *et al.* (2017)	northern group	—
**MTD T 14109**	Germany: North Rhine-Westphalia: near Dormagen: Knechtstedener Wald	**h1**	LT839092	Kindler *et al.* (2017)	northern group	—
**MTD T 14111**	Germany: North Rhine-Westphalia: near Dormagen: Knechtstedener Wald	**h1**	LT839092	Kindler *et al.* (2017)	northern group	—
**MTD T 14485**	Germany: North Rhine-Westphalia: near Olpe: Friesenhagen	**h1**	LT839092	Kindler *et al.* (2017)	northern group	—
**ZFMK 83771**	Germany: North Rhine-Westphalia: Nideggen/Eifel	**h1**	LT839092	Kindler *et al.* (2013)	northern group	—
**MTD T 13972**	Germany: North Rhine-Westphalia: Rösrath	**h1**	LT839092	Kindler *et al.* (2017)	northern group	—
**ZFMK 89086**	Germany: North Rhine-Westphalia: Sankt Augustin-Hangelar	**h1**	LT839092	Kindler *et al.* (2013)	northern group	—
**MTD T 11157**	Germany: North Rhine-Westphalia: Troisdorf	**h1**	LT839092	Kindler *et al.* (2017)	northern group	—
**MTD T 11996**	Germany: North Rhine-Westphalia: Troisdorf	**h1**	LT839092	Kindler *et al.* (2017)	northern group	—
**MTD T 14486**	Germany: North Rhine-Westphalia: Wesel	**h1**	LT839092	Kindler *et al.* (2017)	northern group	—
**MTD T 11795**	Germany: North Rhine-Westphalia: Wuppertal: Kohlfurt	**h1**	LT839092	Kindler *et al.* (2017)	northern group	—
**MTD T 11873**	Germany: North Rhine-Westphalia: Wuppertal: Kohlfurt	**h1**	LT839092	Kindler *et al.* (2017)	northern group	—
**MTD T 11877**	Germany: North Rhine-Westphalia: Wuppertal: Kohlfurt	**h1**	LT839092	Kindler *et al.* (2017)	northern group	—

Table S1 continued						
**Voucher**	**Locality**	**Haplotype**	**Accession number**	**Reference**	**Geographical division**	
					**Group**	**Subgroup**
**MTD T 11878**	Germany: North Rhine-Westphalia: Wuppertal: Kohlfurt	**h1**	LT839092	Kindler *et al.* (2017)	northern group	—
**MTD T 11880**	Germany: North Rhine-Westphalia: Wuppertal: Kohlfurt	**h1**	LT839092	Kindler *et al.* (2017)	northern group	—
**MTD T 11881**	Germany: North Rhine-Westphalia: Wuppertal: Kohlfurt	**h1**	LT839092	Kindler *et al.* (2017)	northern group	—
**MTD T 11883**	Germany: North Rhine-Westphalia: Wuppertal: Kohlfurt	**h1**	LT839092	Kindler *et al.* (2017)	northern group	—
**MTD T 11884**	Germany: North Rhine-Westphalia: Wuppertal: Kohlfurt	**h1**	LT839092	Kindler *et al.* (2017)	northern group	—
**MTD T 11886**	Germany: North Rhine-Westphalia: Wuppertal: Kohlfurt	**h1**	LT839092	Kindler *et al.* (2017)	northern group	—
**MTD T 11887**	Germany: North Rhine-Westphalia: Wuppertal: Kohlfurt	**h1**	LT839092	Kindler *et al.* (2017)	northern group	—
**MTD T 11904**	Germany: North Rhine-Westphalia: Wuppertal: Kohlfurt	**h1**	LT839092	Kindler *et al.* (2017)	northern group	—
**MTD T 11905**	Germany: North Rhine-Westphalia: Wuppertal: Kohlfurt	**h1**	LT839092	Kindler *et al.* (2017)	northern group	—
**MTD T 11907**	Germany: North Rhine-Westphalia: Wuppertal: Kohlfurt	**h1**	LT839092	Kindler *et al.* (2017)	northern group	—
**MTD T 12043**	Germany: North Rhine-Westphalia: Wuppertal: Kohlfurt	**h1**	LT839092	Kindler *et al.* (2017)	northern group	—
**MTD T 12045**	Germany: North Rhine-Westphalia: Wuppertal: Kohlfurt	**h1**	LT839092	Kindler *et al.* (2017)	northern group	—
**MTD T 12048**	Germany: North Rhine-Westphalia: Wuppertal: Kohlfurt	**h1**	LT839092	Kindler *et al.* (2017)	northern group	—
**MTD T 12049**	Germany: North Rhine-Westphalia: Wuppertal: Kohlfurt	**h1**	LT839092	Kindler *et al.* (2017)	northern group	—
**MTD T 12050**	Germany: North Rhine-Westphalia: Wuppertal: Kohlfurt	**h1**	LT839092	Kindler *et al.* (2017)	northern group	—
**MTD T 12051**	Germany: North Rhine-Westphalia: Wuppertal: Kohlfurt	**h1**	LT839092	Kindler *et al.* (2017)	northern group	—
**MTD T 12052**	Germany: North Rhine-Westphalia: Wuppertal: Kohlfurt	**h1**	LT839092	Kindler *et al.* (2017)	northern group	—
**MTD T 12053**	Germany: North Rhine-Westphalia: Wuppertal: Kohlfurt	**h1**	LT839092	Kindler *et al.* (2017)	northern group	—
**MTD T 12054**	Germany: North Rhine-Westphalia: Wuppertal: Kohlfurt	**h1**	LT839092	Kindler *et al.* (2017)	northern group	—
**ZFMK 73645**	Germany: North Rhine-Westphalia: Wuppertal: Morsbachtal	**h1**	LT839092	Kindler *et al.* (2013)	northern group	—
**MTD T 12057**	Germany: North Rhine-Westphalia: Wuppertal: Neu-Dornap	**h1**	LT839092	Kindler *et al.* (2017)	northern group	—
**ZFMK 73646**	Germany: North Rhine-Westphalia: Wuppertal: Vorwerk	**h1**	LT839092	Kindler *et al.* (2013)	northern group	—
**MTD T 14838**	Germany: Rhineland-Palatinate: Altenahr	**h5**	LT839096	Kindler *et al.* (2017)	northern group	—
**MTD T 12042**	Germany: Rhineland-Palatinate: between Oberpleis and Eudenbach	**h1**	LT839092	Kindler *et al.* (2017)	northern group	—
**SMF 56593**	Germany: Rhineland-Palatinate: Bürdenbach	**h1**	LT839092	Kindler *et al.* (2017)	northern group	—
**SMNS 14504**	Germany: Rhineland-Palatinate: Germersheim	**h1**	LT839092	Kindler *et al.* (2017)	northern group	—
**ZFMK 80876**	Germany: Rhineland-Palatinate: Horhausen	**h1**	LT839092	Kindler *et al.* (2013)	northern group	—
**MTD D 35933**	Germany: Rhineland-Palatinate: Lieser	**h1**	LT839092	Kindler *et al.* (2013)	northern group	—
**ZFMK 83703**	Germany: Rhineland-Palatinate: Maria Laach	**h1**	LT839092	Kindler *et al.* (2013)	northern group	—
**SMF 73299**	Germany: Rhineland-Palatinate: Nauroth	**h1**	LT839092	Kindler *et al.* (2017)	northern group	—
**MTD T 11573**	Germany: Rhineland-Palatinate: near Dreis	**h1**	LT839092	Kindler *et al.* (2017)	northern group	—
**MTD T 11758**	Germany: Rhineland-Palatinate: near Linz: St. Katharinen	**h1**	LT839092	Kindler *et al.* (2017)	northern group	—
**SMF 74940**	Germany: Rhineland-Palatinate: SW Koblenz: between Macken and Burgen	**h1**	LT839092	Kindler *et al.* (2017)	northern group	—
**ZFMK 80875**	Germany: Rhineland-Palatinate: Weltersburg	**h1**	LT839092	Kindler *et al.* (2013)	northern group	—
**MTD T 14169**	Great Britain	**h1**	LT839092	Kindler *et al.* (2017)	northern group	—
**MTD T 14807**	Great Britain: Brecklands area of Norfolk: near Thetford	**h1**	LT839092	Kindler *et al.* (2017)	northern group	—
**MTD T 14808**	Great Britain: Brecklands area of Norfolk: near Thetford	**h1**	LT839092	Kindler *et al.* (2017)	northern group	—
**MTD T 14809**	Great Britain: Brecklands area of Norfolk: near Thetford	**h1**	LT839092	Kindler *et al.* (2017)	northern group	—
**MTD T 14810**	Great Britain: Brecklands area of Norfolk: near Thetford	**h1**	LT839092	Kindler *et al.* (2017)	northern group	—
**MTD T 14811**	Great Britain: Brecklands area of Norfolk: near Thetford	**h1**	LT839092	Kindler *et al.* (2017)	northern group	—
**MTD T 14812**	Great Britain: Brecklands area of Norfolk: near Thetford	**h1**	LT839092	Kindler *et al.* (2017)	northern group	—
**MTD T 14130**	Great Britain: Buckingshamshire: Chesham	**h1**	LT839092	Kindler *et al.* (2017)	northern group	—
**MTD T 14144**	Great Britain: Denbighshire: Eryrys	**h1**	LT839092	Kindler *et al.* (2017)	northern group	—
**MTD T 14165**	Great Britain: Dorset	**h1**	LT839092	Kindler *et al.* (2017)	northern group	—

Table S1 continued						
**Voucher**	**Locality**	**Haplotype**	**Accession number**	**Reference**	**Geographical division**	
					**Group**	**Subgroup**
**MTD T 14166**	Great Britain: Dorset	**h1**	LT839092	Kindler *et al.* (2017)	northern group	—
**MTD T 14131**	Great Britain: Dorset: Bovington	**h6**	LT839097	Kindler *et al.* (2017)	northern group	—
**MTD T 14132**	Great Britain: Dorset: Christchurch	**h1**	LT839092	Kindler *et al.* (2017)	northern group	—
**MTD T 14117**	Great Britain: Essex: Loughton	**h1**	LT839092	Kindler *et al.* (2017)	northern group	—
**MTD T 14118**	Great Britain: Essex: Loughton	**h1**	LT839092	Kindler *et al.* (2017)	northern group	—
**MTD T 14119**	Great Britain: Essex: Loughton	**h1**	LT839092	Kindler *et al.* (2017)	northern group	—
**MTD T 14120**	Great Britain: Essex: Loughton	**h1**	LT839092	Kindler *et al.* (2017)	northern group	—
**MTD T 14121**	Great Britain: Essex: Loughton	**h1**	LT839092	Kindler *et al.* (2017)	northern group	—
**MTD T 14122**	Great Britain: Essex: Loughton	**h1**	LT839092	Kindler *et al.* (2017)	northern group	—
**MTD T 14136**	Great Britain: Gloucestershire: Milkwall	**h1**	LT839092	Kindler *et al.* (2017)	northern group	—
**MTD T 14124**	Great Britain: Hereford: 4km E Ledbury	**h1**	LT839092	Kindler *et al.* (2017)	northern group	—
**MTD T 14123**	Great Britain: Hereford: Ledbury	**h2**	LT839093	Kindler *et al.* (2017)	northern group	—
**MTD T 14139**	Great Britain: Herefordshire: Abbey Dore	**h1**	LT839092	Kindler *et al.* (2017)	northern group	—
**MTD T 14137**	Great Britain: Herefordshire: Tedstone Wafre	**h1**	LT839092	Kindler *et al.* (2017)	northern group	—
**MTD T 14167**	Great Britain: Hertfordshire: Hatfield	**h1**	LT839092	Kindler *et al.* (2017)	northern group	—
**MTD T 13012**	Great Britain: Hull	**h6**	LT839097	Kindler *et al.* (2017)	northern group	—
**MTD T 13011**	Great Britain: Hull	**h6**	LT839097	Kindler *et al.* (2017)	northern group	—
**MTD T 14133**	Great Britain: Kent: Hildenborough	**h1**	LT839092	Kindler *et al.* (2017)	northern group	—
**MTD T 14126**	Great Britain: near Scotland	**h1**	LT839092	Kindler *et al.* (2017)	northern group	—
**MTD T 14127**	Great Britain: near Scotland	**h1**	LT839092	Kindler *et al.* (2017)	northern group	—
**MTD T 14128**	Great Britain: near Scotland	**h1**	LT839092	Kindler *et al.* (2017)	northern group	—
**MTD T 14116**	Great Britain: Norfolk: Cirby Cane	**h1**	LT839092	Kindler *et al.* (2017)	northern group	—
**MTD T 9982**	Great Britain: North Wales: Gwaith Powder	**h1**	LT839092	Kindler *et al.* (2013)	northern group	—
**MTD T 9983**	Great Britain: North Wales: Gwaith Powder	**h1**	LT839092	Kindler *et al.* (2013)	northern group	—
**MTD T 14141**	Great Britain: Northhamptonshire: Sywell	**h1**	LT839092	Kindler *et al.* (2017)	northern group	—
**MTD T 14140**	Great Britain: Nottinghamshire: Jacksdale	**h1**	LT839092	Kindler *et al.* (2017)	northern group	—
**MTD T 14113**	Great Britain: Pembrokeshire: Talbenny	**h1**	LT839092	Kindler *et al.* (2017)	northern group	—
**MTD T 14142**	Great Britain: Staffordshire: between Wombourn and Sedgley	**h1**	LT839092	Kindler *et al.* (2017)	northern group	—
**MTD T 14163**	Great Britain: Suffolk: Martlesham	**h1**	LT839092	Kindler *et al.* (2017)	northern group	—
**MTD T 14164**	Great Britain: Suffolk: Martlesham	**h1**	LT839092	Kindler *et al.* (2017)	northern group	—
**MTD T 14134**	Great Britain: Surrey: Milford	**h1**	LT839092	Kindler *et al.* (2017)	northern group	—
**MTD T 14148**	Great Britain: Surrey: Newdigate	**h1**	LT839092	Kindler *et al.* (2017)	northern group	—
**MTD T 14149**	Great Britain: Surrey: Newdigate	**h1**	LT839092	Kindler *et al.* (2017)	northern group	—
**MTD T 14150**	Great Britain: Surrey: Newdigate	**h1**	LT839092	Kindler *et al.* (2017)	northern group	—
**MTD T 14154**	Great Britain: Surrey: Newdigate	**h1**	LT839092	Kindler *et al.* (2017)	northern group	—
**MTD T 14156**	Great Britain: Surrey: Newdigate	**h1**	LT839092	Kindler *et al.* (2017)	northern group	—
**MTD T 14158**	Great Britain: Surrey: Newdigate	**h1**	LT839092	Kindler *et al.* (2017)	northern group	—
**MTD T 14159**	Great Britain: Surrey: Newdigate	**h1**	LT839092	Kindler *et al.* (2017)	northern group	—
**MTD T 14160**	Great Britain: Surrey: Newdigate	**h1**	LT839092	Kindler *et al.* (2017)	northern group	—
**MTD T 14161**	Great Britain: Surrey: Newdigate	**h1**	LT839092	Kindler *et al.* (2017)	northern group	—
**MTD T 14464**	Great Britain: Surrey: Newdigate	**h1**	LT839092	Kindler *et al.* (2017)	northern group	—
**MTD T 14465**	Great Britain: Surrey: Newdigate	**h1**	LT839092	Kindler *et al.* (2017)	northern group	—
**MTD T 14468**	Great Britain: Surrey: Newdigate	**h1**	LT839092	Kindler *et al.* (2017)	northern group	—
**MTD T 14151**	Great Britain: Surrey: Newdigate	**h8**	LT839099	Kindler *et al.* (2017)	northern group	—

Table S1 continued						
**Voucher**	**Locality**	**Haplotype**	**Accession number**	**Reference**	**Geographical division**	
					**Group**	**Subgroup**
**MTD T 14155**	Great Britain: Surrey: Newdigate	**h8**	LT839099	Kindler *et al.* (2017)	northern group	—
**MTD T 14135**	Great Britain: Sussex: Heathfield	**h1**	LT839092	Kindler *et al.* (2017)	northern group	—
**MTD T 14143**	Great Britain: Warwickshire: Oxhill	**h1**	LT839092	Kindler *et al.* (2017)	northern group	—
**MTD T 14129**	Great Britain: Yorkshire: Bradford	**h1**	LT839092	Kindler *et al.* (2017)	northern group	—
**MTD T 14359**	Great Britain: Yorkshire: Skipwith Common	**h6**	LT839097	Kindler *et al.* (2017)	northern group	—
**MTD T 14360**	Great Britain: Yorkshire: Southern Washlands	**h6**	LT839097	Kindler *et al.* (2017)	northern group	—
**MTD T 14361**	Great Britain: Yorkshire: Southern Washlands	**h6**	LT839097	Kindler *et al.* (2017)	northern group	—
**MTD T 11572**	Italy: between Viozene and Ponte di Nava	**h1**	LT839092	This study	southern group	—
**MTD T 13957**	Netherlands: Amstelveen	**h1**	LT839092	Kindler *et al.* (2017)	northern group	—
**MTD T 13958**	Netherlands: Amstelveen	**h1**	LT839092	Kindler *et al.* (2017)	northern group	—
**MTD T 13959**	Netherlands: Amstelveen	**h1**	LT839092	Kindler *et al.* (2017)	northern group	—
**MTD T 12131**	Netherlands: Amsterdam: IJ	**h1**	LT839092	Kindler *et al.* (2017)	northern group	—
**MTD T 12132**	Netherlands: Amsterdam: IJ	**h1**	LT839092	Kindler *et al.* (2017)	northern group	—
**MTD T 13969**	Netherlands: Boschoord	**h1**	LT839092	Kindler *et al.* (2017)	northern group	—
**MTD T 13970**	Netherlands: Boschoord	**h1**	LT839092	Kindler *et al.* (2017)	northern group	—
**MTD T 13971**	Netherlands: Boschoord	**h1**	LT839092	Kindler *et al.* (2017)	northern group	—
**MTD T 13954**	Netherlands: Durgerdam V.E.	**h1**	LT839092	Kindler *et al.* (2017)	northern group	—
**MTD T 13955**	Netherlands: Durgerdam V.E.	**h1**	LT839092	Kindler *et al.* (2017)	northern group	—
**MTD T 13956**	Netherlands: Durgerdam V.E.	**h1**	LT839092	Kindler *et al.* (2017)	northern group	—
**MTD T 12129**	Netherlands: Flevoland: Kuinderbos	**h1**	LT839092	Kindler *et al.* (2017)	northern group	—
**MTD T 12133**	Netherlands: Flevoland: Kuinderbos	**h1**	LT839092	Kindler *et al.* (2017)	northern group	—
**MTD T 13960**	Netherlands: Hilversum	**h1**	LT839092	Kindler *et al.* (2017)	northern group	—
**MTD T 13961**	Netherlands: Hilversum	**h1**	LT839092	Kindler *et al.* (2017)	northern group	—
**MTD T 13962**	Netherlands: Hilversum	**h1**	LT839092	Kindler *et al.* (2017)	northern group	—
**MTD T 13942**	Netherlands: Houten	**h1**	LT839092	Kindler *et al.* (2017)	northern group	—
**MTD T 13943**	Netherlands: Houten	**h1**	LT839092	Kindler *et al.* (2017)	northern group	—
**MTD T 13944**	Netherlands: Houten	**h1**	LT839092	Kindler *et al.* (2017)	northern group	—
**MTD T 13939**	Netherlands: Leersum	**h1**	LT839092	Kindler *et al.* (2017)	northern group	—
**MTD T 13940**	Netherlands: Leersum	**h1**	LT839092	Kindler *et al.* (2017)	northern group	—
**MTD T 13941**	Netherlands: Leersum	**h1**	LT839092	Kindler *et al.* (2017)	northern group	—
**MTD T 13966**	Netherlands: Oldeberkoop	**h1**	LT839092	Kindler *et al.* (2017)	northern group	—
**MTD T 13967**	Netherlands: Oldeberkoop	**h1**	LT839092	Kindler *et al.* (2017)	northern group	—
**MTD T 13968**	Netherlands: Oldeberkoop	**h1**	LT839092	Kindler *et al.* (2017)	northern group	—
**MTD T 13945**	Netherlands: Oostvaardersplassen	**h1**	LT839092	Kindler *et al.* (2017)	northern group	—
**MTD T 13946**	Netherlands: Oostvaardersplassen	**h1**	LT839092	Kindler *et al.* (2017)	northern group	—
**MTD T 13947**	Netherlands: Oostvaardersplassen	**h1**	LT839092	Kindler *et al.* (2017)	northern group	—
**MTD T 13963**	Netherlands: Rimburg	**h1**	LT839092	Kindler *et al.* (2017)	northern group	—
**MTD T 13964**	Netherlands: Rimburg	**h1**	LT839092	Kindler *et al.* (2017)	northern group	—
**MTD T 13965**	Netherlands: Rimburg	**h1**	LT839092	Kindler *et al.* (2017)	northern group	—
**MTD T 13951**	Netherlands: Velp: Beekhuizen	**h1**	LT839092	Kindler *et al.* (2017)	northern group	—
**MTD T 13952**	Netherlands: Velp: Beekhuizen	**h1**	LT839092	Kindler *et al.* (2017)	northern group	—
**MTD T 13953**	Netherlands: Velp: Beekhuizen	**h1**	LT839092	Kindler *et al.* (2017)	northern group	—
**MTD T 13948**	Netherlands: Wageningen	**h1**	LT839092	Kindler *et al.* (2017)	northern group	—
**MTD T 13949**	Netherlands: Wageningen	**h1**	LT839092	Kindler *et al.* (2017)	northern group	—

Table S1 continued						
**Voucher**	**Locality**	**Haplotype**	**Accession number**	**Reference**	**Geographical division**	
					**Group**	**Subgroup**
**MTD T 13950**	Netherlands: Wageningen	**h1**	LT839092	Kindler *et al.* (2017)	northern group	—
**ROH01**	Switzerland: Aargau: Aarau: Rohr	**h1**	LT839092	Kindler *et al.* (2017)	northern group	—
**ROH04**	Switzerland: Aargau: Aarau: Rohr	**h1**	LT839092	Kindler *et al.* (2017)	northern group	—
**ROH02**	Switzerland: Aargau: Aarau: Rohr	**h9**	LT839100	Kindler *et al.* (2017)	northern group	—
**KGN01**	Switzerland: Aargau: Böttstein	**h1**	LT839092	Kindler *et al.* (2017)	northern group	—
**KGN02**	Switzerland: Aargau: Böttstein	**h1**	LT839092	Kindler *et al.* (2017)	northern group	—
**BRU01**	Switzerland: Aargau: Brugg	**h1**	LT839092	Kindler *et al.* (2017)	northern group	—
**BRU03**	Switzerland: Aargau: Brugg	**h1**	LT839092	Kindler *et al.* (2017)	northern group	—
**BRU02**	Switzerland: Aargau: Brugg	**h9**	LT839100	Kindler *et al.* (2017)	northern group	—
**AGO01**	Switzerland: Aargau: Eiken	**h9**	LT839100	Kindler *et al.* (2017)	northern group	—
**AGO02**	Switzerland: Aargau: Eiken	**h9**	LT839100	Kindler *et al.* (2017)	northern group	—
**AGO03**	Switzerland: Aargau: Eiken	**h9**	LT839100	Kindler *et al.* (2017)	northern group	—
**GIP01**	Switzerland: Aargau: Gippingen	**h1**	LT839092	Kindler *et al.* (2017)	northern group	—
**GIP05**	Switzerland: Aargau: Gippingen	**h1**	LT839092	Kindler *et al.* (2017)	northern group	—
**GIP02**	Switzerland: Aargau: Gippingen	**h9**	LT839100	Kindler *et al.* (2017)	northern group	—
**GIP03**	Switzerland: Aargau: Gippingen	**h9**	LT839100	Kindler *et al.* (2017)	northern group	—
**GIP04**	Switzerland: Aargau: Gippingen	**h9**	LT839100	Kindler *et al.* (2017)	northern group	—
**KLI01**	Switzerland: Aargau: Klingnau	**h1**	LT839092	Kindler *et al.* (2017)	northern group	—
**ROT01**	Switzerland: Aargau: Rottenschwil	**h1**	LT839092	Kindler *et al.* (2017)	northern group	—
**REG02**	Switzerland: Basel-Landschaft: Binningen	**h1**	LT839092	Kindler *et al.* (2017)	northern group	—
**MBL14**	Switzerland: Basel-Landschaft: Liestal	**h1**	LT839092	Kindler *et al.* (2017)	northern group	—
**REG01**	Switzerland: Basel-Landschaft: Pratteln	**h1**	LT839092	Kindler *et al.* (2017)	northern group	—
**BBE01**	Switzerland: Bern: Airport Bern	**h1**	LT839092	Kindler *et al.* (2017)	northern group	—
**BBE02**	Switzerland: Bern: Airport Bern	**h1**	LT839092	Kindler *et al.* (2017)	northern group	—
**BBE03**	Switzerland: Bern: Airport Bern	**h1**	LT839092	Kindler *et al.* (2017)	northern group	—
**BBE04**	Switzerland: Bern: Airport Bern	**h1**	LT839092	Kindler *et al.* (2017)	northern group	—
**BBE05**	Switzerland: Bern: Airport Bern	**h1**	LT839092	Kindler *et al.* (2017)	northern group	—
**BBE06**	Switzerland: Bern: Airport Bern	**h1**	LT839092	Kindler *et al.* (2017)	northern group	—
**MBE10**	Switzerland: Bern: Airport Bern	**h1**	LT839092	Kindler *et al.* (2017)	northern group	—
**MBE11**	Switzerland: Bern: Mühleberg	**h1**	LT839092	Kindler *et al.* (2017)	northern group	—
**MTD T 10083**	Switzerland: Bern: near Bern	**h1**	LT839092	Kindler *et al.* (2013)	northern group	—
**MTD T 10086**	Switzerland: Bern: near Meiringen: Gadmental	**h3**	LT839094	Kindler *et al.* (2013)	northern group	—
**MBE09**	Switzerland: Bern: Sutz-Lattrigen	**h1**	LT839092	Kindler *et al.* (2017)	northern group	—
**MTD T 10084**	Switzerland: Fribourg: between Neuchâtel, Bern and Biel: Grosses Moos	**h1**	LT839092	Kindler *et al.* (2013)	northern group	—
**MTD T 10095**	Switzerland: Fribourg: between Neuchâtel, Bern and Biel: Grosses Moos	**h1**	LT839092	Kindler *et al.* (2013)	northern group	—
**CHU01**	Switzerland: Graubünden: Chur	**h1**	LT839092	Kindler *et al.* (2017)	northern group	—
**BGR01**	Switzerland: Graubünden: Filisur	**h1**	LT839092	Kindler *et al.* (2017)	northern group	—
**FLA01**	Switzerland: Graubünden: Fläsch	**h1**	LT839092	Kindler *et al.* (2017)	northern group	—
**FLA02**	Switzerland: Graubünden: Fläsch	**h1**	LT839092	Kindler *et al.* (2017)	northern group	—
**FLA03**	Switzerland: Graubünden: Fläsch	**h1**	LT839092	Kindler *et al.* (2017)	northern group	—
**LAA01**	Switzerland: Graubünden: Laax	**h1**	LT839092	Kindler *et al.* (2017)	northern group	—
**LAA02**	Switzerland: Graubünden: Laax	**h1**	LT839092	Kindler *et al.* (2017)	northern group	—
**ALT01**	Switzerland: St. Gallen: Altstätten	**h1**	LT839092	Kindler *et al.* (2017)	northern group	—
**ALT02**	Switzerland: St. Gallen: Altstätten	**h1**	LT839092	Kindler *et al.* (2017)	northern group	—

Table S1 continued						
**Voucher**	**Locality**	**Haplotype**	**Accession number**	**Reference**	**Geographical division**	
					**Group**	**Subgroup**
**ALT03**	Switzerland: St. Gallen: Altstätten	**h1**	LT839092	Kindler *et al.* (2017)	northern group	—
**ALT04**	Switzerland: St. Gallen: Altstätten	**h1**	LT839092	Kindler *et al.* (2017)	northern group	—
**ALT05**	Switzerland: St. Gallen: Altstätten	**h1**	LT839092	Kindler *et al.* (2017)	northern group	—
**BAL01**	Switzerland: St. Gallen: Balgach	**h1**	LT839092	Kindler *et al.* (2017)	northern group	—
**BAL02**	Switzerland: St. Gallen: Balgach	**h1**	LT839092	Kindler *et al.* (2017)	northern group	—
**BAL04**	Switzerland: St. Gallen: Balgach	**h1**	LT839092	Kindler *et al.* (2017)	northern group	—
**BAL05**	Switzerland: St. Gallen: Balgach	**h1**	LT839092	Kindler *et al.* (2017)	northern group	—
**BCZ04**	Switzerland: St. Gallen: Berneck	**h1**	LT839092	Kindler *et al.* (2017)	northern group	—
**BCZ05**	Switzerland: St. Gallen: Berneck	**h1**	LT839092	Kindler *et al.* (2017)	northern group	—
**BCZ06**	Switzerland: St. Gallen: Berneck	**h1**	LT839092	Kindler *et al.* (2017)	northern group	—
**BCZ07**	Switzerland: St. Gallen: Berneck	**h1**	LT839092	Kindler *et al.* (2017)	northern group	—
**BCZ08**	Switzerland: St. Gallen: Berneck	**h1**	LT839092	Kindler *et al.* (2017)	northern group	—
**BCZ09**	Switzerland: St. Gallen: Berneck	**h1**	LT839092	Kindler *et al.* (2017)	northern group	—
**BCZ10**	Switzerland: St. Gallen: Berneck	**h1**	LT839092	Kindler *et al.* (2017)	northern group	—
**BCZ11**	Switzerland: St. Gallen: Berneck	**h1**	LT839092	Kindler *et al.* (2017)	northern group	—
**QUI01**	Switzerland: St. Gallen: Quinten	**h1**	LT839092	Kindler *et al.* (2017)	northern group	—
**SMN01**	Switzerland: St. Gallen: Schmerikon	**h1**	LT839092	Kindler *et al.* (2017)	northern group	—
**SMN02**	Switzerland: St. Gallen: Schmerikon	**h1**	LT839092	Kindler *et al.* (2017)	northern group	—
**SMN03**	Switzerland: St. Gallen: Schmerikon	**h1**	LT839092	Kindler *et al.* (2017)	northern group	—
**SEW01**	Switzerland: St. Gallen: Sennwald	**h1**	LT839092	Kindler *et al.* (2017)	northern group	—
**NEB02**	Switzerland: St. Gallen: Thal	**h1**	LT839092	Kindler *et al.* (2017)	northern group	—
**NEB05**	Switzerland: St. Gallen: Thal	**h1**	LT839092	Kindler *et al.* (2017)	northern group	—
**MUE01**	Switzerland: Uri: Erstfeld	**h1**	LT839092	Kindler *et al.* (2017)	northern group	—
**GEN01**	Switzerland: Vaud: Begnins	**h1**	LT839092	Kindler *et al.* (2017)	northern group	—
**MTD T 10079**	Switzerland: Vaud: Vinzel	**h1**	LT839092	Kindler *et al.* (2013)	northern group	—
**FIS01**	Switzerland: Zürich: Fischenthal	**h1**	LT839092	Kindler *et al.* (2017)	northern group	—
**FIS02**	Switzerland: Zürich: Fischenthal	**h1**	LT839092	Kindler *et al.* (2017)	northern group	—
**FIS03**	Switzerland: Zürich: Fischenthal	**h1**	LT839092	Kindler *et al.* (2017)	northern group	—
**GAT02**	Switzerland: Zürich: Gattikon	**h1**	LT839092	Kindler *et al.* (2017)	northern group	—
**GAT01**	Switzerland: Zürich: Gattikon	**h11**	LT839102	Kindler *et al.* (2017)	northern group	—
**PFA01**	Switzerland: Zürich: Lake Pfäffikon	**h1**	LT839092	Kindler *et al.* (2017)	northern group	—
**MUE07**	Switzerland: Zürich: Langnau am Albis	**h1**	LT839092	Kindler *et al.* (2017)	northern group	—
**OBF01**	Switzerland: Zürich: Obfelden	**h1**	LT839092	Kindler *et al.* (2017)	northern group	—
**OBF02**	Switzerland: Zürich: Obfelden	**h1**	LT839092	Kindler *et al.* (2017)	northern group	—
**STA01**	Switzerland: Zürich: Stäfa	**h1**	LT839092	Kindler *et al.* (2017)	northern group	—
**STA02**	Switzerland: Zürich: Stäfa	**h1**	LT839092	Kindler *et al.* (2017)	northern group	—
**STA03**	Switzerland: Zürich: Stäfa	**h1**	LT839092	Kindler *et al.* (2017)	northern group	—
**STA04**	Switzerland: Zürich: Stäfa	**h1**	LT839092	Kindler *et al.* (2017)	northern group	—
**GRF01**	Switzerland: Zürich: Uster	**h1**	LT839092	Kindler *et al.* (2017)	northern group	—
**WIN01**	Switzerland: Zürich: Winterthur: Sennhof	**h1**	LT839092	Kindler *et al.* (2017)	northern group	—
**WIN02**	Switzerland: Zürich: Winterthur: Sennhof	**h1**	LT839092	Kindler *et al.* (2017)	northern group	—
**WIN03**	Switzerland: Zürich: Winterthur: Sennhof	**h1**	LT839092	Kindler *et al.* (2017)	northern group	—
**WIN04**	Switzerland: Zürich: Winterthur: Sennhof	**h1**	LT839092	Kindler *et al.* (2017)	northern group	—
**ZEL01**	Switzerland: Zürich: Zell	**h1**	LT839092	Kindler *et al.* (2017)	northern group	—

Table S1 continued						
Voucher	Locality	Haplotype	Accession number	Reference	Geographical division	
					Group	Subgroup
**ZEL02**	Switzerland: Zürich: Zell	**h1**	LT839092	Kindler *et al.* (2017)	northern group	—
**ZEL03**	Switzerland: Zürich: Zell	**h1**	LT839092	Kindler *et al.* (2017)	northern group	—
**ZEL04**	Switzerland: Zürich: Zell	**h1**	LT839092	Kindler *et al.* (2017)	northern group	—
**ZEL05**	Switzerland: Zürich: Zell	**h1**	LT839092	Kindler *et al.* (2017)	northern group	—
**ZEL06**	Switzerland: Zürich: Zell	**h1**	LT839092	Kindler *et al.* (2017)	northern group	—
**ZEL07**	Switzerland: Zürich: Zell	**h1**	LT839092	Kindler *et al.* (2017)	northern group	—
**ZHC01**	Switzerland: Zürich: Zürich	**h1**	LT839092	Kindler *et al.* (2017)	northern group	—
**ZHC02**	Switzerland: Zürich: Zürich	**h1**	LT839092	Kindler *et al.* (2017)	northern group	—
**ZHN01**	Switzerland: Zürich: Zürich: Affoltern	**h1**	LT839092	Kindler *et al.* (2017)	northern group	—
**ZHN02**	Switzerland: Zürich: Zürich: Affoltern	**h1**	LT839092	Kindler *et al.* (2017)	northern group	—
**KLO04**	Switzerland: Zürich: Zürich: Airport	**h1**	LT839092	Kindler *et al.* (2017)	northern group	—
**KLO07**	Switzerland: Zürich: Zürich: Airport	**h1**	LT839092	Kindler *et al.* (2017)	northern group	—
**NHMW 38599**	Austria: Upper Austria: St. Ulrich bei Steyr	**y1**	LT839104	Kindler *et al.* (2017)	northern group	SCES
**NHMW 39122**	Austria: Upper Austria: St. Ulrich bei Steyr	**y1**	LT839104	Kindler *et al.* (2017)	northern group	SCES
**NHMW 40036 (1)**	Austria: Upper Austria: St. Ulrich bei Steyr	**y1**	LT839104	Kindler *et al.* (2017)	northern group	SCES
**MTD D 18924**	Bulgaria: Levunovo	**y15**	LT839118	Kindler *et al.* (2013)	southern group	—
**MTD D 18923**	Bulgaria: Levunovo	**y20**	LT839123	Kindler *et al.* (2013)	southern group	—
**MTD T 10940**	Bulgaria: Liljanovo	**y20**	LT839123	Kindler *et al.* (2017)	southern group	—
**BEV.T4054**	Bulgaria: Novo selo	**y15**	LT839118	Kindler *et al.* (2017)	southern group	—
**MTD T 9012**	Bulgaria: Pejo Javorov	**y18**	LT839121	Kindler *et al.* (2013)	southern group	—
**MTD T 9013**	Bulgaria: Pejo Javorov	**y20**	LT839123	Kindler *et al.* (2013)	southern group	—
**MTD D 19566**	Bulgaria: Sandanksi	**y20**	LT839123	Kindler *et al.* (2013)	southern group	—
**MTD T 12794**	Czech Republic: Stráž nad Ohří	**y1**	LT839104	Kindler *et al.* (2017)	northern group	SCES
**MTD T 9652**	Denmark: Funen: NNW Svendborg	**y1**	LT839104	Kindler *et al.* (2013)	northern group	NCESS
**MTD T 9653**	Denmark: Funen: NNW Svendborg	**y1**	LT839104	Kindler *et al.* (2013)	northern group	NCESS
**MTD T 9654**	Denmark: Funen: NNW Svendborg	**y1**	LT839104	Kindler *et al.* (2013)	northern group	NCESS
**MTD T 9655**	Denmark: Funen: NNW Svendborg	**y1**	LT839104	Kindler *et al.* (2013)	northern group	NCESS
**MTD T 9913**	Denmark: Jutland: SE Gammel Rye	**y1**	LT839104	Kindler *et al.* (2013)	northern group	NCESS
**MTD T 9914**	Denmark: Jutland: SE Gammel Rye	**y1**	LT839104	Kindler *et al.* (2013)	northern group	NCESS
**MTD T 9915**	Denmark: Jutland: SE Gammel Rye	**y1**	LT839104	Kindler *et al.* (2013)	northern group	NCESS
**MTD T 9916**	Denmark: Jutland: SE Skanderborg	**y1**	LT839104	Kindler *et al.* (2013)	northern group	NCESS
**MTD T 14559**	Denmark: Langeland: Hov	**y1**	LT839104	Kindler *et al.* (2017)	northern group	NCESS
**MTD T 9651**	Denmark: Langeland: S Tranekær	**y1**	LT839104	Kindler *et al.* (2013)	northern group	NCESS
**MTD T 10918**	Denmark: Zealand: 6 km W Koge	**y30**	LT839133	Kindler *et al.* (2014)	northern group	NCESS
**ZMH R09201**	Denmark: Zealand: Brønsholm	**y20**	LT839123	Kindler *et al.* (2017)	northern group	NCESS
**ZMH R09021**	Denmark: Zealand: Fredensborg	**y20**	LT839123	Kindler *et al.* (2017)	northern group	NCESS
**ZMH R09022**	Denmark: Zealand: Fredensborg	**y20**	LT839123	Kindler *et al.* (2017)	northern group	NCESS
**ZMH R09204**	Denmark: Zealand: Fredensborg	**y20**	LT839123	Kindler *et al.* (2017)	northern group	NCESS
**ZMH R09205**	Denmark: Zealand: Fredensborg	**y20**	LT839123	Kindler *et al.* (2017)	northern group	NCESS
**ZMH R09206**	Denmark: Zealand: Fredensborg	**y20**	LT839123	Kindler *et al.* (2017)	northern group	NCESS
**ZMH R09210**	Denmark: Zealand: Fredensborg	**y20**	LT839123	Kindler *et al.* (2017)	northern group	NCESS
**ZMH R09211**	Denmark: Zealand: Fredensborg	**y20**	LT839123	Kindler *et al.* (2017)	northern group	NCESS
**ZMH R09020**	Denmark: Zealand: Gribskov forest	**y20**	LT839123	Kindler *et al.* (2017)	northern group	NCESS
**ZMH R09030**	Denmark: Zealand: Gribskov forest	**y20**	LT839123	Kindler *et al.* (2017)	northern group	NCESS

Table S1 continued						
**Voucher**	**Locality**	**Haplotype**	**Accession number**	**Reference**	**Geographical division**	
					**Group**	**Subgroup**
**ZMH R09055**	Denmark: Zealand: Gribskov forest	**y20**	LT839123	Kindler *et al.* (2017)	northern group	NCESS
**MTD T 14546**	Denmark: Zealand: Holte: Vaserne	**y1**	LT839104	Kindler *et al.* (2017)	northern group	NCESS
**MTD T 14547**	Denmark: Zealand: Holte: Vaserne	**y1**	LT839104	Kindler *et al.* (2017)	northern group	NCESS
**MTD T 14548**	Denmark: Zealand: Holte: Vaserne	**y1**	LT839104	Kindler *et al.* (2017)	northern group	NCESS
**MTD T 14751**	Denmark: Zealand: N Copenhagen: Jægersborg Dyrehave	**y1**	LT839104	Kindler *et al.* (2017)	northern group	NCESS
**MTD T 9649**	Denmark: Zealand: N Præstø: Feddet	**y34**	LT839137	Kindler *et al.* (2013)	northern group	NCESS
**MTD T 9269**	Denmark: Zealand: S Borup	**y7**	LT839110	Kindler *et al.* (2013)	northern group	NCESS
**MTD T 9270**	Denmark: Zealand: S Borup	**y7**	LT839110	Kindler *et al.* (2013)	northern group	NCESS
**MTD T 9271**	Denmark: Zealand: S Borup	**y7**	LT839110	Kindler *et al.* (2013)	northern group	NCESS
**MTD T 14550**	Denmark: Zealand: S Hillerød: Børstingerød Mose	**y1**	LT839104	Kindler *et al.* (2017)	northern group	NCESS
**MTD T 14552**	Denmark: Zealand: S Hillerød: Børstingerød Mose	**y1**	LT839104	Kindler *et al.* (2017)	northern group	NCESS
**MTD T 14554**	Denmark: Zealand: S Hillerød: Børstingerød Mose	**y1**	LT839104	Kindler *et al.* (2017)	northern group	NCESS
**MTD T 14555**	Denmark: Zealand: S Hillerød: Børstingerød Mose	**y1**	LT839104	Kindler *et al.* (2017)	northern group	NCESS
**MTD T 14556**	Denmark: Zealand: S Hillerød: Børstingerød Mose	**y1**	LT839104	Kindler *et al.* (2017)	northern group	NCESS
**MTD T 14557**	Denmark: Zealand: S Hillerød: Børstingerød Mose	**y1**	LT839104	Kindler *et al.* (2017)	northern group	NCESS
**MTD T 14551**	Denmark: Zealand: S Hillerød: Børstingerød Mose	**y19**	LT839122	Kindler *et al.* (2017)	northern group	NCESS
**MTD T 14553**	Denmark: Zealand: S Hillerød: Børstingerød Mose	**y24**	LT839127	Kindler *et al.* (2017)	northern group	NCESS
**MTD T 14549**	Denmark: Zealand: S Hillerød: Brødeskov	**y1**	LT839104	Kindler *et al.* (2017)	northern group	NCESS
**MTD T 14558**	Denmark: Zealand: S Hillerød: near Lillerød: Ravnsholt	**y1**	LT839104	Kindler *et al.* (2017)	northern group	NCESS
**MTD T 14341**	Denmark: Zealand: W Køge	**y1**	LT839104	Kindler *et al.* (2017)	northern group	NCESS
**MTD T 14342**	Denmark: Zealand: W Køge	**y1**	LT839104	Kindler *et al.* (2017)	northern group	NCESS
**MTD T 9648**	Denmark: Zealand: W Køge	**y1**	LT839104	Kindler *et al.* (2013)	northern group	NCESS
**MTD T 11583**	Finland: Åland	**y1**	LT839104	Kindler *et al.* (2014)	northern group	NCESS
**MTD T 11584**	Finland: Åland	**y1**	LT839104	Kindler *et al.* (2014)	northern group	NCESS
**MTD T 11586**	Finland: Åland	**y5**	LT839108	Kindler *et al.* (2014)	northern group	NCESS
**MTD T 11589**	Finland: Åland: Finström	**y1**	LT839104	Kindler *et al.* (2014)	northern group	NCESS
**MTD T 11587**	Finland: Åland: Lemland: Bergö	**y5**	LT839108	Kindler *et al.* (2014)	northern group	NCESS
**MTD T 11588**	Finland: Åland: Lemland: Nåtö	**y1**	LT839104	Kindler *et al.* (2014)	northern group	NCESS
**MTD T 12985**	Former Yugoslav Republic of Macedonia: Crkvino	**y23**	LT839126	Kindler *et al.* (2017)	southern group	—
**MTD T 12986**	Former Yugoslav Republic of Macedonia: Dojran Lake: Mrdaja	**y15**	LT839118	Kindler *et al.* (2017)	southern group	—
**MTD T 12989**	Former Yugoslav Republic of Macedonia: Dojran Lake: Mrdaja	**y15**	LT839118	Kindler *et al.* (2017)	southern group	—
**MTD T 12987**	Former Yugoslav Republic of Macedonia: Dojran Lake: Mrdaja	**y17**	LT839120	Kindler *et al.* (2017)	southern group	—
**MTD T 12988**	Former Yugoslav Republic of Macedonia: Dojran Lake: Mrdaja	**y21**	LT839124	Kindler *et al.* (2017)	southern group	—
**BEV.T4055**	Former Yugoslav Republic of Macedonia: Jakubica Mountains	**y15**	LT839118	Kindler *et al.* (2017)	southern group	—
**MTD T 9911**	Former Yugoslav Republic of Macedonia: Jakubica Mountains	**y15**	LT839118	Kindler *et al.* (2013)	southern group	—
**MTD T 12993**	Former Yugoslav Republic of Macedonia: Lake Prespa: Pokrvenik	**y12**	LT839115	Kindler *et al.* (2017)	southern group	—
**MTD T 14447**	Former Yugoslav Republic of Macedonia: Nov Dojran	**y15**	LT839118	Kindler *et al.* (2017)	southern group	—
**MTD T 12990**	Former Yugoslav Republic of Macedonia: Star Dojran	**y15**	LT839118	Kindler *et al.* (2017)	southern group	—
**SMNS 14510**	Germany: Baden-Württemberg: Bad Waldsee	**y1**	LT839104	Kindler *et al.* (2017)	northern group	SCES
**SMNS 14508**	Germany: Baden-Württemberg: Bad Wurzach	**y1**	LT839104	Kindler *et al.* (2017)	northern group	SCES
**SMNS 14509**	Germany: Baden-Württemberg: Bad Wurzach	**y1**	LT839104	Kindler *et al.* (2017)	northern group	SCES
**MTD T 14814**	Germany: Baden-Württemberg: Berau	**y1**	LT839104	Kindler *et al.* (2017)	northern group	SCES
**MTD D 40907**	Germany: Baden-Württemberg: Böhringer See	**y1**	LT839104	Kindler *et al.* (2013)	northern group	SCES
**MTD T 14531**	Germany: Baden-Württemberg: Bronnbach	**y1**	LT839104	Kindler *et al.* (2017)	northern group	SCES

Table S1 continued						
**Voucher**	**Locality**	**Haplotype**	**Accession number**	**Reference**	**Geographical division**	
					**Group**	**Subgroup**
**MTD T 14816**	Germany: Baden-Württemberg: Freudenstadt	**y1**	LT839104	Kindler *et al.* (2017)	northern group	SCES
**MTD T 14835**	Germany: Baden-Württemberg: Freudenstadt	**y1**	LT839104	Kindler *et al.* (2017)	northern group	SCES
**MTD T 14858**	Germany: Baden-Württemberg: Gamburg	**y1**	LT839104	Kindler *et al.* (2017)	northern group	SCES
**SMNS 14506**	Germany: Baden-Württemberg: Herrenberg	**y1**	LT839104	Kindler *et al.* (2017)	northern group	SCES
**MTD T 14837**	Germany: Baden-Württemberg: Karlsruhe	**y1**	LT839104	Kindler *et al.* (2017)	northern group	SCES
**MTD T 14843**	Germany: Baden-Württemberg: Laufenburg-Grunholz	**y1**	LT839104	Kindler *et al.* (2017)	northern group	SCES
**SMNS 12851**	Germany: Baden-Württemberg: Ludwigsburg	**y1**	LT839104	Kindler *et al.* (2017)	northern group	SCES
**SMNS 12227**	Germany: Baden-Württemberg: Mettnau	**y37**	LT839140	Kindler *et al.* (2017)	northern group	SCES
**SMNS 14514**	Germany: Baden-Württemberg: near Gerlingen: Krummbachtal	**y1**	LT839104	Kindler *et al.* (2017)	northern group	SCES
**SMNS 14515**	Germany: Baden-Württemberg: near Gerlingen: Krummbachtal	**y1**	LT839104	Kindler *et al.* (2017)	northern group	SCES
**SMNS 14511**	Germany: Baden-Württemberg: near Heilbronn: Reichertshausen	**y1**	LT839104	Kindler *et al.* (2017)	northern group	SCES
**SMNS 9092**	Germany: Baden-Württemberg: near Ravensburg	**y1**	LT839104	Kindler *et al.* (2017)	northern group	SCES
**MTD T 14815**	Germany: Baden-Württemberg: Neckar valley	**y1**	LT839104	Kindler *et al.* (2017)	northern group	SCES
**MTD D 39068**	Germany: Baden-Württemberg: Oberweissach	**y1**	LT839104	Kindler *et al.* (2013)	northern group	SCES
**SMNS 14260**	Germany: Baden-Württemberg: Ohmden-Jesingen	**y1**	LT839104	Kindler *et al.* (2017)	northern group	SCES
**MTD T 14822**	Germany: Baden-Württemberg: Präg	**y1**	LT839104	Kindler *et al.* (2017)	northern group	SCES
**MTD T 14525**	Germany: Baden-Württemberg: Riedlingen	**y1**	LT839104	Kindler *et al.* (2017)	northern group	SCES
**MTD T 14526**	Germany: Baden-Württemberg: Riedlingen	**y1**	LT839104	Kindler *et al.* (2017)	northern group	SCES
**MTD T 14527**	Germany: Baden-Württemberg: Riedlingen	**y1**	LT839104	Kindler *et al.* (2017)	northern group	SCES
**MTD T 14528**	Germany: Baden-Württemberg: Riedlingen	**y1**	LT839104	Kindler *et al.* (2017)	northern group	SCES
**MTD T 14529**	Germany: Baden-Württemberg: Riedlingen	**y1**	LT839104	Kindler *et al.* (2017)	northern group	SCES
**MTD T 14829**	Germany: Baden-Württemberg: Schiltach	**y1**	LT839104	Kindler *et al.* (2017)	northern group	SCES
**MTD T 14831**	Germany: Baden-Württemberg: Schiltach	**y1**	LT839104	Kindler *et al.* (2017)	northern group	SCES
**MTD T 11994**	Germany: Baden-Württemberg: Schriesheim	**y1**	LT839104	Kindler *et al.* (2017)	northern group	SCES
**SMNS 14517**	Germany: Baden-Württemberg: Stuttgart-Botnang	**y1**	LT839104	Kindler *et al.* (2017)	northern group	SCES
**SMNS 14518**	Germany: Baden-Württemberg: Stuttgart-Feuerbach	**y1**	LT839104	Kindler *et al.* (2017)	northern group	SCES
**SMNS 14151**	Germany: Baden-Württemberg: Stuttgart-Hohenheim	**y1**	LT839104	Kindler *et al.* (2017)	northern group	SCES
**SMNS 14519**	Germany: Baden-Württemberg: Stuttgart-Hohenheim	**y1**	LT839104	Kindler *et al.* (2017)	northern group	SCES
**SMNS 14085**	Germany: Baden-Württemberg: Stuttgart-Vaihingen	**y1**	LT839104	Kindler *et al.* (2017)	northern group	SCES
**MTD T 14833**	Germany: Baden-Württemberg: Sulz-Fischingen	**y1**	LT839104	Kindler *et al.* (2017)	northern group	SCES
**MTD T 12038**	Germany: Baden-Württemberg: Tübingen: Wurmlingen	**y1**	LT839104	Kindler *et al.* (2017)	northern group	SCES
**MTD T 14834**	Germany: Baden-Württemberg: Zwiefalten	**y1**	LT839104	Kindler *et al.* (2017)	northern group	SCES
**MTD T 11137**	Germany: Bavaria: 1 km SSW Jachenau	**y1**	LT839104	Kindler *et al.* (2017)	northern group	SCES
**ZMH R09037**	Germany: Bavaria: Berchtesgaden	**y20**	LT839123	Kindler *et al.* (2017)	northern group	SCES
**MTD T 11141**	Germany: Bavaria: between Katschenreuth and Frankenberg	**y1**	LT839104	Kindler *et al.* (2017)	northern group	SCES
**MTD T 11142**	Germany: Bavaria: between Katschenreuth and Frankenberg	**y1**	LT839104	Kindler *et al.* (2017)	northern group	SCES
**MTD T 11143**	Germany: Bavaria: between Katschenreuth and Frankenberg	**y1**	LT839104	Kindler *et al.* (2017)	northern group	SCES
**MTD T 11145**	Germany: Bavaria: Dreschen	**y1**	LT839104	Kindler *et al.* (2017)	northern group	SCES
**MTD T 11147**	Germany: Bavaria: Haunstetten	**y1**	LT839104	Kindler *et al.* (2017)	northern group	SCES
**MTD T 11153**	Germany: Bavaria: Munich: Obermenzing	**y1**	LT839104	Kindler *et al.* (2017)	northern group	SCES
**MTD T 11154**	Germany: Bavaria: Munich: Obermenzing	**y1**	LT839104	Kindler *et al.* (2017)	northern group	SCES
**MTD T 11133**	Germany: Bavaria: Murnauer Moos	**y1**	LT839104	Kindler *et al.* (2017)	northern group	SCES
**MTD T 11144**	Germany: Bavaria: near Neuenreuth	**y1**	LT839104	Kindler *et al.* (2017)	northern group	SCES
**MTD T 14832**	Germany: Bavaria: Schwandorf	**y1**	LT839104	Kindler *et al.* (2017)	northern group	SCES

Table S1 continued						
**Voucher**	**Locality**	**Haplotype**	**Accession number**	**Reference**	**Geographical division**	
					**Group**	**Subgroup**
**MTD T 11140**	Germany: Bavaria: Wackersdorf	**y1**	LT839104	Kindler *et al.* (2017)	northern group	SCES
**MTD T 11148**	Germany: Bavaria: Wolfratshausen	**y1**	LT839104	Kindler *et al.* (2017)	northern group	SCES
**MTD T 14172**	Germany: Berlin: Teufelssee	**y1**	LT839104	Kindler *et al.* (2017)	northern group	NCESS
**MTD T 14173**	Germany: Berlin: Teufelssee	**y1**	LT839104	Kindler *et al.* (2017)	northern group	NCESS
**MTD D 47570**	Germany: Brandenburg: Brieske	**y1**	LT839104	Kindler *et al.* (2013)	northern group	NCESS
**NME R 0492/06**	Germany: Brandenburg: Casel	**y1**	LT839104	Kindler *et al.* (2017)	northern group	NCESS
**MTD T 11460**	Germany: Brandenburg: Forst	**y1**	LT839104	Kindler *et al.* (2017)	northern group	NCESS
**MTD T 14363**	Germany: Brandenburg: Golm	**y1**	LT839104	Kindler *et al.* (2017)	northern group	NCESS
**MTD T 14364**	Germany: Brandenburg: Golm	**y1**	LT839104	Kindler *et al.* (2017)	northern group	NCESS
**MTD T 14366**	Germany: Brandenburg: Golm	**y1**	LT839104	Kindler *et al.* (2017)	northern group	NCESS
**MTD T 14367**	Germany: Brandenburg: Golm	**y1**	LT839104	Kindler *et al.* (2017)	northern group	NCESS
**MTD T 14369**	Germany: Brandenburg: Golm	**y1**	LT839104	Kindler *et al.* (2017)	northern group	NCESS
**MTD T 14370**	Germany: Brandenburg: Golm	**y1**	LT839104	Kindler *et al.* (2017)	northern group	NCESS
**MTD T 14371**	Germany: Brandenburg: Golm	**y1**	LT839104	Kindler *et al.* (2017)	northern group	NCESS
**MTD T 14375**	Germany: Brandenburg: Golm	**y1**	LT839104	Kindler *et al.* (2017)	northern group	NCESS
**MTD T 14376**	Germany: Brandenburg: Golm	**y1**	LT839104	Kindler *et al.* (2017)	northern group	NCESS
**MTD T 14377**	Germany: Brandenburg: Golm	**y1**	LT839104	Kindler *et al.* (2017)	northern group	NCESS
**MTD T 14379**	Germany: Brandenburg: Golm	**y1**	LT839104	Kindler *et al.* (2017)	northern group	NCESS
**MTD T 14380**	Germany: Brandenburg: Golm	**y1**	LT839104	Kindler *et al.* (2017)	northern group	NCESS
**MTD T 14381**	Germany: Brandenburg: Golm	**y1**	LT839104	Kindler *et al.* (2017)	northern group	NCESS
**MTD T 14382**	Germany: Brandenburg: Golm	**y1**	LT839104	Kindler *et al.* (2017)	northern group	NCESS
**MTD T 14383**	Germany: Brandenburg: Golm	**y1**	LT839104	Kindler *et al.* (2017)	northern group	NCESS
**MTD T 14384**	Germany: Brandenburg: Golm	**y1**	LT839104	Kindler *et al.* (2017)	northern group	NCESS
**MTD T 14385**	Germany: Brandenburg: Golm	**y1**	LT839104	Kindler *et al.* (2017)	northern group	NCESS
**MTD T 14386**	Germany: Brandenburg: Golm	**y1**	LT839104	Kindler *et al.* (2017)	northern group	NCESS
**MTD T 14387**	Germany: Brandenburg: Golm	**y1**	LT839104	Kindler *et al.* (2017)	northern group	NCESS
**MTD T 14365**	Germany: Brandenburg: Golm	**y35**	LT839138	Kindler *et al.* (2017)	northern group	NCESS
**MTD T 14373**	Germany: Brandenburg: Golm	**y35**	LT839138	Kindler *et al.* (2017)	northern group	NCESS
**MTD T 14374**	Germany: Brandenburg: Golm	**y35**	LT839138	Kindler *et al.* (2017)	northern group	NCESS
**MTD T 14530**	Germany: Brandenburg: Gülpe	**y8**	LT839111	Kindler *et al.* (2017)	northern group	NCESS
**MTD D 47197**	Germany: Brandenburg: Guteborn	**y1**	LT839104	Kindler *et al.* (2013)	northern group	NCESS
**MTD D 45304**	Germany: Brandenburg: Hosena	**y1**	LT839104	Kindler *et al.* (2013)	northern group	NCESS
**ZMB 66570**	Germany: Brandenburg: near Bamme	**y1**	LT839104	Kindler *et al.* (2014)	northern group	NCESS
**MTD D 29504**	Germany: Brandenburg: Perleberg	**y20**	LT839123	Kindler *et al.* (2013)	northern group	NCESS
**MTD T 14345**	Germany: Brandenburg: Schwedt/Oder	**y1**	LT839104	Kindler *et al.* (2017)	northern group	NCESS
**ZFMK 76358**	Germany: Brandenburg: Senftenberg	**y1**	LT839104	Kindler *et al.* (2013)	northern group	NCESS
**MTD T 14368**	Germany: Brandenburg: Werder	**y13**	LT839116	Kindler *et al.* (2017)	northern group	NCESS
**ZMH R09735**	Germany: Hamburg: Klövensteen	**y1**	LT839104	Kindler *et al.* (2017)	northern group	NCESS
**ZMH R06162**	Germany: Hamburg: Ohlstedt	**y1**	LT839104	Kindler *et al.* (2017)	northern group	NCESS
**ZMH R09009**	Germany: Hamburg: Wohldorf	**y1**	LT839104	Kindler *et al.* (2017)	northern group	NCESS
**ZMH R09048**	Germany: Hamburg: Wohldorf	**y20**	LT839123	Kindler *et al.* (2017)	northern group	NCESS
**MTD T 14620**	Germany: Hesse: Bebra-Weiterode	**y1**	LT839104	Kindler *et al.* (2017)	northern group	NCESS
**SMF 71026**	Germany: Hesse: Biebergemünd	**y1**	LT839104	Kindler *et al.* (2017)	northern group	SCES
**SMF 71027**	Germany: Hesse: Biebergemünd	**y1**	LT839104	Kindler *et al.* (2017)	northern group	SCES

Table S1 continued						
**Voucher**	**Locality**	**Haplotype**	**Accession number**	**Reference**	**Geographical division**	
					**Group**	**Subgroup**
**SMF 77543**	Germany: Hesse: Biebergemünd-Breitenborn	**y1**	LT839104	Kindler *et al.* (2017)	northern group	SCES
**SMF 78480**	Germany: Hesse: Flieden	**y1**	LT839104	Kindler *et al.* (2017)	northern group	SCES
**ZFMK 82929**	Germany: Hesse: Flieden	**y1**	LT839104	Kindler *et al.* (2013)	northern group	SCES
**ZFMK 58023**	Germany: Hesse: Flieden	**y39**	LT839142	Kindler *et al.* (2017)	northern group	SCES
**SMF 79183**	Germany: Hesse: Hasselroth-Neuenhaßlau	**y1**	LT839104	Kindler *et al.* (2017)	northern group	SCES
**MTD T 14842**	Germany: Hesse: Hirschhorn	**y1**	LT839104	Kindler *et al.* (2017)	northern group	SCES
**MTD T 13929**	Germany: Hesse: Marburg: new botanical garden	**y1**	LT839104	Kindler *et al.* (2017)	northern group	NCESS
**MTD T 13930**	Germany: Hesse: Marburg: new botanical garden	**y1**	LT839104	Kindler *et al.* (2017)	northern group	NCESS
**MTD T 13931**	Germany: Hesse: Marburg: new botanical garden	**y39**	LT839142	Kindler *et al.* (2017)	northern group	NCESS
**MTD T 13932**	Germany: Hesse: Marburg: new botanical garden	**y39**	LT839142	Kindler *et al.* (2017)	northern group	NCESS
**MTD T 13933**	Germany: Hesse: Marburg: new botanical garden	**y39**	LT839142	Kindler *et al.* (2017)	northern group	NCESS
**MTD T 13400**	Germany: Hesse: near Bad Homburg	**y1**	LT839104	Kindler *et al.* (2017)	northern group	SCES
**SMF 73339**	Germany: Hesse: near Bieber	**y1**	LT839104	Kindler *et al.* (2017)	northern group	SCES
**SMF 74531**	Germany: Hesse: W Oberndorf	**y1**	LT839104	Kindler *et al.* (2017)	northern group	SCES
**MTD T 14749**	Germany: Lower Saxony: Cuxhaven: Spanger Heide	**y1**	LT839104	Kindler *et al.* (2017)	northern group	NCESS
**MTD T 14750**	Germany: Lower Saxony: Cuxhaven: Spanger Heide	**y1**	LT839104	Kindler *et al.* (2017)	northern group	NCESS
**LMNM REP963**	Germany: Lower Saxony: Frieschenmoor	**y1**	LT839104	Kindler *et al.* (2017)	northern group	NCESS
**MTD T 13919**	Germany: Lower Saxony: Gartow	**y35**	LT839138	Kindler *et al.* (2017)	northern group	NCESS
**MTD T 13367**	Germany: Lower Saxony: Hannover	**y1**	LT839104	Kindler *et al.* (2017)	northern group	NCESS
**MTD T 13374**	Germany: Lower Saxony: Hannover	**y1**	LT839104	Kindler *et al.* (2017)	northern group	NCESS
**LMNM REP961**	Germany: Lower Saxony: Hatterwüsting	**y1**	LT839104	Kindler *et al.* (2017)	northern group	NCESS
**LMNM REP107**	Germany: Lower Saxony: Hude	**y1**	LT839104	Kindler *et al.* (2017)	northern group	NCESS
**LMNM REP960**	Germany: Lower Saxony: Huntlosen	**y1**	LT839104	Kindler *et al.* (2017)	northern group	NCESS
**MTD T 13371**	Germany: Lower Saxony: Lachendorf	**y1**	LT839104	Kindler *et al.* (2017)	northern group	NCESS
**ZFMK 86134**	Germany: Lower Saxony: Lachendorf	**y1**	LT839104	Kindler *et al.* (2013)	northern group	NCESS
**MTD T 13370**	Germany: Lower Saxony: Lachendorf	**y35**	LT839138	Kindler *et al.* (2017)	northern group	NCESS
**ZFMK 89088**	Germany: Lower Saxony: Leiferde	**y1**	LT839104	Kindler *et al.* (2013)	northern group	NCESS
**ZMH R06756**	Germany: Lower Saxony: Lüchow-Dannenberg: Pevestorf	**y20**	LT839123	Kindler *et al.* (2017)	northern group	NCESS
**ZMH R06758**	Germany: Lower Saxony: Lüchow-Dannenberg: Pevestorf	**y20**	LT839123	Kindler *et al.* (2017)	northern group	NCESS
**ZMH R06945**	Germany: Lower Saxony: Lüchow-Dannenberg: Pevestorf	**y20**	LT839123	Kindler *et al.* (2017)	northern group	NCESS
**ZMH R06959**	Germany: Lower Saxony: Lüchow-Dannenberg: Pevestorf	**y20**	LT839123	Kindler *et al.* (2017)	northern group	NCESS
**ZMH R07057**	Germany: Lower Saxony: Lüchow-Dannenberg: Pevestorf	**y20**	LT839123	Kindler *et al.* (2017)	northern group	NCESS
**ZMH R07878**	Germany: Lower Saxony: Lüchow-Dannenberg: Pevestorf	**y20**	LT839123	Kindler *et al.* (2017)	northern group	NCESS
**ZMH R08415**	Germany: Lower Saxony: Lüchow-Dannenberg: Pevestorf	**y20**	LT839123	Kindler *et al.* (2017)	northern group	NCESS
**ZMH R08936**	Germany: Lower Saxony: Lüchow-Dannenberg: Pevestorf	**y20**	LT839123	Kindler *et al.* (2017)	northern group	NCESS
**ZMH R09335**	Germany: Lower Saxony: Lüchow-Dannenberg: Pevestorf	**y20**	LT839123	Kindler *et al.* (2017)	northern group	NCESS
**ZMH R09336**	Germany: Lower Saxony: Lüchow-Dannenberg: Pevestorf	**y20**	LT839123	Kindler *et al.* (2017)	northern group	NCESS
**ZMH R09337**	Germany: Lower Saxony: Lüchow-Dannenberg: Pevestorf	**y20**	LT839123	Kindler *et al.* (2017)	northern group	NCESS
**ZMH R09540**	Germany: Lower Saxony: Lüchow-Dannenberg: Pevestorf	**y20**	LT839123	Kindler *et al.* (2017)	northern group	NCESS
**ZMH R09636**	Germany: Lower Saxony: Lüchow-Dannenberg: Pevestorf	**y20**	LT839123	Kindler *et al.* (2017)	northern group	NCESS
**ZMH R09742**	Germany: Lower Saxony: Lüchow-Dannenberg: Pevestorf	**y20**	LT839123	Kindler *et al.* (2017)	northern group	NCESS
**ZMH R09754**	Germany: Lower Saxony: Lüchow-Dannenberg: Pevestorf	**y20**	LT839123	Kindler *et al.* (2017)	northern group	NCESS
**MTD T 13373**	Germany: Lower Saxony: Meißendorf	**y1**	LT839104	Kindler *et al.* (2017)	northern group	NCESS
**MTD T 13368**	Germany: Lower Saxony: near Celle: Bannetzer Moor	**y1**	LT839104	Kindler *et al.* (2017)	northern group	NCESS

Table S1 continued						
**Voucher**	**Locality**	**Haplotype**	**Accession number**	**Reference**	**Geographical division**	
					**Group**	**Subgroup**
**MTD T 13369**	Germany: Lower Saxony: near Cuxhaven: Arensch	**y1**	LT839104	Kindler *et al.* (2017)	northern group	NCESS
**MTD T 13372**	Germany: Lower Saxony: near Dannefeld	**y35**	LT839138	Kindler *et al.* (2017)	northern group	NCESS
**LMNM REP957**	Germany: Lower Saxony: Oldenbrok	**y1**	LT839104	Kindler *et al.* (2017)	northern group	NCESS
**LMNM REP496**	Germany: Lower Saxony: Oldenburg	**y1**	LT839104	Kindler *et al.* (2017)	northern group	NCESS
**LMNM REP962**	Germany: Lower Saxony: Oldenburg: Eversten	**y1**	LT839104	Kindler *et al.* (2017)	northern group	NCESS
**MTD T 14622**	Germany: Lower Saxony: Oldendorf (Luhe)	**y1**	LT839104	Kindler *et al.* (2017)	northern group	NCESS
**LMNM REP553**	Germany: Lower Saxony: Rastede	**y1**	LT839104	Kindler *et al.* (2017)	northern group	NCESS
**LMNM REP128**	Germany: Lower Saxony: S Oldenburg: Barneführer Holz	**y1**	LT839104	Kindler *et al.* (2017)	northern group	NCESS
**ZMH R06717**	Germany: Lower Saxony: Salzhausen	**y1**	LT839104	Kindler *et al.* (2017)	northern group	NCESS
**ZMH R09016**	Germany: Lower Saxony: Stade	**y1**	LT839104	Kindler *et al.* (2017)	northern group	NCESS
**MTD T 13917**	Germany: Lower Saxony: Steinhuder Meer	**y1**	LT839104	Kindler *et al.* (2017)	northern group	NCESS
**MTD T 13918**	Germany: Lower Saxony: Steinhuder Meer	**y1**	LT839104	Kindler *et al.* (2017)	northern group	NCESS
**MTD T 11465**	Germany: Mecklenburg-Western Pomerania: 3 km E Wesenberg	**y1**	LT839104	Kindler *et al.* (2014)	northern group	NCESS
**MTD T 11466**	Germany: Mecklenburg-Western Pomerania: 3 km E Wesenberg	**y1**	LT839104	Kindler *et al.* (2014)	northern group	NCESS
**MTD T 11467**	Germany: Mecklenburg-Western Pomerania: 3 km E Wesenberg	**y1**	LT839104	Kindler *et al.* (2014)	northern group	NCESS
**MTD T 11469**	Germany: Mecklenburg-Western Pomerania: 3 km E Wesenberg	**y1**	LT839104	Kindler *et al.* (2014)	northern group	NCESS
**MTD T 11470**	Germany: Mecklenburg-Western Pomerania: 3 km E Wesenberg	**y1**	LT839104	Kindler *et al.* (2014)	northern group	NCESS
**MTD T 11471**	Germany: Mecklenburg-Western Pomerania: 3 km E Wesenberg	**y1**	LT839104	Kindler *et al.* (2014)	northern group	NCESS
**MTD T 11472**	Germany: Mecklenburg-Western Pomerania: 3 km E Wesenberg	**y1**	LT839104	Kindler *et al.* (2014)	northern group	NCESS
**MTD T 11473**	Germany: Mecklenburg-Western Pomerania: 3 km E Wesenberg	**y1**	LT839104	Kindler *et al.* (2014)	northern group	NCESS
**MTD T 11474**	Germany: Mecklenburg-Western Pomerania: 3 km E Wesenberg	**y1**	LT839104	Kindler *et al.* (2014)	northern group	NCESS
**MTD T 11476**	Germany: Mecklenburg-Western Pomerania: 3 km E Wesenberg	**y1**	LT839104	Kindler *et al.* (2014)	northern group	NCESS
**MTD T 11477**	Germany: Mecklenburg-Western Pomerania: 3 km E Wesenberg	**y1**	LT839104	Kindler *et al.* (2014)	northern group	NCESS
**MTD T 11478**	Germany: Mecklenburg-Western Pomerania: 3 km E Wesenberg	**y1**	LT839104	Kindler *et al.* (2014)	northern group	NCESS
**MTD T 11479**	Germany: Mecklenburg-Western Pomerania: 3 km E Wesenberg	**y1**	LT839104	Kindler *et al.* (2014)	northern group	NCESS
**MTD T 11480**	Germany: Mecklenburg-Western Pomerania: 3 km E Wesenberg	**y1**	LT839104	Kindler *et al.* (2014)	northern group	NCESS
**MTD T 11481**	Germany: Mecklenburg-Western Pomerania: 3 km E Wesenberg	**y1**	LT839104	Kindler *et al.* (2014)	northern group	NCESS
**MTD T 11482**	Germany: Mecklenburg-Western Pomerania: 3 km E Wesenberg	**y1**	LT839104	Kindler *et al.* (2014)	northern group	NCESS
**MTD T 11468**	Germany: Mecklenburg-Western Pomerania: 3 km E Wesenberg	**y31**	LT839134	Kindler *et al.* (2014)	northern group	NCESS
**NME R 0567/08**	Germany: Mecklenburg-Western Pomerania: Boitin	**y1**	LT839104	Kindler *et al.* (2017)	northern group	NCESS
**NME R 0974/14**	Germany: Mecklenburg-Western Pomerania: Groß-Miltzow	**y1**	LT839104	Kindler *et al.* (2017)	northern group	NCESS
**NME R 0973/14**	Germany: Mecklenburg-Western Pomerania: Groß-Miltzow	**y22**	LT839125	Kindler *et al.* (2017)	northern group	NCESS
**NME R 0414/02**	Germany: Mecklenburg-Western Pomerania: near Ahlbeck	**y1**	LT839104	Kindler *et al.* (2017)	northern group	NCESS
**NME R 0963/14**	Germany: Mecklenburg-Western Pomerania: near Ahlbeck	**y1**	LT839104	Kindler *et al.* (2017)	northern group	NCESS
**MTD T 12145**	Germany: Mecklenburg-Western Pomerania: near Altwarp	**y1**	LT839104	Kindler *et al.* (2017)	northern group	NCESS
**MTD T 12146**	Germany: Mecklenburg-Western Pomerania: near Altwarp	**y1**	LT839104	Kindler *et al.* (2017)	northern group	NCESS
**MTD T 12147**	Germany: Mecklenburg-Western Pomerania: near Altwarp	**y1**	LT839104	Kindler *et al.* (2017)	northern group	NCESS
**MTD T 12148**	Germany: Mecklenburg-Western Pomerania: near Altwarp	**y1**	LT839104	Kindler *et al.* (2017)	northern group	NCESS
**MTD T 12149**	Germany: Mecklenburg-Western Pomerania: near Altwarp	**y1**	LT839104	Kindler *et al.* (2017)	northern group	NCESS
**MTD T 12150**	Germany: Mecklenburg-Western Pomerania: near Altwarp	**y1**	LT839104	Kindler *et al.* (2017)	northern group	NCESS
**MTD T 12135**	Germany: Mecklenburg-Western Pomerania: near Carwitz	**y1**	LT839104	Kindler *et al.* (2017)	northern group	NCESS
**MTD T 12136**	Germany: Mecklenburg-Western Pomerania: near Carwitz	**y1**	LT839104	Kindler *et al.* (2017)	northern group	NCESS
**MTD T 12137**	Germany: Mecklenburg-Western Pomerania: near Carwitz	**y1**	LT839104	Kindler *et al.* (2017)	northern group	NCESS
**MTD T 12139**	Germany: Mecklenburg-Western Pomerania: near Carwitz	**y1**	LT839104	Kindler *et al.* (2017)	northern group	NCESS

Table S1 continued						
**Voucher**	**Locality**	**Haplotype**	**Accession number**	**Reference**	**Geographical division**	
					**Group**	**Subgroup**
**MTD T 12140**	Germany: Mecklenburg-Western Pomerania: near Carwitz	**y1**	LT839104	Kindler *et al.* (2017)	northern group	NCESS
**MTD T 12142**	Germany: Mecklenburg-Western Pomerania: near Carwitz	**y1**	LT839104	Kindler *et al.* (2017)	northern group	NCESS
**MTD T 12144**	Germany: Mecklenburg-Western Pomerania: near Carwitz	**y1**	LT839104	Kindler *et al.* (2017)	northern group	NCESS
**MTD T 12134**	Germany: Mecklenburg-Western Pomerania: near Carwitz	**y22**	LT839125	Kindler *et al.* (2017)	northern group	NCESS
**MTD T 12138**	Germany: Mecklenburg-Western Pomerania: near Carwitz	**y22**	LT839125	Kindler *et al.* (2017)	northern group	NCESS
**MTD T 12141**	Germany: Mecklenburg-Western Pomerania: near Carwitz	**y22**	LT839125	Kindler *et al.* (2017)	northern group	NCESS
**MTD T 12143**	Germany: Mecklenburg-Western Pomerania: near Carwitz	**y22**	LT839125	Kindler *et al.* (2017)	northern group	NCESS
**MTD T 14482**	Germany: Mecklenburg-Western Pomerania: near Stralsund	**y1**	LT839104	Kindler *et al.* (2017)	northern group	NCESS
**MTD T 12829**	Germany: Mecklenburg-Western Pomerania: near Wustrow: Buchsee	**y1**	LT839104	Kindler *et al.* (2017)	northern group	NCESS
**MTD T 11462**	Germany: Mecklenburg-Western Pomerania: Neubrandenburg	**y1**	LT839104	Kindler *et al.* (2014)	northern group	NCESS
**MTD T 11463**	Germany: Mecklenburg-Western Pomerania: Neubrandenburg	**y1**	LT839104	Kindler *et al.* (2014)	northern group	NCESS
**MTD T 11464**	Germany: Mecklenburg-Western Pomerania: Neubrandenburg	**y1**	LT839104	Kindler *et al.* (2014)	northern group	NCESS
**NME R 0475/06**	Germany: Mecklenburg-Western Pomerania: Neubrandenburg	**y1**	LT839104	Kindler *et al.* (2017)	northern group	NCESS
**MTD T 11475**	Germany: Mecklenburg-Western Pomerania: Neubrandenburg	**y1**	LT839104	Kindler *et al.* (2014)	northern group	NCESS
**ZFMK 47435**	Germany: Mecklenburg-Western Pomerania: Neukloster	**y1**	LT839104	Kindler *et al.* (2013)	northern group	NCESS
**NME R 0474/06**	Germany: Mecklenburg-Western Pomerania: Prerow	**y43**	LT839146	Kindler *et al.* (2017)	northern group	NCESS
**MTD T 14508**	Germany: Mecklenburg-Western Pomerania: Rostock: Radelsee	**y10**	LT839113	Kindler *et al.* (2017)	northern group	NCESS
**MTD T 14483**	Germany: Mecklenburg-Western Pomerania: Saal: Neuendorf-Heide	**y1**	LT839104	Kindler *et al.* (2017)	northern group	NCESS
**MTD T 14484**	Germany: Mecklenburg-Western Pomerania: Saal: Neuendorf-Heide	**y1**	LT839104	Kindler *et al.* (2017)	northern group	NCESS
**MTD T 11483**	Germany: Mecklenburg-Western Pomerania: Usedom	**y1**	LT839104	Kindler *et al.* (2014)	northern group	NCESS
**MTD T 11484**	Germany: Mecklenburg-Western Pomerania: Usedom	**y1**	LT839104	Kindler *et al.* (2014)	northern group	NCESS
**MTD D 39712**	Germany: Saxony: Bad Düben	**y1**	LT839104	Kindler *et al.* (2013)	northern group	NCESS
**MTD T 10923**	Germany: Saxony: between Burkhartswalde and Obermunzig	**y1**	LT839104	Kindler *et al.* (2017)	northern group	NCESS
**MTD T 3183**	Germany: Saxony: Biehla	**y1**	LT839104	Kindler *et al.* (2013)	northern group	NCESS
**MWLK 192/13**	Germany: Saxony: Burkau	**y2**	LT839105	Kindler *et al.* (2017)	northern group	NCESS
**MTD D 47317**	Germany: Saxony: Chemnitz	**y9**	LT839112	Kindler *et al.* (2013)	northern group	NCESS
**MWLK 626/05**	Germany: Saxony: Cunnersdorf-Biehla	**y2**	LT839105	Kindler *et al.* (2013)	northern group	NCESS
**MTD D 46238**	Germany: Saxony: Dahlen	**y1**	LT839104	Kindler *et al.* (2013)	northern group	NCESS
**MWLK 588/11**	Germany: Saxony: Döbra	**y1**	LT839104	Kindler *et al.* (2017)	northern group	NCESS
**MTD D 47728**	Germany: Saxony: Dresden	**y1**	LT839104	Kindler *et al.* (2013)	northern group	NCESS
**MTD T 11539**	Germany: Saxony: Dresden	**y1**	LT839104	Kindler *et al.* (2017)	northern group	NCESS
**MTD T 13090**	Germany: Saxony: Dresden	**y1**	LT839104	Kindler *et al.* (2017)	northern group	NCESS
**MWLK 76/08**	Germany: Saxony: Dresden: Klotzsche	**y1**	LT839104	Kindler *et al.* (2017)	northern group	NCESS
**MTD T 12835**	Germany: Saxony: Dresden: Langebrück	**y1**	LT839104	Kindler *et al.* (2017)	northern group	NCESS
**MWLK 665/11**	Germany: Saxony: Dresden: Langebrück	**y1**	LT839104	Kindler *et al.* (2017)	northern group	NCESS
**MTD D 48756**	Germany: Saxony: Dresden: Weixdorf	**y1**	LT839104	Kindler *et al.* (2017)	northern group	NCESS
**MWLK 250/08**	Germany: Saxony: Droben	**y1**	LT839104	Kindler *et al.* (2017)	northern group	NCESS
**MWLK 399/01**	Germany: Saxony: Dubring-Scheckthal	**y1**	LT839104	Kindler *et al.* (2017)	northern group	NCESS
**MWLK 51/04**	Germany: Saxony: Dubringer Moor	**y1**	LT839104	Kindler *et al.* (2013)	northern group	NCESS
**MWLK 265/08**	Germany: Saxony: Groß Neida	**y1**	LT839104	Kindler *et al.* (2017)	northern group	NCESS
**MWLK 266/08**	Germany: Saxony: Groß Neida	**y1**	LT839104	Kindler *et al.* (2017)	northern group	NCESS
**MWLK 776/05**	Germany: Saxony: Guttau	**y1**	LT839104	Kindler *et al.* (2013)	northern group	NCESS
**MWLK 794/05**	Germany: Saxony: Jetscheba	**y1**	LT839104	Kindler *et al.* (2017)	northern group	NCESS
**MWLK 182/12**	Germany: Saxony: Kamenz	**y2**	LT839105	Kindler *et al.* (2017)	northern group	NCESS

Table S1 continued						
**Voucher**	**Locality**	**Haplotype**	**Accession number**	**Reference**	**Geographical division**	
					**Group**	**Subgroup**
**MWLK 988/05**	Germany: Saxony: Kamenz	**y2**	LT839105	Kindler *et al.* (2013)	northern group	NCESS
**MTD T 10926**	Germany: Saxony: Karsdorf	**y1**	LT839104	Kindler *et al.* (2017)	northern group	NCESS
**MTD T 10930**	Germany: Saxony: Karsdorf	**y1**	LT839104	Kindler *et al.* (2017)	northern group	NCESS
**MWLK 343/10**	Germany: Saxony: Liebegast	**y1**	LT839104	Kindler *et al.* (2017)	northern group	NCESS
**MTD T 12782**	Germany: Saxony: Liegau-Augustusbad	**y1**	LT839104	Kindler *et al.* (2017)	northern group	NCESS
**MTD T 13332**	Germany: Saxony: Liegau-Augustusbad	**y1**	LT839104	Kindler *et al.* (2017)	northern group	NCESS
**MTD T 10936**	Germany: Saxony: Medingen	**y1**	LT839104	Kindler *et al.* (2017)	northern group	NCESS
**MTD T 11106**	Germany: Saxony: Meißen	**y1**	LT839104	Kindler *et al.* (2017)	northern group	NCESS
**MTD T 11397**	Germany: Saxony: Meißen	**y1**	LT839104	Kindler *et al.* (2017)	northern group	NCESS
**MWLK 371/01**	Germany: Saxony: Michalken	**y1**	LT839104	Kindler *et al.* (2013)	northern group	NCESS
**MWLK 372/01**	Germany: Saxony: Michalken	**y1**	LT839104	Kindler *et al.* (2013)	northern group	NCESS
**MWLK 624/07**	Germany: Saxony: Michalken	**y1**	LT839104	Kindler *et al.* (2017)	northern group	NCESS
**MTD D 45006**	Germany: Saxony: Milkel	**y1**	LT839104	Kindler *et al.* (2013)	northern group	NCESS
**MWLK 793/05**	Germany: Saxony: Milkel	**y1**	LT839104	Kindler *et al.* (2017)	northern group	NCESS
**MWLK 1032/05**	Germany: Saxony: Mönau	**y1**	LT839104	Kindler *et al.* (2017)	northern group	NCESS
**MWLK 1049/05**	Germany: Saxony: Mönau	**y1**	LT839104	Kindler *et al.* (2017)	northern group	NCESS
**MTD T 10774**	Germany: Saxony: Moritzburg	**y1**	LT839104	Kindler *et al.* (2017)	northern group	NCESS
**MTD T 11395**	Germany: Saxony: near Dresden	**y1**	LT839104	Kindler *et al.* (2017)	northern group	NCESS
**MWLK 105/01**	Germany: Saxony: Neudorf	**y1**	LT839104	Kindler *et al.* (2017)	northern group	NCESS
**MWLK 311/05**	Germany: Saxony: Neukirch	**y1**	LT839104	Kindler *et al.* (2013)	northern group	NCESS
**MTD D 32501**	Germany: Saxony: Oppach	**y1**	LT839104	Kindler *et al.* (2013)	northern group	NCESS
**MWLK 141/09**	Germany: Saxony: Oppitz	**y1**	LT839104	Kindler *et al.* (2017)	northern group	NCESS
**MTD D 42679**	Germany: Saxony: Ottendorf-Okrilla	**y1**	LT839104	Kindler *et al.* (2013)	northern group	NCESS
**MTD T 11222**	Germany: Saxony: Ottendorf-Okrilla	**y1**	LT839104	Kindler *et al.* (2017)	northern group	NCESS
**MTD T 14857**	Germany: Saxony: Pirk: dam	**y9**	LT839112	Kindler *et al.* (2017)	northern group	NCESS
**MWLK 378/10**	Germany: Saxony: Prachenau	**y1**	LT839104	Kindler *et al.* (2017)	northern group	NCESS
**MWLK 284/99**	Germany: Saxony: Rehnsdorf	**y2**	LT839105	Kindler *et al.* (2013)	northern group	NCESS
**MTD T 13915**	Germany: Saxony: Rietschen	**y3**	LT839106	Kindler *et al.* (2017)	northern group	NCESS
**MTD T 13916**	Germany: Saxony: Rietschen	**y41**	LT839144	Kindler *et al.* (2017)	northern group	NCESS
**MWLK 68/02**	Germany: Saxony: Schönau	**y1**	LT839104	Kindler *et al.* (2017)	northern group	NCESS
**MWLK 1486/06**	Germany: Saxony: Schönbach-Bulleritz	**y2**	LT839105	Kindler *et al.* (2017)	northern group	NCESS
**MTD T 11030**	Germany: Saxony: Schönborn	**y1**	LT839104	Kindler *et al.* (2017)	northern group	NCESS
**MWLK 53/04**	Germany: Saxony: Schwepnitz	**y3**	LT839106	Kindler *et al.* (2013)	northern group	NCESS
**MWLK 137/12**	Germany: Saxony: Skaska	**y1**	LT839104	Kindler *et al.* (2017)	northern group	NCESS
**MWLK 204/12**	Germany: Saxony: Skaska	**y1**	LT839104	Kindler *et al.* (2017)	northern group	NCESS
**MWLK 687/11**	Germany: Saxony: Skaska	**y1**	LT839104	Kindler *et al.* (2017)	northern group	NCESS
**MWLK 537/11**	Germany: Saxony: Steinitz	**y1**	LT839104	Kindler *et al.* (2017)	northern group	NCESS
**MWLK 483/07**	Germany: Saxony: Straßgräbchen	**y2**	LT839105	Kindler *et al.* (2017)	northern group	NCESS
**MWLK 1047/05**	Germany: Saxony: Treugeböhla	**y14**	LT839117	Kindler *et al.* (2017)	northern group	NCESS
**MWLK 447/11**	Germany: Saxony: Uhyst-Mönau	**y1**	LT839104	Kindler *et al.* (2017)	northern group	NCESS
**MWLK 10/13**	Germany: Saxony: Uhyst-Mönau	**y32**	LT839135	Kindler *et al.* (2017)	northern group	NCESS
**MTD D 42680**	Germany: Saxony: Waldenburg	**y1**	LT839104	Kindler *et al.* (2013)	northern group	NCESS
**MWLK 1050/05**	Germany: Saxony: Wartha-Steinitz	**y26**	LT839129	Kindler *et al.* (2017)	northern group	NCESS
**MWLK 115/09**	Germany: Saxony: Weinböhla	**y1**	LT839104	Kindler *et al.* (2017)	northern group	NCESS

Table S1 continued						
**Voucher**	**Locality**	**Haplotype**	**Accession number**	**Reference**	**Geographical division**	
					**Group**	**Subgroup**
**MTD T 10775**	Germany: Saxony: Wermsdorf	**y1**	LT839104	Kindler *et al.* (2017)	northern group	NCESS
**MTD T 10994**	Germany: Saxony: Wermsdorf	**y1**	LT839104	Kindler *et al.* (2017)	northern group	NCESS
**MWLK 815/05**	Germany: Saxony: Wilthen	**y1**	LT839104	Kindler *et al.* (2017)	northern group	NCESS
**MWLK 144/01**	Germany: Saxony: Wittichenau	**y1**	LT839104	Kindler *et al.* (2013)	northern group	NCESS
**MTD D 41418**	Germany: Saxony-Anhalt: Altenbrak	**y1**	LT839104	Kindler *et al.* (2013)	northern group	NCESS
**MTD T 11578**	Germany: Saxony-Anhalt: near Klobikau: Lake Geißeltal	**y1**	LT839104	Kindler *et al.* (2017)	northern group	NCESS
**ZFMK 89087**	Germany: Saxony-Anhalt: Sangerhausen	**y1**	LT839104	Kindler *et al.* (2013)	northern group	NCESS
**MTD T 14072**	Germany: Schleswig-Holstein: Bad Bramstedt	**y1**	LT839104	Kindler *et al.* (2017)	northern group	NCESS
**ZMH R09486**	Germany: Schleswig-Holstein: Bargteheide	**y1**	LT839104	Kindler *et al.* (2017)	northern group	NCESS
**ZMH R09497**	Germany: Schleswig-Holstein: Bargteheide	**y1**	LT839104	Kindler *et al.* (2017)	northern group	NCESS
**ZMH R09855**	Germany: Schleswig-Holstein: Bargteheide	**y1**	LT839104	Kindler *et al.* (2017)	northern group	NCESS
**ZMH R11001**	Germany: Schleswig-Holstein: Bargteheide	**y1**	LT839104	Kindler *et al.* (2017)	northern group	NCESS
**MTD T 14846**	Germany: Schleswig-Holstein: Bark: Barker Heide	**y1**	LT839104	Kindler *et al.* (2017)	northern group	NCESS
**MTD T 14847**	Germany: Schleswig-Holstein: Dägeling: Heide Nordoe	**y1**	LT839104	Kindler *et al.* (2017)	northern group	NCESS
**MTD T 14070**	Germany: Schleswig-Holstein: Daldorf: Kiebitzholmer Moor	**y1**	LT839104	Kindler *et al.* (2017)	northern group	NCESS
**MTD T 14845**	Germany: Schleswig-Holstein: Dätgen	**y11**	LT839114	Kindler *et al.* (2017)	northern group	NCESS
**ZMH R09046**	Germany: Schleswig-Holstein: Dithmarschen	**y1**	LT839104	Kindler *et al.* (2017)	northern group	NCESS
**MTD T 14073**	Germany: Schleswig-Holstein: Felm: Kaltenhofer Moor	**y1**	LT839104	Kindler *et al.* (2017)	northern group	NCESS
**MTD T 14074**	Germany: Schleswig-Holstein: Felm: Kaltenhofer Moor	**y1**	LT839104	Kindler *et al.* (2017)	northern group	NCESS
**MTD T 14848**	Germany: Schleswig-Holstein: Felm: Kaltenhofer Moor	**y1**	LT839104	Kindler *et al.* (2017)	northern group	NCESS
**MTD T 14071**	Germany: Schleswig-Holstein: Gribbohm	**y11**	LT839114	Kindler *et al.* (2017)	northern group	NCESS
**MTD T 14069**	Germany: Schleswig-Holstein: Hamdorf	**y1**	LT839104	Kindler *et al.* (2017)	northern group	NCESS
**MTD T 14066**	Germany: Schleswig-Holstein: Hamdorf	**y11**	LT839114	Kindler *et al.* (2017)	northern group	NCESS
**ZFMK 85184**	Germany: Schleswig-Holstein: Kiel: Elmschenhagen	**y1**	LT839104	Kindler *et al.* (2013)	northern group	NCESS
**ZFMK 62405**	Germany: Schleswig-Holstein: Kiel: Landwehr	**y1**	LT839104	Kindler *et al.* (2013)	northern group	NCESS
**MTD T 13934**	Germany: Schleswig-Holstein: Klein Rönnau	**y1**	LT839104	Kindler *et al.* (2017)	northern group	NCESS
**ZMH R09338**	Germany: Schleswig-Holstein: Langenhorst	**y1**	LT839104	Kindler *et al.* (2017)	northern group	NCESS
**MTD T 14068**	Germany: Schleswig-Holstein: Lübeck: Grönauer Heide	**y1**	LT839104	Kindler *et al.* (2017)	northern group	NCESS
**MTD T 13935**	Germany: Schleswig-Holstein: Lübeck: Schellbruch	**y1**	LT839104	Kindler *et al.* (2017)	northern group	NCESS
**MTD T 13936**	Germany: Schleswig-Holstein: Lübeck: Schellbruch	**y1**	LT839104	Kindler *et al.* (2017)	northern group	NCESS
**MTD T 14063**	Germany: Schleswig-Holstein: Osterrönfeld	**y1**	LT839104	Kindler *et al.* (2017)	northern group	NCESS
**ZFMK 92535**	Germany: Schleswig-Holstein: Probstei: Hagener Moor	**y1**	LT839104	Kindler *et al.* (2013)	northern group	NCESS
**ZFMK 73639**	Germany: Schleswig-Holstein: Probsteierhagen	**y1**	LT839104	Kindler *et al.* (2013)	northern group	NCESS
**ZMH R04381**	Germany: Schleswig-Holstein: Rendsburg-Eckernförde: Warder	**y1**	LT839104	Kindler *et al.* (2017)	northern group	NCESS
**MTD T 14064**	Germany: Schleswig-Holstein: Sehestedt	**y1**	LT839104	Kindler *et al.* (2017)	northern group	NCESS
**NME R 0409/02**	Germany: Thuringia: Cospeda	**y1**	LT839104	Kindler *et al.* (2017)	northern group	NCESS
**NME R 0445/04**	Germany: Thuringia: Erfurt: Rhoda	**y1**	LT839104	Kindler *et al.* (2017)	northern group	NCESS
**NME R 0507/07**	Germany: Thuringia: Erfurt: Rhoda	**y1**	LT839104	Kindler *et al.* (2017)	northern group	NCESS
**NME R 0556/08**	Germany: Thuringia: Erfurt: Rhoda	**y1**	LT839104	Kindler *et al.* (2017)	northern group	NCESS
**NME R 0696/11**	Germany: Thuringia: Geroda	**y1**	LT839104	Kindler *et al.* (2017)	northern group	SCES
**NME R 0793/13**	Germany: Thuringia: Großheringen	**y1**	LT839104	Kindler *et al.* (2017)	northern group	NCESS
**NME R 0956/14**	Germany: Thuringia: Heroldsdorf	**y1**	LT839104	Kindler *et al.* (2017)	northern group	NCESS
**NME R 0415/02**	Germany: Thuringia: Rotheul	**y1**	LT839104	Kindler *et al.* (2017)	northern group	SCES
**MTD T 12792**	Germany: Thuringia: Schömbach dam	**y1**	LT839104	Kindler *et al.* (2017)	northern group	NCESS

Table S1 continued						
**Voucher**	**Locality**	**Haplotype**	**Accession number**	**Reference**	**Geographical division**	
					**Group**	**Subgroup**
**NME R 0253/99**	Germany: Thuringia: Triebes	**y1**	LT839104	Kindler *et al.* (2017)	northern group	NCESS
**NME R 0676/10**	Germany: Thuringia: Triebes	**y1**	LT839104	Kindler *et al.* (2017)	northern group	NCESS
**NME R 0495/06**	Germany: Thuringia: Uhlstädt-Kirchhasel	**y1**	LT839104	Kindler *et al.* (2017)	northern group	NCESS
**NME R 0890/13**	Germany: Thuringia: Wandersleben	**y1**	LT839104	Kindler *et al.* (2017)	northern group	NCESS
**NME R 0413/02**	Germany: Thuringia: Zeulenroda-Triebes	**y9**	LT839112	Kindler *et al.* (2017)	northern group	NCESS
**ZFMK 69946**	Greece: Chalkidiki: N Stratoni	**y15**	LT839118	Kindler *et al.* (2013)	southern group	—
**ZFMK 62945**	Greece: Chalkidiki: N Stratoni	**y16**	LT839119	Kindler *et al.* (2013)	southern group	—
**ZFMK 85407**	Greece: Cyclades: Milos	**y15**	LT839118	Kindler *et al.* (2013)	southern group	—
**MTD T 14327**	Greece: Milos	**y15**	LT839118	Kindler *et al.* (2017)	southern group	—
**MTD T 9907**	Greece: Skiros	**y15**	LT839118	Kindler *et al.* (2013)	southern group	—
**MTD D 29269**	Greece: Volos	**y15**	LT839118	Kindler *et al.* (2013)	southern group	—
**ZFMK 62956**	Greece: Xanthus: Avdira	**y15**	LT839118	Kindler *et al.* (2013)	southern group	—
**ZFMK 92957**	Greece: Xanthus: Avdira	**y15**	LT839118	Kindler *et al.* (2013)	southern group	—
**MTD T 14461**	Kosovo: Topojan	**y21**	LT839124	Kindler *et al.* (2017)	southern group	—
**MTD T 11568**	Norway: Akershus: Ski	**y1**	LT839104	Kindler *et al.* (2014)	northern group	NCESS
**MTD T 11569**	Norway: Akershus: Ski	**y1**	LT839104	Kindler *et al.* (2014)	northern group	NCESS
**ZMUO 54-96**	Norway: Akershus: Son	**y1**	LT839104	Kindler *et al.* (2013)	northern group	NCESS
**MTD T 11567**	Norway: Andebu: Ilestadvannet	**y1**	LT839104	Kindler *et al.* (2014)	northern group	NCESS
**MTD T 11566**	Norway: Holmestrand: Hallingsrud	**y1**	LT839104	Kindler *et al.* (2014)	northern group	NCESS
**ZMUO 31-83**	Norway: Oslo: Grønmo: Sølvdobla	**y1**	LT839104	Kindler *et al.* (2013)	northern group	NCESS
**ZMUO 26-85**	Norway: Oslo: Maridalen: Skjervensaga	**y1**	LT839104	Kindler *et al.* (2013)	northern group	NCESS
**MTD T 11571**	Norway: Østfold: Moss	**y1**	LT839104	Kindler *et al.* (2014)	northern group	NCESS
**MTD T 11570**	Norway: southern Oslo	**y1**	LT839104	Kindler *et al.* (2014)	northern group	NCESS
**ZMUO IH 10/2000**	Norway: Telemark: Levangshalvøya	**y1**	LT839104	Kindler *et al.* (2013)	northern group	NCESS
**MTD T 10026**	Poland: Upper Silesia: Kotórz Wielki	**y1**	LT839104	Kindler *et al.* (2013)	northern group	NCESS
**MTD T 11485**	Poland: Usedom	**y1**	LT839104	Kindler *et al.* (2014)	northern group	NCESS
**MTD T 11486**	Poland: Usedom	**y1**	LT839104	Kindler *et al.* (2014)	northern group	NCESS
**MTD T 12984**	Serbia: Prohor	**y15**	LT839118	Kindler *et al.* (2017)	southern group	—
**MTD T 8632**	Slovakia: Kraľovany: Kraľovanská dolina	**y1**	LT839104	Kindler *et al.* (2013)	northern group	SCES
**MTD T 11594**	Slovakia: Ružomberok	**y1**	LT839104	Kindler *et al.* (2017)	northern group	SCES
**MTD T 9015**	Slovakia: Tatra Mountains: Žiar	**y1**	LT839104	Kindler *et al.* (2013)	northern group	SCES
**ZMH R09056**	Sweden: Blekinge: Augerum	**y1**	LT839104	Kindler *et al.* (2017)	northern group	NCESS
**ZFMK 36114**	Sweden: Gotland	**y1**	LT839104	Kindler *et al.* (2013)	northern group	NCESS
**MTD T 11582**	Sweden: Närke: Klockhammar	**y1**	LT839104	Kindler *et al.* (2014)	northern group	NCESS
**ZMH R09209**	Sweden: Öland: between Stenasa and Resmo: Mockelmossen	**y1**	LT839104	Kindler *et al.* (2017)	northern group	NCESS
**MTD T 10921**	Sweden: Öland: Halltorps Hage	**y1**	LT839104	Kindler *et al.* (2014)	northern group	NCESS
**MTD T 10922**	Sweden: Öland: Halltorps Hage	**y1**	LT839104	Kindler *et al.* (2014)	northern group	NCESS
**MTD T 10920**	Sweden: Öland: Halltorps Hage	**y36**	LT839139	Kindler *et al.* (2014)	northern group	NCESS
**ZMH R09024**	Sweden: Skåne: Maglehem	**y20**	LT839123	Kindler *et al.* (2017)	northern group	NCESS
**ZMH R09026**	Sweden: Skåne: Olseröd	**y1**	LT839104	Kindler *et al.* (2017)	northern group	NCESS
**MTD T 10919**	Sweden: Småland: Kråksmåla	**y25**	LT839128	Kindler *et al.* (2014)	northern group	NCESS
**MTD T 11580**	Sweden: Södermanland: near Boo	**y1**	LT839104	Kindler *et al.* (2014)	northern group	NCESS
**MTD T 11579**	Sweden: Södermanland: near Nyköping	**y1**	LT839104	Kindler *et al.* (2014)	northern group	NCESS
**MTD T 11581**	Sweden: Södermanland: near Nyköping	**y1**	LT839104	Kindler *et al.* (2014)	northern group	NCESS

Table S1 continued						
**Voucher**	**Locality**	**Haplotype**	**Accession number**	**Reference**	**Geographical division**	
					**Group**	**Subgroup**
**BEV.6417**	Sweden: Uppland: Forsmark	**y1**	LT839104	Kindler *et al.* (2017)	northern group	NCESS
**BEV.6419**	Sweden: Uppland: near Uppsala: Östervåla	**y4**	LT839107	Kindler *et al.* (2017)	northern group	NCESS
**BEV.6420**	Sweden: Uppland: near Uppsala: Östervåla	**y6**	LT839109	Kindler *et al.* (2017)	northern group	NCESS
**MTD T 14859**	Sweden: Västergötland: Rånnaväg	**y1**	LT839104	Kindler *et al.* (2017)	northern group	NCESS
**MTD T 14860**	Sweden: Västergötland: Vegby	**y1**	LT839104	Kindler *et al.* (2017)	northern group	NCESS
**BAD01**	Switzerland: Aargau: Bad Zurzach	**y1**	LT839104	Kindler *et al.* (2017)	northern group	SCES
**BAD02**	Switzerland: Aargau: Bad Zurzach	**y1**	LT839104	Kindler *et al.* (2017)	northern group	SCES
**BAD03**	Switzerland: Aargau: Bad Zurzach	**y1**	LT839104	Kindler *et al.* (2017)	northern group	SCES
**BAD04**	Switzerland: Aargau: Bad Zurzach	**y1**	LT839104	Kindler *et al.* (2017)	northern group	SCES
**BAD05**	Switzerland: Aargau: Bad Zurzach	**y1**	LT839104	Kindler *et al.* (2017)	northern group	SCES
**BAD06**	Switzerland: Aargau: Bad Zurzach	**y1**	LT839104	Kindler *et al.* (2017)	northern group	SCES
**BAD07**	Switzerland: Aargau: Bad Zurzach	**y1**	LT839104	Kindler *et al.* (2017)	northern group	SCES
**BAD08**	Switzerland: Aargau: Bad Zurzach	**y1**	LT839104	Kindler *et al.* (2017)	northern group	SCES
**BAD09**	Switzerland: Aargau: Bad Zurzach	**y1**	LT839104	Kindler *et al.* (2017)	northern group	SCES
**BAD10**	Switzerland: Aargau: Bad Zurzach	**y1**	LT839104	Kindler *et al.* (2017)	northern group	SCES
**RIM01**	Switzerland: Aargau: Rietheim	**y37**	LT839140	Kindler *et al.* (2017)	northern group	SCES
**SHO01**	Switzerland: Schaffhausen: Hallau	**y1**	LT839104	Kindler *et al.* (2017)	northern group	SCES
**SHO02**	Switzerland: Schaffhausen: Hallau	**y1**	LT839104	Kindler *et al.* (2017)	northern group	SCES
**SHO03**	Switzerland: Schaffhausen: Hallau	**y1**	LT839104	Kindler *et al.* (2017)	northern group	SCES
**SAR01**	Switzerland: Schaffhausen: Hemishofen	**y1**	LT839104	Kindler *et al.* (2017)	northern group	SCES
**SAR03**	Switzerland: Schaffhausen: Hemishofen	**y1**	LT839104	Kindler *et al.* (2017)	northern group	SCES
**SAR02**	Switzerland: Schaffhausen: Hemishofen	**y37**	LT839140	Kindler *et al.* (2017)	northern group	SCES
**SHF01**	Switzerland: Schaffhausen: Herblingen	**y1**	LT839104	Kindler *et al.* (2017)	northern group	SCES
**SHF02**	Switzerland: Schaffhausen: Herblingen	**y1**	LT839104	Kindler *et al.* (2017)	northern group	SCES
**SHF03**	Switzerland: Schaffhausen: Herblingen	**y1**	LT839104	Kindler *et al.* (2017)	northern group	SCES
**MTD D 30567**	Switzerland: St. Gallen: Altenrhein	**y1**	LT839104	Kindler *et al.* (2013)	northern group	SCES
**BAL03**	Switzerland: St. Gallen: Balgach	**y40**	LT839143	Kindler *et al.* (2017)	northern group	SCES
**BCZ02**	Switzerland: St. Gallen: Berneck	**y1**	LT839104	Kindler *et al.* (2017)	northern group	SCES
**BCZ03**	Switzerland: St. Gallen: Berneck	**y1**	LT839104	Kindler *et al.* (2017)	northern group	SCES
**DIE03**	Switzerland: St. Gallen: Diepoldsau	**y1**	LT839104	Kindler *et al.* (2017)	northern group	SCES
**DIE01**	Switzerland: St. Gallen: Diepoldsau	**y40**	LT839143	Kindler *et al.* (2017)	northern group	SCES
**DIE02**	Switzerland: St. Gallen: Diepoldsau	**y40**	LT839143	Kindler *et al.* (2017)	northern group	SCES
**MTD T 10088**	Switzerland: St. Gallen: Lake Constance: near Thal	**y1**	LT839104	Kindler *et al.* (2013)	northern group	SCES
**OBE01**	Switzerland: St. Gallen: Oberuzwil	**y1**	LT839104	Kindler *et al.* (2017)	northern group	SCES
**OBE02**	Switzerland: St. Gallen: Oberuzwil	**y1**	LT839104	Kindler *et al.* (2017)	northern group	SCES
**OBE05**	Switzerland: St. Gallen: Oberuzwil	**y1**	LT839104	Kindler *et al.* (2017)	northern group	SCES
**OBE06**	Switzerland: St. Gallen: Oberuzwil	**y1**	LT839104	Kindler *et al.* (2017)	northern group	SCES
**OBE03**	Switzerland: St. Gallen: Oberuzwil	**y42**	LT839145	Kindler *et al.* (2017)	northern group	SCES
**OBE04**	Switzerland: St. Gallen: Oberuzwil	**y42**	LT839145	Kindler *et al.* (2017)	northern group	SCES
**OBE07**	Switzerland: St. Gallen: Oberuzwil	**y42**	LT839145	Kindler *et al.* (2017)	northern group	SCES
**NEB03**	Switzerland: St. Gallen: Thal	**y1**	LT839104	Kindler *et al.* (2017)	northern group	SCES
**NEB04**	Switzerland: St. Gallen: Thal	**y1**	LT839104	Kindler *et al.* (2017)	northern group	SCES
**THA01**	Switzerland: St. Gallen: Thal	**y1**	LT839104	Kindler *et al.* (2017)	northern group	SCES
**THA03**	Switzerland: St. Gallen: Thal	**y1**	LT839104	Kindler *et al.* (2017)	northern group	SCES

Table S1 continued						
**Voucher**	**Locality**	**Haplotype**	**Accession number**	**Reference**	**Geographical division**	
					**Group**	**Subgroup**
**NEB01**	Switzerland: St. Gallen: Thal	**y40**	LT839143	Kindler *et al.* (2017)	northern group	SCES
**THA02**	Switzerland: St. Gallen: Thal	**y40**	LT839143	Kindler *et al.* (2017)	northern group	SCES
**WIL01**	Switzerland: St. Gallen: Will	**y1**	LT839104	Kindler *et al.* (2017)	northern group	SCES
**AAD01**	Switzerland: Thurgau: Aadorf	**y37**	LT839140	Kindler *et al.* (2017)	northern group	SCES
**AAD02**	Switzerland: Thurgau: Aadorf	**y37**	LT839140	Kindler *et al.* (2017)	northern group	SCES
**AAD03**	Switzerland: Thurgau: Aadorf	**y37**	LT839140	Kindler *et al.* (2017)	northern group	SCES
**JUV04**	Switzerland: Thurgau: Alterswilen	**y1**	LT839104	Kindler *et al.* (2017)	northern group	SCES
**NWN01**	Switzerland: Thurgau: Alterswilen	**y1**	LT839104	Kindler *et al.* (2017)	northern group	SCES
**NWN02**	Switzerland: Thurgau: Alterswilen	**y1**	LT839104	Kindler *et al.* (2017)	northern group	SCES
**JUV03**	Switzerland: Thurgau: Alterswilen	**y27**	LT839130	Kindler *et al.* (2017)	northern group	SCES
**JUV05**	Switzerland: Thurgau: Alterswilen	**y27**	LT839130	Kindler *et al.* (2017)	northern group	SCES
**JUV06**	Switzerland: Thurgau: Alterswilen	**y27**	LT839130	Kindler *et al.* (2017)	northern group	SCES
**JUV01**	Switzerland: Thurgau: Alterswilen	**y28**	LT839131	Kindler *et al.* (2017)	northern group	SCES
**JUV02**	Switzerland: Thurgau: Alterswilen	**y29**	LT839132	Kindler *et al.* (2017)	northern group	SCES
**BUR01**	Switzerland: Thurgau: Bürglen	**y1**	LT839104	Kindler *et al.* (2017)	northern group	SCES
**BUR02**	Switzerland: Thurgau: Bürglen	**y1**	LT839104	Kindler *et al.* (2017)	northern group	SCES
**FRA01**	Switzerland: Thurgau: Frauenfeld	**y1**	LT839104	Kindler *et al.* (2017)	northern group	SCES
**FRA02**	Switzerland: Thurgau: Frauenfeld	**y37**	LT839140	Kindler *et al.* (2017)	northern group	SCES
**KRE01**	Switzerland: Thurgau: Kreuzlingen	**y1**	LT839104	Kindler *et al.* (2017)	northern group	SCES
**KRE02**	Switzerland: Thurgau: Kreuzlingen	**y1**	LT839104	Kindler *et al.* (2017)	northern group	SCES
**KRE03**	Switzerland: Thurgau: Kreuzlingen	**y1**	LT839104	Kindler *et al.* (2017)	northern group	SCES
**DAT01**	Switzerland: Zürich: Dätwil	**y37**	LT839140	Kindler *et al.* (2017)	northern group	SCES
**DAT02**	Switzerland: Zürich: Dätwil	**y37**	LT839140	Kindler *et al.* (2017)	northern group	SCES
**DUB01**	Switzerland: Zürich: Dübendorf	**y1**	LT839104	Kindler *et al.* (2017)	northern group	SCES
**DUB02**	Switzerland: Zürich: Dübendorf	**y1**	LT839104	Kindler *et al.* (2017)	northern group	SCES
**DUB03**	Switzerland: Zürich: Dübendorf	**y1**	LT839104	Kindler *et al.* (2017)	northern group	SCES
**DUB04**	Switzerland: Zürich: Dübendorf	**y1**	LT839104	Kindler *et al.* (2017)	northern group	SCES
**FCH02**	Switzerland: Zürich: Flaach	**y1**	LT839104	Kindler *et al.* (2017)	northern group	SCES
**FCH04**	Switzerland: Zürich: Flaach	**y1**	LT839104	Kindler *et al.* (2017)	northern group	SCES
**FCH06**	Switzerland: Zürich: Flaach	**y1**	LT839104	Kindler *et al.* (2017)	northern group	SCES
**FCH01**	Switzerland: Zürich: Flaach	**y37**	LT839140	Kindler *et al.* (2017)	northern group	SCES
**FCH03**	Switzerland: Zürich: Flaach	**y37**	LT839140	Kindler *et al.* (2017)	northern group	SCES
**MAR02**	Switzerland: Zürich: Marthalen	**y1**	LT839104	Kindler *et al.* (2017)	northern group	SCES
**MAR01**	Switzerland: Zürich: Marthalen	**y38**	LT839141	Kindler *et al.* (2017)	northern group	SCES
**MAR03**	Switzerland: Zürich: Marthalen	**y38**	LT839141	Kindler *et al.* (2017)	northern group	SCES
**NEE01**	Switzerland: Zürich: Neerach	**y1**	LT839104	Kindler *et al.* (2017)	northern group	SCES
**NEE02**	Switzerland: Zürich: Neerach	**y1**	LT839104	Kindler *et al.* (2017)	northern group	SCES
**NEE03**	Switzerland: Zürich: Neerach	**y1**	LT839104	Kindler *et al.* (2017)	northern group	SCES
**NEE04**	Switzerland: Zürich: Neerach	**y1**	LT839104	Kindler *et al.* (2017)	northern group	SCES
**PFU02**	Switzerland: Zürich: Pfungen	**y1**	LT839104	Kindler *et al.* (2017)	northern group	SCES
**PFU03**	Switzerland: Zürich: Pfungen	**y1**	LT839104	Kindler *et al.* (2017)	northern group	SCES
**PFU01**	Switzerland: Zürich: Pfungen	**y33**	LT839136	Kindler *et al.* (2017)	northern group	SCES
**TAT01**	Switzerland: Zürich: Thalheim an der Thur	**y1**	LT839104	Kindler *et al.* (2017)	northern group	SCES
**WEI01**	Switzerland: Zürich: Weiach	**y1**	LT839104	Kindler *et al.* (2017)	northern group	SCES

Table S1 continued						
**Voucher**	**Locality**	**Haplotype**	**Accession number**	**Reference**	**Geographical division**	
					**Group**	**Subgroup**
**WEI02**	Switzerland: Zürich: Weiach	**y1**	LT839104	Kindler *et al.* (2017)	northern group	SCES
**WEI03**	Switzerland: Zürich: Weiach	**y33**	LT839136	Kindler *et al.* (2017)	northern group	SCES
**KLO01**	Switzerland: Zürich: Zürich: Airport	**y1**	LT839104	Kindler *et al.* (2017)	northern group	SCES
**KLO02**	Switzerland: Zürich: Zürich: Airport	**y1**	LT839104	Kindler *et al.* (2017)	northern group	SCES
**KLO03**	Switzerland: Zürich: Zürich: Airport	**y1**	LT839104	Kindler *et al.* (2017)	northern group	SCES
**KLO05**	Switzerland: Zürich: Zürich: Airport	**y1**	LT839104	Kindler *et al.* (2017)	northern group	SCES
**KLO06**	Switzerland: Zürich: Zürich: Airport	**y1**	LT839104	Kindler *et al.* (2017)	northern group	SCES
**KLO08**	Switzerland: Zürich: Zürich: Airport	**y1**	LT839104	Kindler *et al.* (2017)	northern group	SCES
**KLO09**	Switzerland: Zürich: Zürich: Airport	**y1**	LT839104	Kindler *et al.* (2017)	northern group	SCES
**KLO10**	Switzerland: Zürich: Zürich: Airport	**y1**	LT839104	Kindler *et al.* (2017)	northern group	SCES
**ZFMK 65105**	Austria: Burgenland: Andau	**r30**	LT839176	Kindler *et al.* (2013)	southern group	Carpathian Basin
**NHMW 39266 (2)**	Austria: Burgenland: Apetlon	**r3**	LT839149	Kindler *et al.* (2017)	southern group	Carpathian Basin
**ZFMK 68656**	Austria: Burgenland: Apetlon	**r4**	LT839150	Kindler *et al.* (2013)	southern group	Carpathian Basin
**ZFMK 55995**	Austria: Burgenland: Breitenbrunn	**r4**	LT839150	Kindler *et al.* (2013)	southern group	Carpathian Basin
**NHMW 39030 (2)**	Austria: Burgenland: Eisenstadt	**r4**	LT839150	Kindler *et al.* (2017)	southern group	Carpathian Basin
**ZFMK 74926**	Austria: Burgenland: Illmitz	**r4**	LT839150	Kindler *et al.* (2013)	southern group	Carpathian Basin
**ZFMK 65687**	Austria: Burgenland: Jois	**r3**	LT839149	Kindler *et al.* (2013)	southern group	Carpathian Basin
**NHMW 38644**	Austria: Burgenland: Lake Neusiedl	**r3**	LT839149	Kindler *et al.* (2017)	southern group	Carpathian Basin
**MTD T 11991**	Austria: Burgenland: Lake Neusiedl: Illmitz	**r4**	LT839150	Kindler *et al.* (2017)	southern group	Carpathian Basin
**MTD T 12062**	Austria: Burgenland: Lake Neusiedl: Illmitz	**r4**	LT839150	Kindler *et al.* (2017)	southern group	Carpathian Basin
**MTD T 12063**	Austria: Burgenland: Lake Neusiedl: Illmitz	**r4**	LT839150	Kindler *et al.* (2017)	southern group	Carpathian Basin
**NHMW 38809**	Austria: Burgenland: Lake Neusiedl: Illmitz	**r4**	LT839150	Kindler *et al.* (2017)	southern group	Carpathian Basin
**NHMW 39436 (2)**	Austria: Burgenland: Lake Neusiedl: Illmitz	**r4**	LT839150	Kindler *et al.* (2017)	southern group	Carpathian Basin
**NHMW 40024 (1)**	Austria: Burgenland: Lake Neusiedl: Illmitz	**r4**	LT839150	Kindler *et al.* (2017)	southern group	Carpathian Basin
**MTD T 12068**	Austria: Burgenland: Lake Neusiedl: Jois	**r13**	LT839159	Kindler *et al.* (2017)	southern group	Carpathian Basin
**MTD T 12067**	Austria: Burgenland: Lake Neusiedl: Jois	**r3**	LT839149	Kindler *et al.* (2017)	southern group	Carpathian Basin
**MTD T 12077**	Austria: Burgenland: Lake Neusiedl: Jois	**r3**	LT839149	Kindler *et al.* (2017)	southern group	Carpathian Basin
**MTD T 11988**	Austria: Burgenland: Lake Neusiedl: Jois	**r30**	LT839176	Kindler *et al.* (2017)	southern group	Carpathian Basin
**MTD T 12076**	Austria: Burgenland: Lake Neusiedl: Jois	**r30**	LT839176	Kindler *et al.* (2017)	southern group	Carpathian Basin
**MTD T 11986**	Austria: Burgenland: Lake Neusiedl: Jois	**r4**	LT839150	Kindler *et al.* (2017)	southern group	Carpathian Basin
**MTD T 11987**	Austria: Burgenland: Lake Neusiedl: Jois	**r4**	LT839150	Kindler *et al.* (2017)	southern group	Carpathian Basin
**MTD T 11989**	Austria: Burgenland: Lake Neusiedl: Jois	**r4**	LT839150	Kindler *et al.* (2017)	southern group	Carpathian Basin
**MTD T 11990**	Austria: Burgenland: Lake Neusiedl: Jois	**r4**	LT839150	Kindler *et al.* (2017)	southern group	Carpathian Basin
**MTD T 11992**	Austria: Burgenland: Lake Neusiedl: Jois	**r4**	LT839150	Kindler *et al.* (2017)	southern group	Carpathian Basin
**MTD T 12058**	Austria: Burgenland: Lake Neusiedl: Jois	**r4**	LT839150	Kindler *et al.* (2017)	southern group	Carpathian Basin
**MTD T 12059**	Austria: Burgenland: Lake Neusiedl: Jois	**r4**	LT839150	Kindler *et al.* (2017)	southern group	Carpathian Basin
**MTD T 12060**	Austria: Burgenland: Lake Neusiedl: Jois	**r4**	LT839150	Kindler *et al.* (2017)	southern group	Carpathian Basin
**MTD T 12061**	Austria: Burgenland: Lake Neusiedl: Jois	**r4**	LT839150	Kindler *et al.* (2017)	southern group	Carpathian Basin
**MTD T 12064**	Austria: Burgenland: Lake Neusiedl: Jois	**r4**	LT839150	Kindler *et al.* (2017)	southern group	Carpathian Basin
**MTD T 12065**	Austria: Burgenland: Lake Neusiedl: Jois	**r4**	LT839150	Kindler *et al.* (2017)	southern group	Carpathian Basin
**MTD T 12066**	Austria: Burgenland: Lake Neusiedl: Jois	**r4**	LT839150	Kindler *et al.* (2017)	southern group	Carpathian Basin
**MTD T 12069**	Austria: Burgenland: Lake Neusiedl: Jois	**r4**	LT839150	Kindler *et al.* (2017)	southern group	Carpathian Basin
**MTD T 12070**	Austria: Burgenland: Lake Neusiedl: Jois	**r4**	LT839150	Kindler *et al.* (2017)	southern group	Carpathian Basin
**MTD T 12071**	Austria: Burgenland: Lake Neusiedl: Jois	**r4**	LT839150	Kindler *et al.* (2017)	southern group	Carpathian Basin

Table S1 continued						
**Voucher**	**Locality**	**Haplotype**	**Accession number**	**Reference**	**Geographical division**	
					**Group**	**Subgroup**
**MTD T 12072**	Austria: Burgenland: Lake Neusiedl: Jois	**r4**	LT839150	Kindler *et al.* (2017)	southern group	Carpathian Basin
**MTD T 12073**	Austria: Burgenland: Lake Neusiedl: Jois	**r4**	LT839150	Kindler *et al.* (2017)	southern group	Carpathian Basin
**MTD T 12074**	Austria: Burgenland: Lake Neusiedl: Jois	**r4**	LT839150	Kindler *et al.* (2017)	southern group	Carpathian Basin
**MTD T 12075**	Austria: Burgenland: Lake Neusiedl: Jois	**r4**	LT839150	Kindler *et al.* (2017)	southern group	Carpathian Basin
**MTD T 12078**	Austria: Burgenland: Lake Neusiedl: Jois	**r4**	LT839150	Kindler *et al.* (2017)	southern group	Carpathian Basin
**NHMW 39266 (1)**	Austria: Burgenland: Lake Neusiedl: Neusiedl am See	**r4**	LT839150	Kindler *et al.* (2017)	southern group	Carpathian Basin
**ZFMK 51670**	Austria: Burgenland: Weiden am See	**r10**	LT839156	Kindler *et al.* (2013)	southern group	Carpathian Basin
**ZFMK 91242**	Austria: Burgenland: Zitzmannsdorfer Wiesen	**r4**	LT839150	Kindler *et al.* (2013)	southern group	Carpathian Basin
**MTD T 11964**	Austria: Carinthia: Lake Faak	**r4**	LT839150	Kindler *et al.* (2017)	southern group	Carpathian Basin
**NHMW 39030 (1)**	Austria: Lower Austria: Aspersdorf	**r4**	LT839150	Kindler *et al.* (2017)	northern group	—
**MTD T 9912**	Austria: Lower Austria: Bergern	**r3**	LT839149	Kindler *et al.* (2013)	northern group	—
**NHMW 38541**	Austria: Lower Austria: Ernstbrunn	**r4**	LT839150	Kindler *et al.* (2017)	northern group	—
**NHMW 36728**	Austria: Lower Austria: Grünbach am Schneeberg	**r4**	LT839150	Kindler *et al.* (2017)	northern group	—
**ZFMK 83922**	Austria: Lower Austria: Losenheim	**r9**	LT839155	Kindler *et al.* (2013)	northern group	—
**NHMW 36405 (3)**	Austria: Lower Austria: NE Drösing	**r4**	LT839150	Kindler *et al.* (2017)	northern group	—
**NHMW 36695 (1)**	Austria: Lower Austria: near Hollabrunn	**r3**	LT839149	Kindler *et al.* (2017)	northern group	—
**NHMW 36695 (2)**	Austria: Lower Austria: near Hollabrunn	**r3**	LT839149	Kindler *et al.* (2017)	northern group	—
**NHMW 36405 (2)**	Austria: Lower Austria: Oberedlitz	**r4**	LT839150	Kindler *et al.* (2017)	northern group	—
**NHMW 40084:2**	Austria: Lower Austria: Orth an der Donau	**r3**	LT839149	Kindler *et al.* (2017)	northern group	—
**NHMW 38557 (1)**	Austria: Lower Austria: Perchtoldsdorf	**r4**	LT839150	Kindler *et al.* (2017)	northern group	—
**NHMW 39496**	Austria: Lower Austria: Petronell-Carnuntum	**r3**	LT839149	Kindler *et al.* (2017)	northern group	—
**NHMW 39436 (1)**	Austria: Lower Austria: Sommerein	**r4**	LT839150	Kindler *et al.* (2017)	northern group	—
**NHMW 37311**	Austria: Lower Austria: St. Pölten	**r3**	LT839149	Kindler *et al.* (2017)	northern group	—
**NHMW 38810**	Austria: Lower Austria: St. Valentin	**r3**	LT839149	Kindler *et al.* (2017)	northern group	—
**NHMW 40084:1**	Austria: Lower Austria: Stopfenreuth	**r3**	LT839149	Kindler *et al.* (2017)	northern group	—
**NHMW 36994**	Austria: Styria: Eibiswald	**r4**	LT839150	Kindler *et al.* (2017)	southern group	Carpathian Basin
**MTD T 14544**	Austria: Styria: Graz	**r4**	LT839150	Kindler *et al.* (2017)	southern group	Carpathian Basin
**MTD T 13539**	Austria: Styria: Seewiesen	**r4**	LT839150	Kindler *et al.* (2017)	northern group	—
**NHMW 39266 (3)**	Austria: Upper Austria: between Stadl Paura and Stadl Traun	**r4**	LT839150	Kindler *et al.* (2017)	northern group	—
**NHMW 38946**	Austria: Upper Austria: Mauthausen	**r3**	LT839149	Kindler *et al.* (2017)	northern group	—
**NHMW 40036 (2)**	Austria: Upper Austria: St. Ulrich bei Steyr	**r3**	LT839149	Kindler *et al.* (2017)	northern group	—
**NHMW 38038**	Austria: Upper Austria: St. Ulrich bei Steyr	**r3**	LT839149	Kindler *et al.* (2017)	northern group	—
**NHMW 37422**	Austria: Upper Austria: St. Ulrich bei Steyr	**r3**	LT839149	Kindler *et al.* (2017)	northern group	—
**NHMW 36405 (1)**	Austria: Upper Austria: St. Ulrich bei Steyr	**r3**	LT839149	Kindler *et al.* (2017)	northern group	—
**NHMW 40024 (2)**	Austria: Upper Austria: St. Ulrich bei Steyr	**r3**	LT839149	Kindler *et al.* (2017)	northern group	—
**MTD T 14561**	Austria: Vienna	**r3**	LT839149	Kindler *et al.* (2017)	northern group	—
**MTD T 14562**	Austria: Vienna	**r3**	LT839149	Kindler *et al.* (2017)	northern group	—
**MTD T 9904**	Austria: Vienna: Danube Island	**r19**	LT839165	Kindler *et al.* (2013)	northern group	—
**MTD T 9903**	Austria: Vienna: Danube Island	**r3**	LT839149	Kindler *et al.* (2013)	northern group	—
**MTD T 9905**	Austria: Vienna: Danube Island	**r3**	LT839149	Kindler *et al.* (2013)	northern group	—
**NHMW 36359**	Austria: Vienna: Danube Island	**r3**	LT839149	Kindler *et al.* (2017)	northern group	—
**NHMW 36284**	Austria: Vienna: Danube Island	**r3**	LT839149	Kindler *et al.* (2017)	northern group	—
**NHMW 38557 (2)**	Austria: Vienna: Hadersdorf-Weidlingau	**r3**	LT839149	Kindler *et al.* (2017)	northern group	—
**MTD T 8976**	Bosnia and Herzegovina: Maglič Mountain: Prijevor	**r7**	LT839153	Kindler *et al.* (2013)	southern group	Carpathian Basin

Table S1 continued						
**Voucher**	**Locality**	**Haplotype**	**Accession number**	**Reference**	**Geographical division**	
					**Group**	**Subgroup**
**MTD T 12836**	Bosnia and Herzegovina: near Capljina: Hutovo Blato	**r4**	LT839150	Kindler *et al.* (2017)	southern group	Carpathian Basin
**MTD T 8653**	Bulgaria: Bezhanovo	**r3**	LT839149	Kindler *et al.* (2013)	southern group	southern Balkan
**MTD T 8654**	Bulgaria: Etropole	**r4**	LT839150	Kindler *et al.* (2013)	southern group	southern Balkan
**MTD D 29984**	Bulgaria: Mičurin	**r4**	LT839150	Kindler *et al.* (2013)	southern group	southern Balkan
**MTD T 14453**	Bulgaria: Svištov	**r4**	LT839150	Kindler *et al.* (2017)	southern group	southern Balkan
**MTD T 14454**	Bulgaria: Svištov	**r4**	LT839150	Kindler *et al.* (2017)	southern group	southern Balkan
**ZFMK 54705**	Croatia: 25 km N Rijeka: Gomance	**r18**	LT839164	Kindler *et al.* (2013)	southern group	Carpathian Basin
**ZFMK 92199**	Croatia: Benkovac	**r4**	LT839150	Kindler *et al.* (2013)	southern group	Carpathian Basin
**ZFMK 91558**	Croatia: Istria: NE Lupoglav	**r17**	LT839163	Kindler *et al.* (2013)	southern group	Carpathian Basin
**MTD D 32031**	Croatia: Istria: Rovinj	**r4**	LT839150	Kindler *et al.* (2013)	southern group	Carpathian Basin
**MTD D 32032**	Croatia: Istria: Rovinj	**r4**	LT839150	Kindler *et al.* (2013)	southern group	Carpathian Basin
**MTD D 20790**	Croatia: Krk	**r16**	LT839162	Kindler *et al.* (2013)	southern group	Carpathian Basin
**MTD T 11596**	Croatia: Krk	**r16**	LT839162	Kindler *et al.* (2017)	southern group	Carpathian Basin
**MTD T 11597**	Croatia: Krk	**r16**	LT839162	Kindler *et al.* (2017)	southern group	Carpathian Basin
**MTD T 13249**	Croatia: Krk	**r16**	LT839162	Kindler *et al.* (2017)	southern group	Carpathian Basin
**MTD T 13250**	Croatia: Krk	**r16**	LT839162	Kindler *et al.* (2017)	southern group	Carpathian Basin
**MTD T 13251**	Croatia: Krk	**r18**	LT839164	Kindler *et al.* (2017)	southern group	Carpathian Basin
**ZFMK 49094**	Croatia: Krk: N Krk	**r16**	LT839162	Kindler *et al.* (2013)	southern group	Carpathian Basin
**ZFMK 54706**	Croatia: Krk: Punat	**r16**	LT839162	Kindler *et al.* (2013)	southern group	Carpathian Basin
**ZFMK 54709**	Croatia: near Šibenik: Krka falls	**r11**	LT839157	Kindler *et al.* (2013)	southern group	Carpathian Basin
**NHMW 37852**	Croatia: Pag: Vlasici	**r4**	LT839150	Kindler *et al.* (2017)	southern group	Carpathian Basin
**ZFMK 54707**	Croatia: Plitvice	**r16**	LT839162	Kindler *et al.* (2013)	southern group	Carpathian Basin
**MTD T 11112**	Croatia: Rab: Lopar	**r4**	LT839150	Kindler *et al.* (2017)	southern group	Carpathian Basin
**MTD T 11211**	Croatia: Rab: Lopar	**r4**	LT839150	Kindler *et al.* (2017)	southern group	Carpathian Basin
**MTD T 9873**	Croatia: Vransko Jezero	**r16**	LT839162	Kindler *et al.* (2013)	southern group	Carpathian Basin
**MTD T 9888**	Czech Republic: Bartošovice	**r1**	LT839147	Kindler *et al.* (2013)	northern group	—
**MTD T 9878**	Czech Republic: Běstvina	**r3**	LT839149	Kindler *et al.* (2013)	northern group	—
**MTD T 9579**	Czech Republic: between Horušice and Ruda	**r3**	LT839149	Kindler *et al.* (2013)	northern group	—
**MTD T 9580**	Czech Republic: between Horušice and Ruda	**r4**	LT839150	Kindler *et al.* (2013)	northern group	—
**MTD T 12995**	Czech Republic: Čepice	**r4**	LT839150	Kindler *et al.* (2017)	northern group	—
**MTD T 8963**	Czech Republic: České Budějovice	**r33**	LT839179	Kindler *et al.* (2013)	northern group	—
**MTD T 9886**	Czech Republic: Chřiby Mountains: Halenkovice	**r4**	LT839150	Kindler *et al.* (2013)	northern group	—
**MTD T 9880**	Czech Republic: Chřiby Mountains: Jankovice	**r4**	LT839150	Kindler *et al.* (2013)	northern group	—
**MTD T 9882**	Czech Republic: Chřiby Mountains: Kudlovice	**r4**	LT839150	Kindler *et al.* (2013)	northern group	—
**MTD T 8974**	Czech Republic: Chřiby Mountains: Kudlovická dolina	**r32**	LT839178	Kindler *et al.* (2013)	northern group	—
**MTD T 9879**	Czech Republic: Chřiby Mountains: Salaš	**r4**	LT839150	Kindler *et al.* (2013)	northern group	—
**MTD T 13009**	Czech Republic: Dlouhá Loučka	**r4**	LT839150	Kindler *et al.* (2017)	northern group	—
**MTD T 8636**	Czech Republic: Dolní Lištná	**r3**	LT839149	Kindler *et al.* (2013)	northern group	—
**MTD T 8642**	Czech Republic: Dolní Lištná	**r3**	LT839149	Kindler *et al.* (2013)	northern group	—
**MTD T 10626**	Czech Republic: Dolní Marklovice;	**r4**	LT839150	Kindler *et al.* (2017)	northern group	—
**MTD T 14862**	Czech Republic: Dražovice	**r4**	LT839150	Kindler *et al.* (2017)	northern group	—
**MTD T 12781**	Czech Republic: Fojtka	**r3**	LT839149	Kindler *et al.* (2017)	northern group	—
**MTD T 8962**	Czech Republic: Heřmanovice	**r4**	LT839150	Kindler *et al.* (2013)	northern group	—
**MTD T 8964**	Czech Republic: Heřmanovice	**r4**	LT839150	Kindler *et al.* (2013)	northern group	—

Table S1 continued						
Voucher	Locality	Haplotype	Accession number	Reference	Geographical division	
					Group	Subgroup
**MTD T 8972**	Czech Republic: Kamýk: Litoměřice	r3	LT839149	Kindler *et al.* (2013)	northern group	—
**MTD T 8634**	Czech Republic: Karviná: Olšiny	r4	LT839150	Kindler *et al.* (2013)	northern group	—
**MTD T 14863**	Czech Republic: Klikov	r4	LT839150	Kindler *et al.* (2017)	northern group	—
**MTD T 8966**	Czech Republic: Kokořín	r3	LT839149	Kindler *et al.* (2013)	northern group	—
**MTD T 8965**	Czech Republic: Kokořínský Důl	r4	LT839150	Kindler *et al.* (2013)	northern group	—
**MTD T 8971**	Czech Republic: Kundratice: Litoměřice	r3	LT839149	Kindler *et al.* (2013)	northern group	—
**MTD T 13549**	Czech Republic: Lužnice	r3	LT839149	Kindler *et al.* (2017)	northern group	—
**MTD T 9881**	Czech Republic: Moravský Písek	r4	LT839150	Kindler *et al.* (2013)	northern group	—
**MTD T 14451**	Czech Republic: Na Plachtě, Hradec Králové	r3	LT839149	Kindler *et al.* (2017)	northern group	—
**MTD T 14756**	Czech Republic: Narodní park Podyjí	r4	LT839150	Kindler *et al.* (2017)	northern group	—
**MTD T 13010**	Czech Republic: Olomouc: Černovír	r8	LT839154	Kindler *et al.* (2017)	northern group	—
**MTD T 13007**	Czech Republic: Plinkout	r4	LT839150	Kindler *et al.* (2017)	northern group	—
**MTD T 14755**	Czech Republic: Pouzdřanský rybník	r4	LT839150	Kindler *et al.* (2017)	northern group	—
**MTD T 14861**	Czech Republic: Sadská	r3	LT839149	Kindler *et al.* (2017)	northern group	—
**MTD T 8643**	Czech Republic: Staré Hamry	r3	LT839149	Kindler *et al.* (2013)	northern group	—
**MTD T 14769**	Czech Republic: Staré Město nad Metují	r2	LT839148	Kindler *et al.* (2017)	northern group	—
**MTD T 13008**	Czech Republic: Vlárský průsmyk	r3	LT839149	Kindler *et al.* (2017)	northern group	—
**MTD T 8969**	Czech Republic: Vysoká: Jihlava	r3	LT839149	Kindler *et al.* (2013)	northern group	—
**MTD T 8970**	Czech Republic: Vysoká: Jihlava	r3	LT839149	Kindler *et al.* (2013)	northern group	—
**MTD T 8961**	Czech Republic: Zlaté Hory	r2	LT839148	Kindler *et al.* (2013)	northern group	—
**MTD T 14560**	Germany: Bavaria: E Passau: Erlau	r3	LT839149	Kindler *et al.* (2017)	northern group	—
**MTD T 11136**	Germany: Bavaria: Jochenstein	r3	LT839149	Kindler *et al.* (2017)	northern group	—
**MTD T 11158**	Germany: Bavaria: Klautzenbach	r3	LT839149	Kindler *et al.* (2017)	northern group	—
**ZFMK 71166**	Germany: Bavaria: Mammendorf	r3	LT839149	Kindler *et al.* (2013)	northern group	—
**MTD T 11134**	Germany: Bavaria: Munich	r3	LT839149	Kindler *et al.* (2017)	northern group	—
**MTD T 11149**	Germany: Bavaria: Munich: Feldmoching	r3	LT839149	Kindler *et al.* (2017)	northern group	—
**MTD T 11150**	Germany: Bavaria: Munich: Obermenzing	r3	LT839149	Kindler *et al.* (2017)	northern group	—
**MTD T 11151**	Germany: Bavaria: Munich: Obermenzing	r3	LT839149	Kindler *et al.* (2017)	northern group	—
**MTD T 11152**	Germany: Bavaria: Munich: Obermenzing	r3	LT839149	Kindler *et al.* (2017)	northern group	—
**MTD T 11155**	Germany: Bavaria: Munich: Obermenzing	r3	LT839149	Kindler *et al.* (2017)	northern group	—
**ZSM DNA 171**	Germany: Bavaria: near Bad Aibling	r3	LT839149	Kindler *et al.* (2017)	northern group	—
**MTD T 11139**	Germany: Bavaria: near Haiming	r3	LT839149	Kindler *et al.* (2017)	northern group	—
**MTD T 14352**	Germany: Bavaria: Olching	r3	LT839149	Kindler *et al.* (2017)	northern group	—
**ZFMK 56016**	Germany: Bavaria: Passau	r3	LT839149	Kindler *et al.* (2013)	northern group	—
**MTD T 11135**	Germany: Bavaria: Passau	r3	LT839149	Kindler *et al.* (2017)	northern group	—
**MTD T 11138**	Germany: Bavaria: Schliersee	r3	LT839149	Kindler *et al.* (2017)	northern group	—
**MTD D 29503**	Germany: Brandenburg: Cumlosen	r4	LT839150	Kindler *et al.* (2013)	northern group	—
**MTD T 14362**	Germany: Brandenburg: Golm	r3	LT839149	Kindler *et al.* (2017)	northern group	—
**MTD T 14372**	Germany: Brandenburg: Golm	r3	LT839149	Kindler *et al.* (2017)	northern group	—
**MTD T 14378**	Germany: Brandenburg: Golm	r3	LT839149	Kindler *et al.* (2017)	northern group	—
**ZFMK 82930**	Germany: Hesse: Steinau	r3	LT839149	Kindler *et al.* (2013)	northern group	—
**MTD T 11159**	Germany: Saxony: Airport Dresden	r3	LT839149	Kindler *et al.* (2017)	northern group	—
**MTD T 14481**	Germany: Saxony: Altenberg: Bärenstein	r3	LT839149	Kindler *et al.* (2017)	northern group	—
**MTD D 39331**	Germany: Saxony: Bad Gottleuba	r3	LT839149	Kindler *et al.* (2013)	northern group	—

Table S1 continued						
**Voucher**	**Locality**	**Haplotype**	**Accession number**	**Reference**	**Geographical division**	
					**Group**	**Subgroup**
**MTD D 47231**	Germany: Saxony: Bärnsdorf	**r3**	LT839149	Kindler *et al.* (2013)	northern group	—
**MTD T 14545**	Germany: Saxony: between Mohorn and Helbigsdorf	**r4**	LT839150	Kindler *et al.* (2017)	northern group	—
**MTD T 10997**	Germany: Saxony: between Volkersdorf and Boxdorf	**r28**	LT839174	Kindler *et al.* (2017)	northern group	—
**MTD T 10998**	Germany: Saxony: between Volkersdorf and Boxdorf	**r3**	LT839149	Kindler *et al.* (2017)	northern group	—
**MTD D 31066**	Germany: Saxony: Borna	**r3**	LT839149	Kindler *et al.* (2013)	northern group	—
**MWLK 803/05**	Germany: Saxony: Cunnersdorf	**r3**	LT839149	Kindler *et al.* (2017)	northern group	—
**MTD D 47638**	Germany: Saxony: Dorfhain	**r3**	LT839149	Kindler *et al.* (2013)	northern group	—
**MTD D 47639**	Germany: Saxony: Dresden	**r3**	LT839149	Kindler *et al.* (2013)	northern group	—
**MTD T 13025**	Germany: Saxony: Dresden	**r3**	LT839149	Kindler *et al.* (2017)	northern group	—
**MTD T 14057**	Germany: Saxony: Dresden	**r3**	LT839149	Kindler *et al.* (2017)	northern group	—
**MTD T 14487**	Germany: Saxony: Dresden	**r3**	LT839149	Kindler *et al.* (2017)	northern group	—
**MTD T 12816**	Germany: Saxony: Dresden: Klotzsche	**r3**	LT839149	Kindler *et al.* (2017)	northern group	—
**MTD T 12832**	Germany: Saxony: Dresden: Klotzsche	**r3**	LT839149	Kindler *et al.* (2017)	northern group	—
**MTD D 48178**	Germany: Saxony: Dresden: Langebrück	**r4**	LT839150	Kindler *et al.* (2013)	northern group	—
**MTD D 47258**	Germany: Saxony: Dresden: Weixdorf	**r3**	LT839149	Kindler *et al.* (2013)	northern group	—
**MWLK 432/08**	Germany: Saxony: Fischbach	**r3**	LT839149	Kindler *et al.* (2017)	northern group	—
**MWLK 370/04**	Germany: Saxony: Großdittmannsdorf	**r3**	LT839149	Kindler *et al.* (2013)	northern group	—
**MTD T 14752**	Germany: Saxony: Helbigsdorf	**r4**	LT839150	Kindler *et al.* (2017)	northern group	—
**MWLK 466/05**	Germany: Saxony: Kamenz	**r3**	LT839149	Kindler *et al.* (2013)	northern group	—
**MTD T 10924**	Germany: Saxony: Karsdorf	**r3**	LT839149	Kindler *et al.* (2017)	northern group	—
**MTD T 10925**	Germany: Saxony: Karsdorf	**r3**	LT839149	Kindler *et al.* (2017)	northern group	—
**MTD T 10927**	Germany: Saxony: Karsdorf	**r3**	LT839149	Kindler *et al.* (2017)	northern group	—
**MTD T 10928**	Germany: Saxony: Karsdorf	**r3**	LT839149	Kindler *et al.* (2017)	northern group	—
**MTD T 10933**	Germany: Saxony: Karsdorf	**r3**	LT839149	Kindler *et al.* (2017)	northern group	—
**MTD T 10934**	Germany: Saxony: Karsdorf	**r3**	LT839149	Kindler *et al.* (2017)	northern group	—
**MTD T 10929**	Germany: Saxony: Karsdorf	**r4**	LT839150	Kindler *et al.* (2017)	northern group	—
**MTD T 10932**	Germany: Saxony: Karsdorf	**r4**	LT839150	Kindler *et al.* (2017)	northern group	—
**MTD T 10843**	Germany: Saxony: Kleba	**r3**	LT839149	Kindler *et al.* (2017)	northern group	—
**MTD T 14350**	Germany: Saxony: Klingenberg	**r3**	LT839149	Kindler *et al.* (2017)	northern group	—
**MTD T 14444**	Germany: Saxony: Klingenberg	**r3**	LT839149	Kindler *et al.* (2017)	northern group	—
**MTD T 14445**	Germany: Saxony: Klingenberg	**r3**	LT839149	Kindler *et al.* (2017)	northern group	—
**MTD T 14351**	Germany: Saxony: Klingenberg	**r4**	LT839150	Kindler *et al.* (2017)	northern group	—
**MTD D 40611**	Germany: Saxony: Kreischa-Lockmittel	**r3**	LT839149	Kindler *et al.* (2013)	northern group	—
**MTD T 9528**	Germany: Saxony: Lengefeld / Neunzehnhain	**r3**	LT839149	Kindler *et al.* (2013)	northern group	—
**MTD T 10999**	Germany: Saxony: Liegau-Augustusbad	**r3**	LT839149	Kindler *et al.* (2017)	northern group	—
**MTD T 11227**	Germany: Saxony: Liegau-Augustusbad	**r3**	LT839149	Kindler *et al.* (2017)	northern group	—
**MTD T 11461**	Germany: Saxony: Liegau-Augustusbad	**r3**	LT839149	Kindler *et al.* (2017)	northern group	—
**MTD D 48837**	Germany: Saxony: Liegau-Augustusbad	**r3**	LT839149	Kindler *et al.* (2017)	northern group	—
**MWLK 76/01**	Germany: Saxony: Linz	**r3**	LT839149	Kindler *et al.* (2013)	northern group	—
**MTD T 11107**	Germany: Saxony: Meißen	**r3**	LT839149	Kindler *et al.* (2017)	northern group	—
**MTD T 11108**	Germany: Saxony: Meißen	**r3**	LT839149	Kindler *et al.* (2017)	northern group	—
**MTD T 11396**	Germany: Saxony: Meißen	**r3**	LT839149	Kindler *et al.* (2017)	northern group	—
**MTD T 11398**	Germany: Saxony: Meißen	**r3**	LT839149	Kindler *et al.* (2017)	northern group	—
**MTD T 11400**	Germany: Saxony: Meißen	**r3**	LT839149	Kindler *et al.* (2017)	northern group	—

Table S1 continued						
**Voucher**	**Locality**	**Haplotype**	**Accession number**	**Reference**	**Geographical division**	
					**Group**	**Subgroup**
**MTD T 11031**	Germany: Saxony: Moritzburg	**r3**	LT839149	Kindler *et al.* (2017)	northern group	—
**MTD T 10770**	Germany: Saxony: Moritzburg	**r4**	LT839150	Kindler *et al.* (2017)	northern group	—
**MTD T 10771**	Germany: Saxony: Moritzburg	**r4**	LT839150	Kindler *et al.* (2017)	northern group	—
**MTD T 10772**	Germany: Saxony: Moritzburg	**r4**	LT839150	Kindler *et al.* (2017)	northern group	—
**MTD T 10773**	Germany: Saxony: Moritzburg	**r4**	LT839150	Kindler *et al.* (2017)	northern group	—
**MTD T 11032**	Germany: Saxony: Moritzburg	**r4**	LT839150	Kindler *et al.* (2017)	northern group	—
**MTD T 11033**	Germany: Saxony: Moritzburg	**r4**	LT839150	Kindler *et al.* (2017)	northern group	—
**MTD T 11035**	Germany: Saxony: Moritzburg	**r4**	LT839150	Kindler *et al.* (2017)	northern group	—
**MWLK 665/11**	Germany: Saxony: Naunhof	**r3**	LT839149	Kindler *et al.* (2017)	northern group	—
**MTD D 47429**	Germany: Saxony: Oberschöna	**r3**	LT839149	Kindler *et al.* (2013)	northern group	—
**MWLK 297/10**	Germany: Saxony: Pulsnitz	**r3**	LT839149	Kindler *et al.* (2017)	northern group	—
**MWLK 194/09**	Germany: Saxony: Röhrsdorf	**r3**	LT839149	Kindler *et al.* (2017)	northern group	—
**MTD T 12833**	Germany: Saxony: Seilitz	**r4**	LT839150	Kindler *et al.* (2017)	northern group	—
**MTD T 13015**	Germany: Saxony: Tharandt	**r3**	LT839149	Kindler *et al.* (2017)	northern group	—
**MTD T 10995**	Germany: Saxony: Wermsdorf	**r3**	LT839149	Kindler *et al.* (2017)	northern group	—
**MTD T 10996**	Germany: Saxony: Wermsdorf	**r3**	LT839149	Kindler *et al.* (2017)	northern group	—
**MTD T 11223**	Germany: Saxony: Wermsdorf	**r3**	LT839149	Kindler *et al.* (2017)	northern group	—
**MTD D 25827**	Greece: Cyclades: Paros	**r18**	LT839164	Kindler *et al.* (2013)	southern group	southern Balkan
**ZFMK 58024**	Hungary: Balaton: Balatonszepezd	**r4**	LT839150	Kindler *et al.* (2013)	southern group	Carpathian Basin
**MTD T 12033**	Hungary: Balaton: Balatonszepezd	**r4**	LT839150	Kindler *et al.* (2017)	southern group	Carpathian Basin
**ZFMK 74927**	Hungary: Balaton: Tihany	**r3**	LT839149	Kindler *et al.* (2013)	southern group	Carpathian Basin
**MTD T 11207**	Hungary: Balaton: Tihany	**r3**	LT839149	Kindler *et al.* (2017)	southern group	Carpathian Basin
**MTD T 11858**	Hungary: Balaton: Tihany	**r24**	LT839170	Kindler *et al.* (2017)	southern group	Carpathian Basin
**MTD T 11859**	Hungary: Balaton: Tihany	**r22**	LT839168	Kindler *et al.* (2017)	southern group	Carpathian Basin
**ZFMK 65686**	Hungary: Barcs	**r4**	LT839150	Kindler *et al.* (2013)	southern group	Carpathian Basin
**ZFMK 88062**	Hungary: between Sarród and Fertóujlak	**r4**	LT839150	Kindler *et al.* (2013)	southern group	Carpathian Basin
**MTD T 12942**	Hungary: Bodrogkisfalud	**r3**	LT839149	Kindler *et al.* (2017)	southern group	Carpathian Basin
**ZFMK 61029**	Hungary: Györ	**r3**	LT839149	Kindler *et al.* (2013)	southern group	Carpathian Basin
**ZFMK 91241**	Hungary: Hansági-főcsatorna	**r4**	LT839150	Kindler *et al.* (2013)	southern group	Carpathian Basin
**NHMW 36337**	Hungary: Hortobagy	**r3**	LT839149	Kindler *et al.* (2017)	southern group	Carpathian Basin
**MTD T 9906**	Hungary: Hortobágy	**r3**	LT839149	Kindler *et al.* (2013)	southern group	Carpathian Basin
**MTD T 7574**	Hungary: Kunpeszér	**r3**	LT839149	Kindler *et al.* (2013)	southern group	Carpathian Basin
**MTD T 7571**	Hungary: Kunpeszér	**r4**	LT839150	Kindler *et al.* (2013)	southern group	Carpathian Basin
**MTD T 13547**	Hungary: Létavértes	**r3**	LT839149	Kindler *et al.* (2017)	southern group	Carpathian Basin
**MTD T 12016**	Hungary: Lipót	**r3**	LT839149	Kindler *et al.* (2017)	southern group	Carpathian Basin
**MTD T 11862**	Hungary: near Fülöpstallas	**r30**	LT839176	Kindler *et al.* (2017)	southern group	Carpathian Basin
**MTD T 11863**	Hungary: near Fülöpstallas	**r4**	LT839150	Kindler *et al.* (2017)	southern group	Carpathian Basin
**MTD T 11595**	Hungary: near Kunpeszér: Sarlóspuszta	**r4**	LT839150	Kindler *et al.* (2017)	southern group	Carpathian Basin
**MTD T 12752**	Hungary: near Tokaj	**r26**	LT839172	Kindler *et al.* (2017)	southern group	Carpathian Basin
**MTD T 12756**	Hungary: near Tokaj	**r27**	LT839173	Kindler *et al.* (2017)	southern group	Carpathian Basin
**MTD T 12761**	Hungary: near Tokaj	**r27**	LT839173	Kindler *et al.* (2017)	southern group	Carpathian Basin
**MTD T 12764**	Hungary: near Tokaj	**r27**	LT839173	Kindler *et al.* (2017)	southern group	Carpathian Basin
**MTD T 12751**	Hungary: near Tokaj	**r3**	LT839149	Kindler *et al.* (2017)	southern group	Carpathian Basin
**MTD T 12753**	Hungary: near Tokaj	**r3**	LT839149	Kindler *et al.* (2017)	southern group	Carpathian Basin

Table S1 continued						
**Voucher**	**Locality**	**Haplotype**	**Accession number**	**Reference**	**Geographical division**	
					**Group**	**Subgroup**
**MTD T 12754**	Hungary: near Tokaj	**r3**	LT839149	Kindler *et al.* (2017)	southern group	Carpathian Basin
**MTD T 12755**	Hungary: near Tokaj	**r3**	LT839149	Kindler *et al.* (2017)	southern group	Carpathian Basin
**MTD T 12757**	Hungary: near Tokaj	**r3**	LT839149	Kindler *et al.* (2017)	southern group	Carpathian Basin
**MTD T 12758**	Hungary: near Tokaj	**r3**	LT839149	Kindler *et al.* (2017)	southern group	Carpathian Basin
**MTD T 12759**	Hungary: near Tokaj	**r3**	LT839149	Kindler *et al.* (2017)	southern group	Carpathian Basin
**MTD T 12760**	Hungary: near Tokaj	**r3**	LT839149	Kindler *et al.* (2017)	southern group	Carpathian Basin
**MTD T 12762**	Hungary: near Tokaj	**r3**	LT839149	Kindler *et al.* (2017)	southern group	Carpathian Basin
**MTD T 12763**	Hungary: near Tokaj	**r3**	LT839149	Kindler *et al.* (2017)	southern group	Carpathian Basin
**MTD T 12765**	Hungary: near Tokaj	**r3**	LT839149	Kindler *et al.* (2017)	southern group	Carpathian Basin
**MTD T 12766**	Hungary: near Tokaj	**r3**	LT839149	Kindler *et al.* (2017)	southern group	Carpathian Basin
**MTD T 12767**	Hungary: near Tokaj	**r3**	LT839149	Kindler *et al.* (2017)	southern group	Carpathian Basin
**MTD T 12768**	Hungary: near Tokaj	**r3**	LT839149	Kindler *et al.* (2017)	southern group	Carpathian Basin
**MTD T 12769**	Hungary: near Tokaj	**r3**	LT839149	Kindler *et al.* (2017)	southern group	Carpathian Basin
**MTD T 12770**	Hungary: near Tokaj	**r3**	LT839149	Kindler *et al.* (2017)	southern group	Carpathian Basin
**MTD T 12771**	Hungary: near Tokaj	**r3**	LT839149	Kindler *et al.* (2017)	southern group	Carpathian Basin
**MTD T 12772**	Hungary: near Tokaj	**r3**	LT839149	Kindler *et al.* (2017)	southern group	Carpathian Basin
**MTD T 12773**	Hungary: near Tokaj	**r3**	LT839149	Kindler *et al.* (2017)	southern group	Carpathian Basin
**MTD T 12774**	Hungary: near Tokaj	**r3**	LT839149	Kindler *et al.* (2017)	southern group	Carpathian Basin
**MTD T 12775**	Hungary: near Tokaj	**r3**	LT839149	Kindler *et al.* (2017)	southern group	Carpathian Basin
**MTD T 12776**	Hungary: near Tokaj	**r3**	LT839149	Kindler *et al.* (2017)	southern group	Carpathian Basin
**MTD T 12777**	Hungary: near Tokaj	**r3**	LT839149	Kindler *et al.* (2017)	southern group	Carpathian Basin
**MTD T 12778**	Hungary: near Tokaj	**r3**	LT839149	Kindler *et al.* (2017)	southern group	Carpathian Basin
**MTD T 12779**	Hungary: near Tokaj	**r3**	LT839149	Kindler *et al.* (2017)	southern group	Carpathian Basin
**MTD T 12780**	Hungary: near Tokaj	**r3**	LT839149	Kindler *et al.* (2017)	southern group	Carpathian Basin
**ZFMK 85905**	Hungary: S Budapest	**r4**	LT839150	Kindler *et al.* (2013)	southern group	Carpathian Basin
**MTD T 11918**	Hungary: Szeged: Lake Feher	**r14**	LT839160	Kindler *et al.* (2017)	southern group	Carpathian Basin
**MTD T 11920**	Hungary: Szeged: Lake Feher	**r3**	LT839149	Kindler *et al.* (2017)	southern group	Carpathian Basin
**MTD T 12014**	Hungary: Szeged: Lake Feher	**r3**	LT839149	Kindler *et al.* (2017)	southern group	Carpathian Basin
**MTD T 12015**	Hungary: Szeged: Lake Feher	**r3**	LT839149	Kindler *et al.* (2017)	southern group	Carpathian Basin
**MTD T 11864**	Hungary: Tiszaalpár	**r3**	LT839149	Kindler *et al.* (2017)	southern group	Carpathian Basin
**MTD T 11865**	Hungary: Tiszaalpár	**r3**	LT839149	Kindler *et al.* (2017)	southern group	Carpathian Basin
**MTD T 11867**	Hungary: Tiszaalpár	**r3**	LT839149	Kindler *et al.* (2017)	southern group	Carpathian Basin
**MTD T 11979**	Hungary: Tömörd	**r4**	LT839150	Kindler *et al.* (2017)	southern group	Carpathian Basin
**MTD T 11980**	Hungary: Tömörd	**r4**	LT839150	Kindler *et al.* (2017)	southern group	Carpathian Basin
**MTD T 7594**	Hungary: Villany	**r4**	LT839150	Kindler *et al.* (2013)	southern group	Carpathian Basin
**MTD T 14459**	Kosovo: Ferizaj	**r20**	LT839166	Kindler *et al.* (2017)	southern group	southern Balkan
**MTD T 14458**	Kosovo: Nerodime e Poshtme	**r20**	LT839166	Kindler *et al.* (2017)	southern group	southern Balkan
**MTD T 14775**	Montenegro: Bjelasica	**r4**	LT839150	Kindler *et al.* (2017)	southern group	southern Balkan
**MTD T 12793**	Poland: Małopolska: Książ Wielki	**r3**	LT839149	Kindler *et al.* (2017)	northern group	—
**MTD T 12830**	Poland: Małopolska: Książ Wielki	**r3**	LT839149	Kindler *et al.* (2017)	northern group	—
**MTD T 12831**	Poland: Małopolska: Książ Wielki	**r3**	LT839149	Kindler *et al.* (2017)	northern group	—
**MTD T 9965**	Poland: Mazovia: Kampinos National Park	**r3**	LT839149	Kindler *et al.* (2013)	northern group	—
**MTD T 9966**	Poland: Mazovia: Kampinos National Park	**r3**	LT839149	Kindler *et al.* (2013)	northern group	—
**MTD T 9968**	Poland: Mazovia: Kampinos National Park	**r3**	LT839149	Kindler *et al.* (2013)	northern group	—

Table S1 continued						
**Voucher**	**Locality**	**Haplotype**	**Accession number**	**Reference**	**Geographical division**	
					**Group**	**Subgroup**
**MTD T 9969**	Poland: Mazovia: Kampinos National Park	r3	LT839149	Kindler *et al.* (2013)	northern group	—
**MTD T 9972**	Poland: Mazovia: Kampinos National Park	r3	LT839149	Kindler *et al.* (2013)	northern group	—
**MTD T 9974**	Poland: Mazovia: Kampinos National Park	r3	LT839149	Kindler *et al.* (2013)	northern group	—
**MTD T 10318**	Poland: Mazovia: Kampinos National Park	r3	LT839149	Kindler *et al.* (2013)	northern group	—
**MTD T 9637**	Poland: Upper Silesia: Golczowice	r2	LT839148	Kindler *et al.* (2013)	northern group	—
**MTD T 10025**	Poland: Upper Silesia: Kotórz Wielki	r3	LT839149	Kindler *et al.* (2013)	northern group	—
**MTD T 9635**	Poland: Upper Silesia: Krośnica	r4	LT839150	Kindler *et al.* (2013)	northern group	—
**MTD T 9638**	Poland: Upper Silesia: Ligota Tułowicka	r2	LT839148	Kindler *et al.* (2013)	northern group	—
**MTD T 9636**	Poland: Upper Silesia: SE Opole	r4	LT839150	Kindler *et al.* (2013)	northern group	—
**MTD T 8640**	Romania: Cerna Sat	r6	LT839152	Kindler *et al.* (2013)	southern group	Carpathian Basin
**MTD T 9889**	Romania: Chilia Veche	r3	LT839149	Kindler *et al.* (2013)	southern group	southern Balkan
**MTD T 11604**	Romania: Crivina	r5	LT839151	Kindler *et al.* (2017)	southern group	Carpathian Basin
**MTD T 8650**	Romania: Geoagiu de Sus	r29	LT839175	Kindler *et al.* (2013)	southern group	Carpathian Basin
**MTD T 13542**	Romania: Tarnovita	r3	LT839149	Kindler *et al.* (2017)	southern group	Carpathian Basin
**MTD T 14456**	Serbia: Belo Polje	r4	LT839150	Kindler *et al.* (2017)	southern group	Carpathian Basin
**MTD T 14455**	Serbia: Belo Polje	r4	LT839150	Kindler *et al.* (2017)	southern group	Carpathian Basin
**MTD T 14457**	Serbia: Dići	r4	LT839150	Kindler *et al.* (2017)	southern group	Carpathian Basin
**MTD T 12999**	Serbia: Donji Dušnik	r4	LT839150	Kindler *et al.* (2017)	southern group	southern Balkan
**MTD T 8975**	Slovakia: between Topoľa and Runina	r3	LT839149	Kindler *et al.* (2013)	southern group	Carpathian Basin
**MTD T 12961**	Slovakia: Bodíky	r3	LT839149	Kindler *et al.* (2017)	southern group	Carpathian Basin
**MTD T 12962**	Slovakia: Bodíky	r3	LT839149	Kindler *et al.* (2017)	southern group	Carpathian Basin
**MTD T 12963**	Slovakia: Bodíky	r3	LT839149	Kindler *et al.* (2017)	southern group	Carpathian Basin
**MTD T 12960**	Slovakia: Bodíky	r4	LT839150	Kindler *et al.* (2017)	southern group	Carpathian Basin
**MTD T 12966**	Slovakia: Bratislava-Dúbravka	r3	LT839149	Kindler *et al.* (2017)	northern group	—
**MTD T 12998**	Slovakia: Bratislava-Dúbravka	r4	LT839150	Kindler *et al.* (2017)	northern group	—
**MTD T 12967**	Slovakia: Bratislava-Karlova Ves	r3	LT839149	Kindler *et al.* (2017)	northern group	—
**MTD T 12964**	Slovakia: Bratislava-Karlova Ves	r4	LT839150	Kindler *et al.* (2017)	northern group	—
**MTD T 14764**	Slovakia: Bratislava-Petržalka	r3	LT839149	Kindler *et al.* (2017)	southern group	Carpathian Basin
**MTD T 14765**	Slovakia: Bratislava-Petržalka	r3	LT839149	Kindler *et al.* (2017)	southern group	Carpathian Basin
**MTD T 14766**	Slovakia: Bratislava-Petržalka	r3	LT839149	Kindler *et al.* (2017)	southern group	Carpathian Basin
**MTD T 14767**	Slovakia: Bratislava-Petržalka	r3	LT839149	Kindler *et al.* (2017)	southern group	Carpathian Basin
**MTD T 10938**	Slovakia: Bratislava: Devínská Kobyla	r3	LT839149	Kindler *et al.* (2017)	northern group	—
**MTD T 12944**	Slovakia: Bratislava: Železná Studnička	r3	LT839149	Kindler *et al.* (2017)	northern group	—
**MTD T 12945**	Slovakia: Bratislava: Železná Studnička	r3	LT839149	Kindler *et al.* (2017)	northern group	—
**MTD T 12946**	Slovakia: Bratislava: Železná Studnička	r3	LT839149	Kindler *et al.* (2017)	northern group	—
**MTD T 12969**	Slovakia: Bratislava: Železná Studnička	r3	LT839149	Kindler *et al.* (2017)	northern group	—
**MTD T 12968**	Slovakia: Bratislava: Železná Studnička	r4	LT839150	Kindler *et al.* (2017)	northern group	—
**MTD T 12970**	Slovakia: Bratislava: Železná Studnička	r4	LT839150	Kindler *et al.* (2017)	northern group	—
**MTD T 9017**	Slovakia: Bujakovo	r3	LT839149	Kindler *et al.* (2013)	southern group	Carpathian Basin
**MTD T 8648**	Slovakia: Červený Kláštor	r3	LT839149	Kindler *et al.* (2013)	southern group	Carpathian Basin
**MTD T 14452**	Slovakia: Chotínske Piesky	r8	LT839154	Kindler *et al.* (2017)	southern group	Carpathian Basin
**MTD T 14450**	Slovakia: Čunovo	r4	LT839150	Kindler *et al.* (2017)	southern group	Carpathian Basin
**MTD T 9014**	Slovakia: Devín	r3	LT839149	Kindler *et al.* (2013)	northern group	—
**MTD T 14760**	Slovakia: Devínske Jazero	r3	LT839149	Kindler *et al.* (2017)	northern group	—

Table S1 continued						
**Voucher**	**Locality**	**Haplotype**	**Accession number**	**Reference**	**Geographical division**	
					**Group**	**Subgroup**
**MTD T 14761**	Slovakia: Devínské Jazero	**r3**	LT839149	Kindler *et al.* (2017)	northern group	—
**MTD T 12996**	Slovakia: Dunajská Lužná	**r3**	LT839149	Kindler *et al.* (2017)	southern group	Carpathian Basin
**MTD T 12997**	Slovakia: Dunajská Lužná	**r4**	LT839150	Kindler *et al.* (2017)	southern group	Carpathian Basin
**MTD T 14763**	Slovakia: Gbelce: Parížské močiare	**r4**	LT839150	Kindler *et al.* (2017)	southern group	Carpathian Basin
**MTD T 14448**	Slovakia: Istragov	**r30**	LT839176	Kindler *et al.* (2017)	southern group	Carpathian Basin
**MTD T 8641**	Slovakia: Janova Lehota	**r3**	LT839149	Kindler *et al.* (2013)	southern group	Carpathian Basin
**MTD T 9011**	Slovakia: Jurský Šúr	**r4**	LT839150	Kindler *et al.* (2013)	southern group	Carpathian Basin
**MTD T 14754**	Slovakia: Kalinkovo-Hrušovská zdrž	**r3**	LT839149	Kindler *et al.* (2017)	southern group	Carpathian Basin
**MTD T 8635**	Slovakia: Kamenica nad Hronom	**r8**	LT839154	Kindler *et al.* (2013)	southern group	Carpathian Basin
**MTD T 12983**	Slovakia: Kosorín	**r4**	LT839150	Kindler *et al.* (2017)	southern group	Carpathian Basin
**MTD T 14772**	Slovakia: Kysak: Hornád river	**r3**	LT839149	Kindler *et al.* (2017)	southern group	Carpathian Basin
**MTD T 14776**	Slovakia: Liptovský Hrádok: Belá river	**r1**	LT839147	Kindler *et al.* (2017)	southern group	Carpathian Basin
**MTD T 14762**	Slovakia: Maňa: Žitavský luh	**r4**	LT839150	Kindler *et al.* (2017)	southern group	Carpathian Basin
**MTD T 14773**	Slovakia: Margecany: Ružín Dam	**r3**	LT839149	Kindler *et al.* (2017)	southern group	Carpathian Basin
**MTD T 12947**	Slovakia: Marianka	**r3**	LT839149	Kindler *et al.* (2017)	northern group	—
**MTD T 12952**	Slovakia: Marianka	**r3**	LT839149	Kindler *et al.* (2017)	northern group	—
**MTD T 12953**	Slovakia: Marianka	**r3**	LT839149	Kindler *et al.* (2017)	northern group	—
**MTD T 12955**	Slovakia: Marianka	**r3**	LT839149	Kindler *et al.* (2017)	northern group	—
**MTD T 12948**	Slovakia: Marianka	**r4**	LT839150	Kindler *et al.* (2017)	northern group	—
**MTD T 12950**	Slovakia: Marianka	**r4**	LT839150	Kindler *et al.* (2017)	northern group	—
**MTD T 12951**	Slovakia: Marianka	**r4**	LT839150	Kindler *et al.* (2017)	northern group	—
**MTD T 12954**	Slovakia: Marianka	**r4**	LT839150	Kindler *et al.* (2017)	northern group	—
**MTD T 12956**	Slovakia: Marianka	**r4**	LT839150	Kindler *et al.* (2017)	northern group	—
**MTD T 12957**	Slovakia: Marianka	**r4**	LT839150	Kindler *et al.* (2017)	northern group	—
**MTD T 12958**	Slovakia: Marianka	**r4**	LT839150	Kindler *et al.* (2017)	northern group	—
**MTD T 12959**	Slovakia: Marianka	**r4**	LT839150	Kindler *et al.* (2017)	northern group	—
**MTD T 12949**	Slovakia: Marianka	**r8**	LT839154	Kindler *et al.* (2017)	northern group	—
**MTD T 14774**	Slovakia: Michalovce: Šíravský kanál	**r3**	LT839149	Kindler *et al.* (2017)	southern group	Carpathian Basin
**MTD T 9019**	Slovakia: Muránska Lehota	**r31**	LT839177	Kindler *et al.* (2013)	southern group	Carpathian Basin
**MTD T 12943**	Slovakia: near Marcelová	**r4**	LT839150	Kindler *et al.* (2017)	southern group	Carpathian Basin
**MTD T 9018**	Slovakia: Regetovka	**r3**	LT839149	Kindler *et al.* (2013)	southern group	Carpathian Basin
**MTD T 14449**	Slovakia: Rusovce	**r23**	LT839169	Kindler *et al.* (2017)	southern group	Carpathian Basin
**MTD T 9016**	Slovakia: Rusovce	**r4**	LT839150	Kindler *et al.* (2013)	southern group	Carpathian Basin
**MTD T 9009**	Slovakia: Silica: Farárova jama	**r4**	LT839150	Kindler *et al.* (2013)	southern group	Carpathian Basin
**MTD T 8637**	Slovakia: Strážne	**r3**	LT839149	Kindler *et al.* (2013)	southern group	Carpathian Basin
**MTD T 9020**	Slovakia: Stupava	**r8**	LT839154	Kindler *et al.* (2013)	northern group	—
**MTD T 12977**	Slovakia: Svätý Jur	**r15**	LT839161	Kindler *et al.* (2017)	southern group	Carpathian Basin
**MTD T 12971**	Slovakia: Svätý Jur	**r3**	LT839149	Kindler *et al.* (2017)	southern group	Carpathian Basin
**MTD T 12972**	Slovakia: Svätý Jur	**r3**	LT839149	Kindler *et al.* (2017)	southern group	Carpathian Basin
**MTD T 12974**	Slovakia: Svätý Jur	**r3**	LT839149	Kindler *et al.* (2017)	southern group	Carpathian Basin
**MTD T 12981**	Slovakia: Svätý Jur	**r3**	LT839149	Kindler *et al.* (2017)	southern group	Carpathian Basin
**MTD T 10937**	Slovakia: Svätý Jur	**r4**	LT839150	Kindler *et al.* (2017)	southern group	Carpathian Basin
**MTD T 12973**	Slovakia: Svätý Jur	**r4**	LT839150	Kindler *et al.* (2017)	southern group	Carpathian Basin
**MTD T 12975**	Slovakia: Svätý Jur	**r4**	LT839150	Kindler *et al.* (2017)	southern group	Carpathian Basin

Table S1 continued						
Voucher	Locality	Haplotype	Accession number	Reference	Geographical division	
					Group	Subgroup
**MTD T 12976**	Slovakia: Svätý Jur	**r4**	LT839150	Kindler *et al.* (2017)	southern group	Carpathian Basin
**MTD T 12978**	Slovakia: Svätý Jur	**r4**	LT839150	Kindler *et al.* (2017)	southern group	Carpathian Basin
**MTD T 12979**	Slovakia: Svätý Jur	**r4**	LT839150	Kindler *et al.* (2017)	southern group	Carpathian Basin
**MTD T 12982**	Slovakia: Svätý Jur	**r4**	LT839150	Kindler *et al.* (2017)	southern group	Carpathian Basin
**MTD T 12980**	Slovakia: Svätý Jur	**r8**	LT839154	Kindler *et al.* (2017)	southern group	Carpathian Basin
**MTD T 8638**	Slovakia: Svetlice	**r3**	LT839149	Kindler *et al.* (2013)	southern group	Carpathian Basin
**MTD T 9022**	Slovakia: Ulič	**r3**	LT839149	Kindler *et al.* (2013)	southern group	Carpathian Basin
**MTD T 14768**	Slovakia: Veľké Leváre	**r32**	LT839178	Kindler *et al.* (2017)	northern group	—
**MTD T 9010**	Slovakia: Weitov lom	**r3**	LT839149	Kindler *et al.* (2013)	northern group	—
**MTD T 10899**	Slovenia: Bela krajina: Zjot	**r21**	LT839167	Kindler *et al.* (2017)	southern group	Carpathian Basin
**MTD T 12729**	Slovenia: Bilje: Vipava river	**r4**	LT839150	Kindler *et al.* (2017)	southern group	Carpathian Basin
**MTD T 11526**	Slovenia: Bled	**r4**	LT839150	Kindler *et al.* (2017)	southern group	Carpathian Basin
**MTD T 13540**	Slovenia: Borjana	**r4**	LT839150	Kindler *et al.* (2017)	southern group	Carpathian Basin
**MTD T 11116**	Slovenia: Borovnica	**r4**	LT839150	Kindler *et al.* (2017)	southern group	Carpathian Basin
**MTD T 11209**	Slovenia: Borovnica	**r4**	LT839150	Kindler *et al.* (2017)	southern group	Carpathian Basin
**MTD T 12783**	Slovenia: Celje: Voglajna river	**r4**	LT839150	Kindler *et al.* (2017)	southern group	Carpathian Basin
**MTD T 11535**	Slovenia: Cerknica Lake	**r4**	LT839150	Kindler *et al.* (2017)	southern group	Carpathian Basin
**MTD T 11536**	Slovenia: Cerknica Lake	**r4**	LT839150	Kindler *et al.* (2017)	southern group	Carpathian Basin
**MTD T 11537**	Slovenia: Cerknica Lake	**r4**	LT839150	Kindler *et al.* (2017)	southern group	Carpathian Basin
**MTD T 11529**	Slovenia: Cerknica Lake	**r4**	LT839150	Kindler *et al.* (2017)	southern group	Carpathian Basin
**MTD T 11538**	Slovenia: Cerknica Lake	**r4**	LT839150	Kindler *et al.* (2017)	southern group	Carpathian Basin
**MTD T 13541**	Slovenia: Dragonje	**r4**	LT839150	Kindler *et al.* (2017)	southern group	Carpathian Basin
**MTD T 10888**	Slovenia: Koper: Škocjanski zatok	**r17**	LT839163	Kindler *et al.* (2017)	southern group	Carpathian Basin
**MTD T 10889**	Slovenia: Koper: Škocjanski zatok	**r17**	LT839163	Kindler *et al.* (2017)	southern group	Carpathian Basin
**MTD T 10890**	Slovenia: Koper: Škocjanski zatok	**r17**	LT839163	Kindler *et al.* (2017)	southern group	Carpathian Basin
**MTD T 10891**	Slovenia: Koper: Škocjanski zatok	**r17**	LT839163	Kindler *et al.* (2017)	southern group	Carpathian Basin
**MTD T 10887**	Slovenia: Koper: Škocjanski zatok	**r4**	LT839150	Kindler *et al.* (2017)	southern group	Carpathian Basin
**MTD T 10892**	Slovenia: Koper: Škocjanski zatok	**r4**	LT839150	Kindler *et al.* (2017)	southern group	Carpathian Basin
**ZFMK 65382**	Slovenia: Ljubljana	**r4**	LT839150	Kindler *et al.* (2013)	southern group	Carpathian Basin
**MTD T 14346**	Slovenia: Ljubljana moor: Curnovec	**r4**	LT839150	Kindler *et al.* (2017)	southern group	Carpathian Basin
**MTD T 14349**	Slovenia: Ljubljana moor: Deponija Vic	**r4**	LT839150	Kindler *et al.* (2017)	southern group	Carpathian Basin
**MTD T 10885**	Slovenia: Ljubljana moor: Draga pri Igu	**r4**	LT839150	Kindler *et al.* (2017)	southern group	Carpathian Basin
**MTD T 10886**	Slovenia: Ljubljana moor: Draga pri Igu	**r4**	LT839150	Kindler *et al.* (2017)	southern group	Carpathian Basin
**MTD T 14347**	Slovenia: Ljubljana moor: Draga pri Igu	**r4**	LT839150	Kindler *et al.* (2017)	southern group	Carpathian Basin
**MTD T 14348**	Slovenia: Ljubljana moor: Draga pri Igu	**r4**	LT839150	Kindler *et al.* (2017)	southern group	Carpathian Basin
**MTD T 11533**	Slovenia: Ljubljana: Večna pot	**r16**	LT839162	Kindler *et al.* (2017)	southern group	Carpathian Basin
**MTD T 11212**	Slovenia: Ljubljansko barje	**r4**	LT839150	Kindler *et al.* (2017)	southern group	Carpathian Basin
**MTD T 11213**	Slovenia: Ljubljansko barje: Bistra	**r4**	LT839150	Kindler *et al.* (2017)	southern group	Carpathian Basin
**MTD T 11534**	Slovenia: Ljubljansko barje: Log pri Brezovici	**r4**	LT839150	Kindler *et al.* (2017)	southern group	Carpathian Basin
**MTD T 10900**	Slovenia: Ljubljansko barje: Pako	**r4**	LT839150	Kindler *et al.* (2017)	southern group	Carpathian Basin
**MTD T 10901**	Slovenia: Ljubljansko barje: Pako	**r4**	LT839150	Kindler *et al.* (2017)	southern group	Carpathian Basin
**MTD T 10902**	Slovenia: Ljubljansko barje: Pako	**r4**	LT839150	Kindler *et al.* (2017)	southern group	Carpathian Basin
**MTD T 12784**	Slovenia: Lukavci	**r4**	LT839150	Kindler *et al.* (2017)	southern group	Carpathian Basin
**MTD T 10895**	Slovenia: Lukavci near Ljutomer	**r12**	LT839158	Kindler *et al.* (2017)	southern group	Carpathian Basin

Table S1 continued						
Voucher	Locality	Haplotype	Accession number	Reference	Geographical division	
					Group	Subgroup
**MTD T 10893**	Slovenia: Lukavci near Ljutomer	**r4**	LT839150	Kindler *et al.* (2017)	southern group	Carpathian Basin
**MTD T 10894**	Slovenia: Lukavci near Ljutomer	**r4**	LT839150	Kindler *et al.* (2017)	southern group	Carpathian Basin
**MTD T 11113**	Slovenia: Mele	**r4**	LT839150	Kindler *et al.* (2017)	southern group	Carpathian Basin
**MTD T 11114**	Slovenia: Murski Petrovci	**r25**	LT839171	Kindler *et al.* (2017)	southern group	Carpathian Basin
**MTD T 11527**	Slovenia: near Maribor: Cigonca Forest	**r8**	LT839154	Kindler *et al.* (2017)	southern group	Carpathian Basin
**MTD T 11530**	Slovenia: near Maribor: Racki Ribnik	**r4**	LT839150	Kindler *et al.* (2017)	southern group	Carpathian Basin
**MTD T 11208**	Slovenia: Podlipa	**r4**	LT839150	Kindler *et al.* (2017)	southern group	Carpathian Basin
**MTD T 11531**	Slovenia: Pragersko	**r4**	LT839150	Kindler *et al.* (2017)	southern group	Carpathian Basin
**MTD T 12178**	Slovenia: Sentjur	**r4**	LT839150	Kindler *et al.* (2017)	southern group	Carpathian Basin
**MTD T 11528**	Slovenia: Slovenia: Divača	**r4**	LT839150	Kindler *et al.* (2017)	southern group	Carpathian Basin
**MTD T 11115**	Slovenia: Tišina	**r4**	LT839150	Kindler *et al.* (2017)	southern group	Carpathian Basin
**MTD T 11210**	Slovenia: Zadolje	**r17**	LT839163	Kindler *et al.* (2017)	southern group	Carpathian Basin
**ZFMK 38356**	Sweden: Gotland	**r3**	LT839149	Kindler *et al.* (2013)	northern group	—

Museum acronyms of vouchers for Table S1:
BEV – Laboratoire de Biogéographie et Ecologie des Vertébrés, Centre d'Ecologie Fonctionnelle & Evolutive, Montpellier
LMNM – Landesmuseum Natur und Mensch, Oldenburg
MTD D – Museum of Zoology, Senckenberg Dresden (Herpetological Collection)
MTD T – Museum of Zoology, Senckenberg Dresden (Tissue Collection)
MWLK – Museum der Westlausitz, Kamenz
NHMW – Naturhistorisches Museum Wien
NME – Naturkundemuseum Erfurt
SMF – Forschungsinstitut und Naturmuseum Senckenberg, Frankfurt a.M.
SMNS – Staatliches Museum für Naturkunde Stuttgart
SMNK – Staatliches Museum für Naturkunde Karlsruhe
ZFMK – Zoologisches Forschungsmuseum Alexander Koenig, Bonn
ZMB – Museum für Naturkunde Berlin
ZMH – Zoologisches Museum Hamburg
ZMUO – Zoologisk museum, Universitetet i Oslo
ZSM – Zoologische Staatssammlung München
ZSUM – Zoologische Sammlung der Philipps-Universität Marburg

Remaining acronyms refer to samples from the collection of the Department of Environmental Sciences, Section of Conservation Biology, University of Basel

Table S2. Diversity indices for rarefied sampling of the northern group of the blue lineage.

	n	***S***	***h***	**h_P**	***Hd***	**π (*10^{-3})**
**Rarefied sampling 1**	33	2	3	2	0.119	0.14
**Rarefied sampling 2**	33	1	2	1	0.061	0.07
**Rarefied sampling 3**	33	1	2	1	0.061	0.07
**Rarefied sampling 4**	33	2	3	2	0.119	0.14
**Rarefied sampling 5**	33	2	3	2	0.174	0.21
**Average**	**33**	**2**	**3**	**2**	**0.107**	**0.13**

n = sample size; *S* = number of segregating sites; *h* = number of haplotypes; h_P = number of private haplotypes; *Hd* = Haplotype diversity; π = nucleotide diversity.

Table S3. Diversity indices for rarefied sampling of the northern group of the yellow lineage.

	n	***S***	***h***	**h_P**	***Hd***	**π (*10^{-3})**
**Rarefied sampling 1**	27	6	7	6	0.456	0.59
**Rarefied sampling 2**	27	4	5	4	0.279	0.34
**Rarefied sampling 3**	27	3	4	3	0.336	0.41
**Rarefied sampling 4**	27	6	7	6	0.456	0.59
**Rarefied sampling 5**	27	5	6	5	0.399	0.51
**Average**	**27**	**5**	**5**	**5**	**0.385**	**0.49**

n = sample size; *S* = number of segregating sites; *h* = number of haplotypes; h_P = number of private haplotypes; *Hd* = Haplotype diversity; π = nucleotide diversity.

Table S4. Diversity indices for rarefied sampling of the northern Central European and Scandinavian subgroup (yellow lineage).

	n	***S***	***h***	**h_P**	***Hd***	**π (*10^{-3})**
**Rarefied sampling 1**	165	17	17	14	0.392	0.51
**Rarefied sampling 2**	165	12	13	10	0.374	0.47
**Rarefied sampling 3**	165	14	14	11	0.356	0.46
**Rarefied sampling 4**	165	19	20	17	0.445	0.58
**Rarefied sampling 5**	165	19	19	17	0.428	0.58
**Average**	**165**	**16**	**17**	**14**	**0.399**	**0.52**

n = sample size; *S* = number of segregating sites; *h* = number of haplotypes; h_P = number of private haplotypes; *Hd* = Haplotype diversity; π = nucleotide diversity.

Table S5. Diversity indices for rarefied sampling of the northern group and of the Carpathian Basin subgroup of the red lineage.

**Northern group:**	***n***	***S***	***h***	**h_P**	***Hd***	**π (*10^{-3})**
**Rarefied sampling 1**	11	2	2	0	0.182	0.42
**Rarefied sampling 2**	11	2	2	0	0.182	0.42
**Rarefied sampling 3**	11	4	4	2	0.600	1.43
**Rarefied sampling 4**	11	2	2	0	0.436	1.01
**Rarefied sampling 5**	11	2	2	0	0.327	0.76
**Average**	**11**	**2**	**2**	**0**	**0.345**	**0.81**
**Carpathian Basin subgroup:**						
**Rarefied sampling 1**	11	6	6	4	0.800	2.02
**Rarefied sampling 2**	11	4	3	0	0.473	1.18
**Rarefied sampling 3**	11	5	5	3	0.818	2.23
**Rarefied sampling 4**	11	4	4	2	0.709	1.68
**Rarefied sampling 5**	11	5	4	2	0.600	1.64
**Average**	**11**	**5**	**4**	**2**	**0.680**	**1.75**

n = sample size; *S* = number of segregating sites; *h* = number of haplotypes; h_P = number of private haplotypes; *Hd* = Haplotype diversity; π = nucleotide diversity.

Table S6. Values for mismatch distribution models and neutrality tests for all ND4+tRNA sequences of the three grass snake lineages.

Group	*n*	Mismatch distribution			*Test of goodness-of-fit*		Raggedness index		Tajima's *D*		Fu's F_S	
		τ	θ_0	θ_1	SSD	*P*	rg	*P*	*D*	*P*	F_S	*P*
**Blue lineage *(Natrix helvetica)***												
**Northern group**	370	3.000 (0.430 – 3.000)	0.000 (0.000 – 0.007)	0.148 (0.000 – 99999.000)	0.00023	0.270	0.582	0.750	-1.839*	0.000	-13.724*	0.000
**Southern group**	33	3.000 (0.385 – 3.500)	0.000 (0.000 – 0.014)	0.225 (0.000 – 99999.000)	0.00096	0.400	0.454	0.660	-1.728*	0.011	-3.543*	0.000
**Yellow lineage *(Natrix natrix)***												
**Northern group**	499	0.498 (0.369 – 0.943)	0.000 (0.000 – 0.118)	99999.000 (20.523 – 99999.000)	0.00129	0.250	0.159	0.430	-2.462*	0.000	-29.634*	0.000
**Southern group**	27	7.578 (0.451 – 62.578)	0.000 (0.000 – 1.338)	2.804 (0.831 – 99999.000)	0.09426	0.080	0.127	0.280	0.343	0.696	0.015	0.520
**NCESS**	334	0.533 (0.346 – 1.102)	0.009 (0.000 – 0.069)	99999.000 (33.688 – 99999.000)	0.00224	0.200	0.144	0.230	-2.407*	0.000	-29.148*	0.000
**SCES**	165	3.000 (0.000 – 4.406)	0.000 (0.000 – 0.004)	0.491 (0.000 – 99999.000)	0.00525	0.450	0.217	0.640	-1.863*	0.007	-10.969*	0.000
**Red lineage *(Natrix natrix)***												
**Northern group**	202	2.691 (0.000 – 5.252)	0.000 (0.000 – 0.654)	1.261 (0.064 – 99999.000)	0.09740	0.190	0.406	0.160	-0.954	0.170	-2.956	0.126
**Southern group**	241	2.385 (0.186 – 4.752)	0.000 (0.000 – 0.178)	3.021 (2.012 – 99999.000)	0.04353	0.140	0.158	0.080	-2.080*	0.000	-20.614*	0.000
**Carpathian Basin subgroup**	230	2.363 (0.000 – 4,939)	0.000 (0.000 – 0.670)	2.999 (0.409 – 99999.000)	0.04066	0.180	0.147	0.170	-2.042*	0.000	-19.426*	0.000
**Southern Balkan subgroup**	11	2.600 (0.000 – 5.467)	0.000 (0.000 – 3.799)	4.423 (1.864 – 99999.000)	0.19916*	0.040	0.733*	0.000	-0.726	0.268	0.364	0.594

NCESS = Northern Central European and Scandinavian Subgroup; SCES = Southern Central European Subgroup; n = sample size; Parameters of the model of population expansion (95% confidence interval): τ = expansion time; θ_0 = population size before expansion; θ_1 = population size after expansion. When SSD (sum of square deviation) values in the goodness-of-fit test for the mismatch distribution and raggedness index (rg) are non-significant ($P > 0.05$), the data do not deviate from the expectation of population expansion. Negative significant *D* and F_S values are expected when population expansion occurs. Significant values asterisked. The number of bootstrap replicates in the mismatch test was 100. Number of simulated samples for neutrality test was 1000.

Table S7. Values for mismatch distribution models and neutrality tests for ND4+tRNA sequences of the rarefied sampling of the northern group of the blue lineage.

Group	*n*	Mismatch distribution			*Test of goodness-of-fit*		Raggedness index		Tajima's *D*		Fu's F_S	
		τ	$θ_0$	rg	*P*	*D*	rg	*P*	*D*	*P*	F_S	*P*
**rarefied sampling 1**	33	3.000 (0.488 – 3.500)	0.000 (0.000 – 0.016)	0.141 (0.000 – 99999.000)	0.00023	0.270	0.596	0.790	-1.502*	0.035	-2.477*	0.001
**rarefied sampling 2**	33	3.000 (0.264 – 3.000)	0.000 (0.000 – 0.000)	0.066 (0.000 – 99999.000)	0.00001	0.190	0.776	0.830	-1.140*	0.030	-1.290*	0.004
**rarefied sampling 3**	33	3.000 (0.301 – 3.000)	0.000 (0.000 – 0.023)	0.066 (0.000 – 99999.000)	0.00001	0.220	0.776	0.860	-1.140*	0.037	-1.290*	0.004
**rarefied sampling 4**	33	3.000 (0.537 – 3.000)	0.000 (0.000 – 0.005)	0.141 (0.000 – 99999.000)	0.00023	0.210	0.596	0.790	-1.502*	0.039	-2.477*	0.001
**rarefied sampling 5**	33	0.520 (0.098 – 1.086)	0.000 (0.000 – 0.065)	99999.000 (9.938 – 99999.000)	0.01052	0.140	0.187	0.200	-0.251	0.403	-0.163	0.338

n = sample size; Parameters of the model of population expansion (95% confidence interval): τ = expansion time; $θ_0$ = population size before expansion; $θ_1$ = population size after expansion. When SSD (sum of square deviation) values in the goodness-of-fit test for the mismatch distribution and raggedness index (rg) are non-significant ($P > 0.05$), the data do not deviate from the expectation of population expansion. Negative significant *D* and F_S values are expected when population expansion occurs. Significant values asterisked. The number of bootstrap replicates in the mismatch test was 100. Number of simulated samples for neutrality test was 1000.

Table S8. Values for mismatch distribution models and neutrality tests for ND4+tRNA sequences of the rarefied sampling of the northern group of the yellow lineage.

Group	*n*	Mismatch distribution			*Test of goodness-of-fit*		Raggedness index		Tajima's *D*		Fu's F_S	
		τ	θ_0	rg	*P*	*D*	rg	*P*	*D*	*P*	F_S	*P*
**rarefied sampling 1**	27	0.842 (0.342 – 1.504)	0.000 (0.000 – 0.111)	99999.000 (4.762 – 99999.000)	0.01468	0.070	0.150	0.080	-1.550*	0.037	-4.179*	0.001
**rarefied sampling 2**	27	3.000 (0.258 – 3.500)	0.000 (0.000 – 0.007)	0.407 (0.000 – 99999.000)	0.00509	0.380	0.271	0.650	-1.889*	0.010	-4.199*	0.000
**rarefied sampling 3**	27	2.980 (0.000 – 43.980)	0.000 (0.000 – 7.489)	3.600 (0.407 – 99999.000)	0.22320	0.150	0.208	0.230	-1.307	0.080	-2.057*	0.020
**rarefied sampling 4**	27	0.617 (0.104 – 1.172)	0.000 (0.000 – 0.193)	99999.000 (3.697 – 99999.000)	0.00646	0.220	0.141	0.370	-1.965*	0.008	-5.668*	0.000
**rarefied sampling 5**	27	0.521 (0.000 – 1.326)	0.000 (0.000 – 0.193)	99999.000 (4.771 – 99999.000)	0.00416	0.400	0.162	0.350	-1.856*	0.010	-4.582*	0.000

n = sample size; Parameters of the model of population expansion (95% confidence interval): τ = expansion time; θ_0 = population size before expansion; θ_1 = population size after expansion. When SSD (sum of square deviation) values in the goodness-of-fit test for the mismatch distribution and raggedness index (rg) are non-significant ($P > 0.05$), the data do not deviate from the expectation of population expansion. Negative significant *D* and F_S values are expected when population expansion occurs. Significant values asterisked. The number of bootstrap replicates in the mismatch test was 100. Number of simulated samples for neutrality test was 1000.

Table S9. Values for mismatch distribution models and neutrality tests for ND4+tRNA sequences of the rarefied sampling of the northern Central European and Scandinavian subgroup (yellow lineage).

Group	*n*	Mismatch distribution			*Test of goodness-of-fit*		Raggedness index		Tajima's *D*		Fu's F_S	
		τ	θ_0	rg	*P*	*D*	rg	*P*	*D*	*P*	F_S	*P*
**rarefied sampling 1**	165	0.500 (0.369 – 1.172)	0.000 (0.000 – 0.049)	99999.000 (16.754 – 99999.000)	0.00225	0.240	0.159	0.360	-2.264*	0.000	-22.408*	0.000
**rarefied sampling 2**	165	0.482 (0.277 – 1.006)	0.000 (0.000 – 0.149)	99999.000 (13.141 – 99999.000)	0.00384	0.130	0.179	0.350	-2.012*	0.003	-14.611*	0.000
**rarefied sampling 3**	165	3.000 (0.000 – 4.074)	0.000 (0.000 – 0.009)	0.560 (0.000 – 99999.000)	0.00919	0.440	0.185	0.740	-2.149*	0.000	-16.834*	0.000
**rarefied sampling 4**	165	0.609 (0.471 – 1.156)	0.000 (0.000 – 0.155)	99999.000 (19.547 – 99999.000)	0.00543	0.080	0.139	0.110	-2.294*	0.000	-27.715*	0.000
**rarefied sampling 5**	165	0.553 (0.223 – 1.059)	0.012 (0.000 – 0.070)	99999.000 (20.133 – 99999.000)	0.00212	0.310	0.135	0.370	-2.300*	0.000	-25.324*	0.000

n = sample size; Parameters of the model of population expansion (95% confidence interval): τ = expansion time; θ_0 = population size before expansion; θ_1 = population size after expansion. When SSD (sum of square deviation) values in the goodness-of-fit test for the mismatch distribution and raggedness index (rg) are non-significant ($P > 0.05$), the data do not deviate from the expectation of population expansion. Negative significant *D* and F_S values are expected when population expansion occurs. Significant values asterisked. The number of bootstrap replicates in the mismatch test was 100. Number of simulated samples for neutrality test was 1000.

Table S10. Values for mismatch distribution models and neutrality tests for ND4+tRNA sequences of the rarefied sampling of the northern group of the red lineage.

Group	*n*	Mismatch distribution			*Test of goodness-of-fit*		Raggedness index		Tajima's *D*		Fu's F_S	
		τ	θ_0	rg	*P*	*D*	rg	*P*	*D*	*P*	F_S	*P*
**rarefied sampling 1**	11	3.314 (0.000 – 3.314)	0.113 (0.000 – 0.236)	0.450 (0.000 – 99999.000)	0.07565	0.050	0.736	0.430	-1.430*	0.027	0.507	0.392
**rarefied sampling 2**	11	3.314 (0.416 – 3.314)	0.113 (0.000 – 0.014)	0.450 (0.000 – 99999.000)	0.07565	0.080	0.736	0.350	-1.430*	0.026	0.507	0.396
**rarefied sampling 3**	11	2.734 (0.000 – 6.174)	0.000 (0.000 – 0.742)	1.863 (0.153 – 99999.000)	0.02067	0.530	0.094	0.720	-0.347	0.389	-0.290	0.378
**rarefied sampling 4**	11	2.912 (0.000 – 24.912)	0.002 (0.000 – 1.086)	1.110 (0.000 – 99999.000)	0.16226	0.180	0.699	0.200	0.851	0.849	2.011	0.788
**rarefied sampling 5**	11	2.982 (0.000 – 87.982)	0.900 (0.000 – 5.989)	3.600 (0.416 – 99999.000)	0.26335	0.130	0.667	0.100	-0.127	0.379	1.454	0.691

n = sample size; Parameters of the model of population expansion (95% confidence interval): τ = expansion time; θ_0 = population size before expansion; θ_1 = population size after expansion. When SSD (sum of square deviation) values in the goodness-of-fit test for the mismatch distribution and raggedness index (rg) are non-significant ($P > 0.05$), the data do not deviate from the expectation of population expansion. Negative significant *D* and F_S values are expected when population expansion occurs. Significant values asterisked. The number of bootstrap replicates in the mismatch test was 100. Number of simulated samples for neutrality test was 1000.

Table S11. Values for mismatch distribution models and neutrality tests for ND4+tRNA sequences of the rarefied sampling of the Carpathian Basin subgroup of the red lineage.

Group	*n*	Mismatch distribution			*Test of goodness-of-fit*		Raggedness index		Tajima's *D*		Fu's F_S	
		τ	θ_0	rg	*P*	*D*	rg	*P*	*D*	*P*	F_S	*P*
**rarefied sampling 1**	11	2.355 (0.000 – 4.496)	0.000 (0.000 – 0.656)	7.129 (1.773 – 99999.000)	0.00824	0.630	0.044	0.890	-0.586	0.349	-1.923	0.060
**rarefied sampling 2**	11	2.975 (0.000 – 4.234)	0.002 (0.000 – 0.018)	1.182 (0.000 – 99999.000)	0.16210	0.110	0.662	0.080	-0.932	0.196	0.624	0.601
**rarefied sampling 3**	11	2.350 (0.816 – 4.131)	0.000 (0.000 – 0.571)	10.200 (3.338 – 99999.000)	0.00474	0.840	0.033	0.840	0.494	0.700	-0.483	0.328
**rarefied sampling 4**	11	2.197 (0.000 – 4.572)	0.007 (0.000 – 2.784)	4.339 (0.153 – 99999.000)	0.03505	0.320	0.118	0.550	0.238	0.674	0.058	0.522
**rarefied sampling 5**	11	2.855 (0.000 – 4.488)	0.000 (0.000 – 0.779)	2.194 (0.000 – 99999.000)	0.09072	0.160	0.325	0.110	-0.648	0.278	0.003	0.478

n = sample size; Parameters of the model of population expansion (95% confidence interval): τ = expansion time; θ_0 = population size before expansion; θ_1 = population size after expansion. When SSD (sum of square deviation) values in the goodness-of-fit test for the mismatch distribution and raggedness index (rg) are non-significant ($P > 0.05$), the data do not deviate from the expectation of population expansion. Negative significant *D* and F_S values are expected when population expansion occurs. Significant values asterisked. The number of bootstrap replicates in the mismatch test was 100. Number of simulated samples for neutrality test was 1000.

Table S12. Genetic diversities for ND4+tRNA of the two star-like haplotype-clusters of the red lineage; r3 and r4 are the central haplotypes.

Group	*n*	*S*	*h*	h_P	*Hd*	π (*10^{-3})
**Cluster "r3"**	226	12	11	11	0.177	0.26
**Cluster "r4"**	217	25	22	22	0.362	0.61

n = sample size; *S* = number of segregating sites; *h* = number of haplotypes; h_P = number of private haplotypes; *Hd* = Haplotype diversity; π = nucleotide diversity.

Table S13. Values for mismatch distribution models and neutrality tests for ND4+tRNA sequences of the two star-like haplotype clusters of the red lineage; r3 and r4 are the central haplotypes.

**Group**	***n***	**Mismatch distribution**			***Test of goodness-of-fit***		**Raggedness index**		**Tajima's *D***		**Fu's F_S**	
		**τ**	**θ_0**	**rg**	***P***	***D***	**rg**	***P***	***D***	***P***	**F_S**	***P***
**Cluster "r3"**	226	3.000 (0.361 – 3.500)	0.000 (0.000 – 0.025)	0.211 (0.000 – 99999.000)	0.00009	0.420	0.482	0.660	-2.126*	0.000	-14.855*	0.000
**Cluster "r4"**	217	3.000 (0.000 – 5.076)	0.000 (0.000 – 0.217)	0.576 (0.009 – 99999.000)	0.00102	0.690	0.192	0.690	-2.400*	0.000	-30.406*	0.000

n = sample size; Parameters of the model of population expansion (95% confidence interval): τ = expansion time; θ_0 = population size before expansion; θ_1 = population size after expansion. When SSD (sum of square deviation) values in the goodness-of-fit test for the mismatch distribution and raggedness index (rg) are non-significant ($P > 0.05$), the data do not deviate from the expectation of population expansion. Negative significant *D* and F_S values are expected when population expansion occurs. Significant values asterisked. The number of bootstrap replicates in the mismatch test was 100. Number of simulated samples for neutrality test was 1000.

References

Cornuet, J. M. *et al.* DIYABC v2. 0: a software to make approximate Bayesian computation inferences about population history using single nucleotide polymorphism. DNA sequence and microsatellite data. *Bioinformatics* **30**, 1187–1189 (2014).

Falush, D., Stephens, M. & Pritchard, J. K. Inference of population structure using multilocus genotype data: linked loci and correlated allele frequencies. *Genetics* **164**, 1567–1587 (2003).

Kindler, C. *et al*. Mitochondrial phylogeography, contact zones and taxonomy of grass snakes (*Natrix natrix, N. megalocephala*). *Zool. Scr*. **42**, 458–472 (2013).

Kindler, C., Bringsøe, H. & Fritz, U. Phylogeography of grass snakes (*Natrix natrix*) all around the Baltic Sea: implications for the Holocene colonization of Fennoscandia. *Amphibia-Reptilia* **35**, 413–424 (2014).

Kindler, C. *et al.* Hybridization patterns in two contact zones of grass snakes reveal a new Central European snake species. *Sci. Rep*. **7**, 7378 (2017).

Kindler, C. *et al*. Phylogeography of the Ibero-Maghrebian red-eyed grass snake (*Natrix astreptophora*). *Org. Divers. Evol*., 10.1007/s13127-017-0354-2 (2018).

Pokrant, F. *et al.* Integrative taxonomy provides evidence for the species status of the Ibero-Maghrebian grass snake *Natrix astreptophora*. *Biol. J. Linn. Soc.* **118**, 873–888 (2016).

Pritchard, J. K., Stephens, M. & Donnelly, P. Inference of population structure using multilocus genotype data. *Genetics* **155**, 945–959 (2000).
